# Abstracts of the 22th European Conference on Eye Movements, 25-29 August 2024, in Maynooth (Irland)

**DOI:** 10.16910/jemr.17.6.1

**Published:** 2024-12-22

**Authors:** Ralph Radach, Ronan Reilly

**Affiliations:** General and Biological Psychology, University of Wuppertal; Department of Computer Science, Maynooth University

**Keywords:** eye movements, perception, attention, reading, microsaccades, gaze

## Abstract

This document contains all abstracts of the 22th European Conference on Eye Movements, August 25-29, 2024, in Maynooth, Irland

## Abstract Book ECEM 2024



