# Supplementary material for: Abstracts of the 22th European Conference on Eye Movements, 25-29 August 2024, in Maynooth (Irland)
Source: J Eye Mov Res. 2024 Dec 22;17(6):10.16910/jemr.17.6.1. doi: 10.16910/jemr.17.6.1 (PMC11787908; doi:10.16910/jemr.17.6.1)
Supplement: Supplementary file 1 [file jemr-17-06-a-SD1-01.pdf]

ECEM

22nd European Conference on Eye Movements

August 25-29, 2024

Maynooth University, Ireland

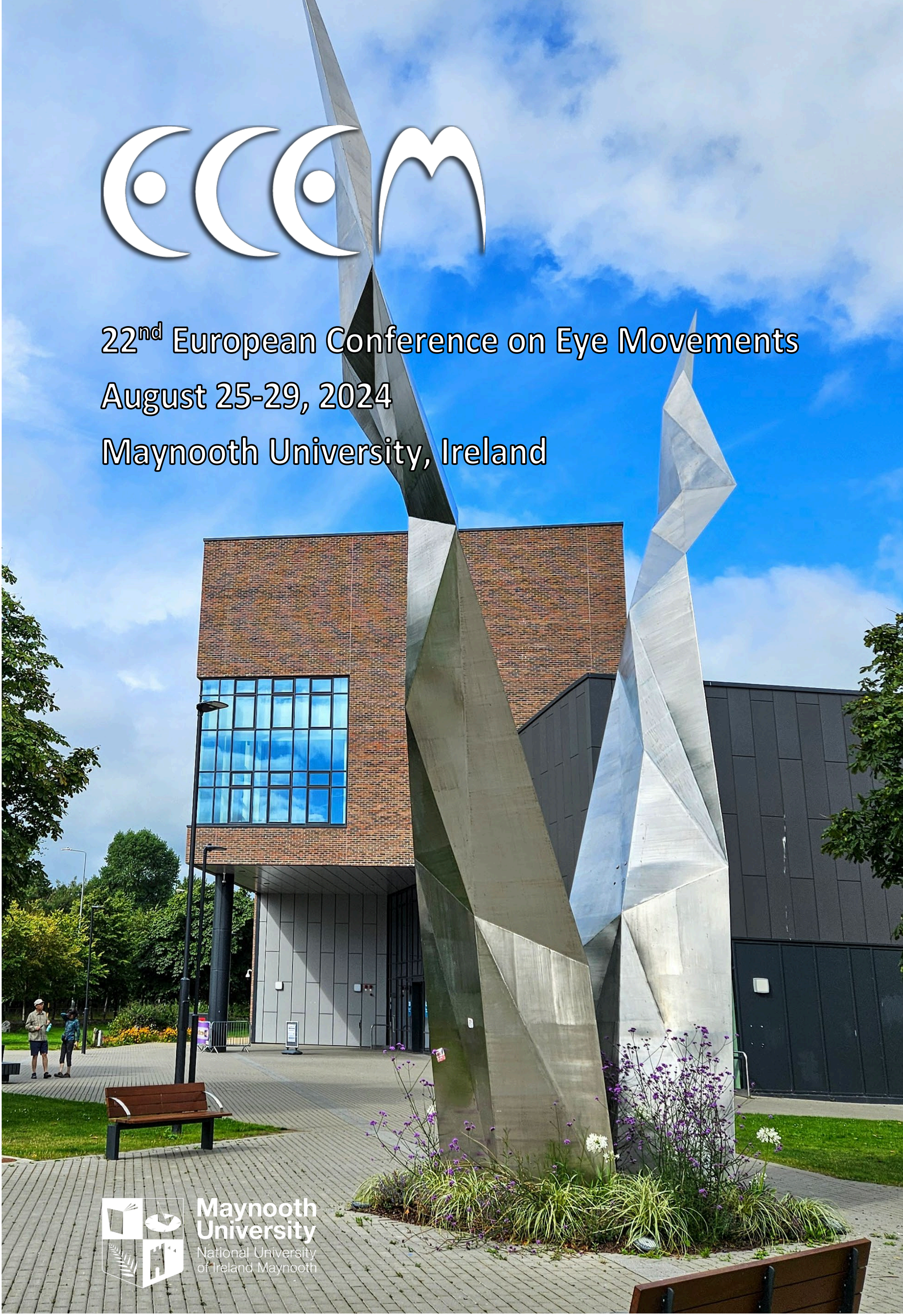

Maynooth University
National University of Ireland Maynooth

ECEM 2024

Abstracts

August 25-29, 2024

Maynooth University, Ireland

Edited by Ronan Reilly & Ralph Radach

Contents

Conference information 5
Keynote Speakers 6
Conference schedule 11
Keynotes 40
Talks 44
  Monday morning 44
    Symposium 1: Crosslinguistic Explorations 44
    Visual Perception and Saccade Control 47
    Symposium 2: Understanding Interaction 50
  Monday afternoon 52
    Visual Processing and Eye Movement Control in Reading 52
    Neural Basis of Eye Movements 56
    Oculomotor Measurement Issues 59
  Tuesday morning 62
    Parafoveal processing in reading 62
    Eye Movement Data Analyses 65
    Eye Tracking in (near) Natural Settings 68
  Tuesday afternoon 71
    Word Processing in Reading 71
    Clinical Eye Movement Research 74
    Eye Movements in the Study of Language 77
  Wednesday morning 80
    Reading Development 80
    Eye Movements in Social Interaction 83
    Visual Processing and Cognitive Control 86

Wednesday afternoon 89
High-level Processing in Reading 89
Applications in Communication and Sport Science 92
Individual Differences and Special Populations in Reading 96
Thursday morning 99
Image and Scene Perception 99
Reading in Different Languages 102
Problem Solving and Expertise 105
Posters 108
Session 1 108
Session 2 127

Conference Information

Conference chairs:

Ralph Radach (Programme)
General and Biological Psychology, University of Wuppertal

Ronan Reilly (Local organisation)
Department of Computer Science, Maynooth University

Programme Committee

Ralph Radach, University of Wuppertal, Germany (chair)
Ignace Hooge, Utrecht University, Netherlands
Marcus Nyström, Lund University, Sweden
Andreas Sprenger, University of Lübeck, Germany
Lizz Schotter, University of South Florida, USA
Johanna Kaakinen, University of Turku, Finland
Lynn Huestegge, University of Würzburg, Germany
Chuanli Zang, University of Central Lancashire, England
Victor Kuperman, McMaster University, Canada
Denis Driehge, University of Southampton, England
Doug Munoz, Queens University, Canada

ECEM Scientific Committee

Ulrich Ansorge (Wien, Austria)
Ralph Engbert (Potsdam, Germany)
Rudolf Groner (Bern, Switzerland)
Jukka Hyönä (Turku, Finland)
Simon Liversedge (Preston, England)
Susana Martinez-Conde (New York, USA)
Wayne Murray (Dundee, Scotland)
Kevin Paterson (Leicester, England)
Ralph Radach (Wuppertal, Germany)
Francoise Vitu (Marseille, France)
Daniela Zambarbieri (Pavia, Italy)

Organising Team

Sinead Marrinan (coordination)
Precious Deremo, Seán Patrick Levey, Mark McCormack, Yulo Ou,
Sabine Petzold, Ananya Rajora, Lavanya Rajora, Wanshi Zhang

Keynote Speakers

We are pleased to present the following keynote speakers at ECEM 2024:

- Susana Chung, UC Berkeley
- Hans Gellersen, Lancaster University, UK
- Jacqueline Gottlieb, Columbia University, New York
- Xinshang Li, Chinese Academy of Sciences
- Alexander Schütz, Marburg University, Germany

Susana Chung
UC Berkeley

Fixational eye movements in the absence of central vision

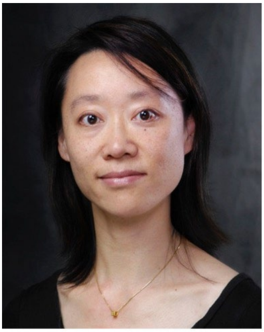

Susana Chung is a Professor of Optometry and Vision Science at the University of California, Berkeley. She completed her optometry training at the Hong Kong Polytechnic University, and subsequently received a MSc in Optometry degree from the University of Melbourne and a PhD in Physiological Optics from the University of Houston. She then completed her post-doctoral training at the University of Minnesota.

The major goals of Susana's research center on the understanding of the limiting factors on vision in the presence of eye disorders or diseases, and whether effective paradigms could be developed to improve vision for people with impaired vision. She uses a variety of techniques including psychophysics, computational modeling, retinal imaging, and eye tracking in her research. Her research has been continuously supported by NIH since 2000. Susana has received several awards for her contribution to research, including the Atwell Award for Research Excellence in Low Vision, the Borish Outstanding Young Researcher Award, and the Glenn A. Fry Award. Currently, she is on the editorial board of Vision Research, Journal of Vision, Optometry and Vision Science and Frontiers of Neuroscience.

Hans Gellersen
Lancaster University, UK

Gaze and Eye Movement as Input for Human-Computer Interaction

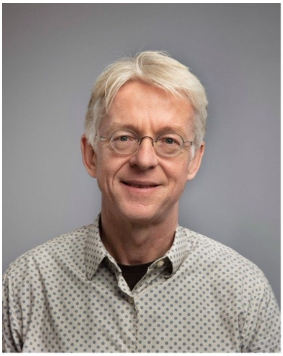

Hans Gellersen is Professor of Interactive Systems at Lancaster University and Aarhus University. His background is in Computer Science with research interests is in sensors and devices for ubiquitous computing and human-computer interaction. He has worked on systems that blend physical and digital interaction, methods that infer context and human activity, and techniques that facilitate spontaneous interaction across devices.

In his recent work, he is focussing on multimodal interaction techniques that leverage gaze and eye movement, supported by a prestigious ERC Advanced Grant awarded by the European Research Council.

Jacqueline Gottlieb
Columbia University, New York

Allocating attention for information gain: the roles of uncertainty, information diagnosticity and cognitive costs

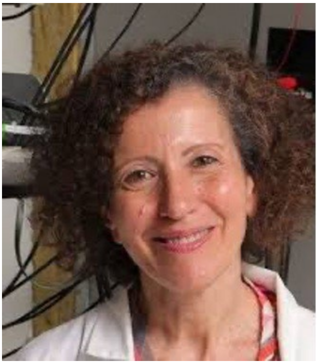

Jacqueline Gottlieb studies the mechanisms that underlie the brain's higher cognitive functions, including decision making, memory, and attention. Her interest is in how the brain gathers the evidence it needs — and ignores what it doesn't — during everyday tasks and during special states such as curiosity. Her research could offer insight into disorders that involve deficits of attention, such as attention deficit disorder, depression and drug addiction.

Xinshang Li
Chinese Academy of Sciences, Beijing

Canyoureadthis? The critical but overlooked role of word-segmentation during reading

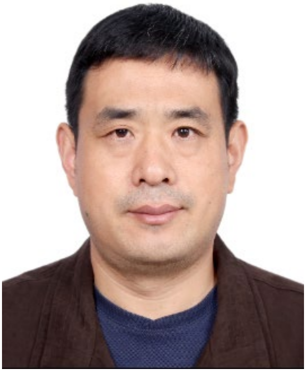

Xingshan Li is a Professor of Psychology at the Institute of Psychology, Chinese Academy of Sciences. He earned his Ph.D. degree in Psychology from the University of Massachusetts Amherst (2007). His research focuses on Chinese reading and language processing. In recent years, he has delved into understanding how Chinese readers deal with the unique features of Chinese text during reading, employing techniques such as eye tracking and computational modeling.

Notably, he has proposed innovative perspectives on word segmentation and saccade target selection by Chinese readers, even in the absence of inter-word spaces. His work culminated in the development of a computational model that simulates eye-movement control and word processing during Chinese reading. Li has an impressive publication record, with approximately 100 journal articles published in esteemed journals such as Psychological Review, Nature Reviews Psychology, JEP: General, and Cognitive Psychology. Additionally, he serves as an Associate Editor for the Quarterly Journal of Experimental Psychology. His contributions to the field have been recognized through various honors and awards, including the Best Article Award from the Psychonomics Society. His dedication extends to academic service as well, as he actively participates in editorial boards and conferences related to eye movements and cognitive psychology.

Alexander Schütz
University of Marburg, Germany

Transsaccadic integration of peripheral and foveal feature information

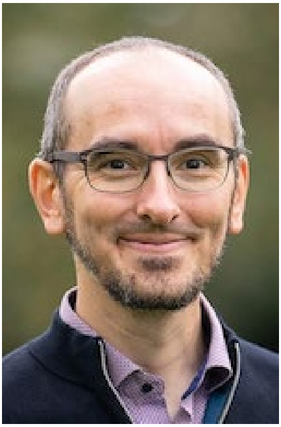

Alexander Schütz is a Professor for Experimental Psychology at Marburg University. He studied Psychology at Erlangen University and got interested in visual perception during his undergraduate thesis on the appearance of warning lights in cars. Eye movements came into play when he obtained a PhD in Karl Gegenfurtner's lab at Gießen University, on the modulation of contrast sensitivity by smooth pursuit eye movements. During his post-doc, he expanded his research interests on saccadic eye movements and visited Dirk Kerzel's lab in Geneva and Concetta Morrone's lab in Pisa. He is interested in the perception-action cycle of active vision, i.e., how visual and cognitive signals guide our eyes and how eye movements determine what we see. He received an ERC Starting Grant on the interaction of peripheral and foveal vision across saccadic eye movements and subsequently an ERC Consolidator Grant on the interplay of sensory and inferred signals in different types of perceptual completion. He uses eye tracking, psychophysics, computational modeling and EEG in his research.

Conference schedule

Keynotes

Monday (9.00 - 10.00)

Gaze and Eye Movement as Input for Human-Computer Interaction
Hans Gellersen

Tuesday (9.00 - 10.00)

Transsaccadic integration of peripheral and foveal feature information
Alexander Schütz

Wednesday (9.00 - 10.00)

Canyoureadthis? The critical but overlooked role of word-segmentation during reading
Xinshang Li

Thursday (9.00 - 10.00)

Allocating attention for information gain: the roles of uncertainty, information diagnosticity and cognitive costs
Jacqueline Gottlieb

Thursday (13:30 - 14.30)

Fixational eye movements in the absence of central vision
Sandra Chung

Talks

**Sunday - Evening - Conference Opening - Venue A**		
15:00 - 18:00	Registration	
18:00 - 19:00	Reilly, Ronan<br>Radach, Ralph	Welcome address.
19:00 - 21:00	Welcome reception	
**Monday - morning - Keynote 1 - Venue A**		
09:00 - 10:00	Gellersen, Hans	Gaze and Eye Movement as Input for Human-Computer Interaction
**10:00 - 10:30**	**Coffee break**	
**Monday - morning - Symposium 1: Crosslinguistic Explorations - Venue A**<br>**Chair: Victor Kuperman**		
10:30 - 10:50	Kuperman, Victor	Presenting Wave 2 of the MulAlingual Eye Movement Corpus (MECO)
10:50 - 11:10	Hyönä, Jukka	Universality in eye movements and reading: A replication with increased power
11:10 - 11:30	Schwalm, Laura	What is universal in eye movement control during reading? Landing Positions of Regressive and Progressive Saccades in 13 languages

11:30 - 11:50	Häikiö, Tuomo	Eye movements of children and adults reading in three different orthographies: The effects of the past, present, and future words
11:50 - 12:10	Schroeder, Sascha	Eye Movements of adults reading three different orthographies as a second language
12:10 - 12:30	Drieghe, Denis	Individual Differences in Word Skipping During Reading in English as L2

Monday - morning - Visual Perception and Saccade Control - Venue B
Chair: Eugene McSorley

10:30 - 10:50	Cambronero Delgadillo, Alejandro Javier	When Time Disrupts: Interruptions in Visual Searches
10:50 - 11:10	Crawford, John Douglas	Influence of Saccades on Cortical Modularity During a Feature Discrimination Task
11:10 - 11:30	Kirkpatrick, Ryan Hannah	Saccades, blinks and pupil responses in youth with an eating disorder: Findings from a multi-site Canadian study using an interleaved pro-saccade/anti-saccade task
11:30 - 11:50	McSorley, Eugene	Sequences of eye movements: the role of the Gestalt
11:50 - 12:10	Robbins, Arryn	Eye movements reflect improvements in search for heterogeneous categories
12:10 - 12:30	Vasilev, Martin Rachev	Unexpected sounds induce a rapid inhibition of eye-movement responses

Monday - morning - Symposium 2: Understanding Interaction - Venue C<br>Chair: Andre Duchowksi		
10:30 - 10:50	Baldisserotto, Filippo	Exploring the dynamics of ambient and focal visual attention of novices in continuous construction tasks.
10:50 - 11:10	Duchowski, Andrew Ted	Gaze Interaction in XR
11:10 - 11:30	Ekin, Merve	Predictive Model of Intrinsic Cognitive Load from Physiological Measures
11:30 - 11:50	Krejtz, Izabela	Gaze-Led Audio Description. Eye-movement data driven technology for cultural inclusion.
11:50 - 12:10	Warchoł-Jakubowska, Anna	Comparing Visual Attention Dynamics of Tram Drivers: Expertise Impact on Safety Awareness
**12:30 - 13:30**	**Lunch break**	

Monday - afternoon - Visual Processing and Eye Movement Control in Reading - Venue A<br>Chair: Francoise Vitu		
13:30 - 13:50	Bao, Yaqian	Comparative Analysis of Saccadic Main Sequences in Horizontal and Vertical Reading
13:50 - 14:10	Engbert, Ralf	Bayesian inference of the simplified SWIFT model of eye-movement control in reading
14:10 - 14:30	Li, Lin	Unpacking Word Segmentation Processes by L2 Chinese Readers: Evidence from Eye Movements
14:30 - 14:50	Parshina, Olga	Cross-linguistic comparison in reading sentences of uniform length: Visual-perceptual demands override readers' experience
14:50 - 15:10	Schotter, Elizabeth Roye	Perceptual and word identification spans do not differentially impact when and where saccade decisions
15:10 - 15:30	Vitu, Françoise	Accounting for Chinese readers' eye-movement behavior using universal visuo-motor computations in the midbrain: Evidence against a role of word segmentation

Monday - afternoon - Neural Basis of Eye Movements - Venue B<br>Chair: Doug Munoz		
13:30 - 13:50	Caziot, Baptiste	Physiological correlates of a simple saccadic-decision task to extended objects in superior colliculus
13:50 - 14:10	Eggert, Thomas	Differences in the control of conjugate and disconjugate components of horizontal fixation drift
14:10 - 14:30	Malevich, Tatiana	Sound activates a dormant visual-motor pathway bypassing primary visual cortex
14:30 - 14:50	Pitigoi, Isabell Christine	Blinks as a critical oculomotor effector: using eye-tracking and electromyography to understand spontaneous blink behaviour
14:50 - 15:10	Semmlow, John	Fusion Sustaining Oscillations: Dependence on Sustained Convergence Level
15:10 - 15:30	Zhang, Tong	A transient signal in foveal superior colliculus neurons for jumpstarting peripheral saccadic orienting

Monday - afternoon - Oculomotor Measurement Issues - Venue C<br>Chair: Ignace Hooge		
13:30 - 13:50	Angele, Bernhard	Using affordable eye tracking methods to study reading: the role of sampling rate
13:50 - 14:10	Barbara, Nathaniel	Optimal Bipolar Channel Selection for EOG-Based Gaze Displacement Estimation

14:10 - 14:30	Graz, Heather	Validation of remote eye-tracker technology as a vehicle for investigation of language comprehension processes in the absence of a stable head position
14:30 - 14:50	Hooge, Ignace T.C.	How to record 140° gaze shifts?
14:50 - 15:10	Pryslopska, Anna	A Concurrent Comparison of EyeLink 1000+ and TrackPixx3 on a Benchmark Test Battery
15:10 - 15:30	Seernani, Divya Prakash	Webcam based eye-tracking -Validation Study Report
**15:30 - 17:00**	**Coffee & Poster Session 1**	

**Tuesday - morning - Keynote 2 - Venue A**		
09:00 - 10:00	Schütz, Alexander	Transsaccadic integration of peripheral and foveal feature information
**10:00 - 10:30**	**Coffee break**	
**Tuesday - morning - Parafoveal Processing in Reading - Venue A**<br>**Chair: Simon Liversedge**		
10:30 - 10:50	Atanasov, Petar Filipov	Neural correlates of parafoveal n+1 and n+2 word processing during sentence reading
10:50 - 11:10	Heikkilä, Timo T	Relating foveal and parafoveal word processing efficiency with eye-movement measures of reading Chinese

11:10 - 11:30	Loberg, Otto Henrik	What’s up with “the”? – Co-registered ET-EEG investigation of parafoveal syntax processing.
11:30 - 11:50	Saunders, Emily	Parafoveal processing of morphological structure for deaf and hearing readers
11:50 - 12:10	Wu, Shi Hui	Parafoveal preview of short words during reading and skimming
12:10 - 12:30	Zang, Chuanli	The influence of foveal processing load on parafoveal preview extent in Chinese reading

Tuesday - morning - Eye Movement Data Analyses - Venue B
Chair: Markus Nyström

10:30 - 10:50	Culemann, Wolf	Systematic Drift Correction in Eye Tracking: Integrating Line Assignments with Implicit Recalibration
10:50 - 11:10	Larigaldie, Nathanael	eyeScrollR: A software method for reproducible mapping of eye tracking data from scrollable web pages
11:10 - 11:30	Nyström, Marcus	What is a blink? Classifying and characterizing blinks in eye openness signals
11:30 - 11:50	Surkov, Anton	EyeFeatures: the package for preprocessing, visualisation, statistical and machine learning analysis of eye movement data
11:50 - 12:10	Van Hoecke, Senne M.	Analyzing large mobile eye tracking datasets: An example from educational research

12:10 - 12:30	Vojtechovska, Michaela	Streamlining Scarf Plot Generation for Eye-Tracking Research
**Tuesday - morning - Eye Tracking in (near) Natural Settings - Venue C<br>Chair: TBD**		
10:30 - 10:50	Jarodzka, Halskza	Yarbus Revisited: The Webcam Edition
10:50 - 11:10	Colucci, Livia	Pupil-driven music machine
11:10 - 11:30	Cubero Dudinskaya, Emilia	Navigating the Sea of Eco-Labels: Unveiling Consumer Attention through Eye-Tracking Analysis on Italian Aquaculture Products
11:30 - 11:50	Dreneva, Anna	Consumer search patterns in online retailing
11:50 - 12:10	Matulewski, Jacek	Usability verification of several gaze control methods of the Breakout game
12:10 - 12:30	Zemblys, Raimondas	Impact of data quality on driving performance related eye-tracking measures
**12:30 - 13:30**	**Lunch break**	

Tuesday - afternoon - Word Processing in Reading - Venue A
Chair: Kevin Paterson

13:30 - 13:50	Bellerby, Emily J.	The effects of reading ability and vocabulary on incidental word learning during reading: An eye movement investigation.
13:50 - 14:10	McGowan, Victoria A.	Do readers not fully process “not”? An eye movement investigation of the processing of negated statements.
14:10 - 14:30	Paterson, Kevin	Eye Movements and Noisy-Channel Inference-Making during Reading
14:30 - 14:50	Serrano-Carot, Marina	Article-noun agreement in the parafovea does not affect skipping in Spanish.
14:50 - 15:10	Munguba Vieira, Joao Marcos	The processing of the definite article in Brazilian Portuguese: When “the” carries gender and number marking
15:10 - 15:30	Wong, Roslyn	Predictability effects in Chinese reading: Evidence from eye movements during corpus reading

Tuesday - afternoon - Clinical Eye Movement Research - Venue B
Chair: Andreas Sprenger

13:30 - 13:50	Brien, Donald Christopher	Eye-tracking During Naturalistic Free Viewing Allows for Highly Specific Classification of Parkinson’s Disorder

13:50 - 14:10	Coubard, Olivier A.	Attention deficit and hyperactivity disorder disrupts selective mechanisms of action as revealed by eye movements
14:10 - 14:30	Noyes, Blake K	Identifying an eye movement phenotype for major depressive disorder
14:30 - 14:50	Riek, Heidi C.	Cross-task patterns of saccade abnormality in Parkinson’s disease suggest multiple affected oculomotor processes
14:50 - 15:10	Sprenger, Andreas	Oculomotor abnormalities indicate early executive dysfunction in prodromal X-linked dystonia-parkinsonism (XDP): evidence for pre-symptomatic screening of Morbus Parkinson
15:10 - 15:30	Wang, Yiting	Identifying early-stage Parkinson's Disease from Eye Movements During Steady State Fixation

Tuesday - afternoon - Eye Movements in the Study of Language - Venue C
Chair: Johanna Kaakinen

13:30 - 13:50	Diao, Linghui	Leveraging the Visual World Eye-Tracking Paradigm in Analyzing Second Language Pronoun Processing
13:50 - 14:10	Han, Haibin	A transposed-syllable effect in Chinese spoken word recognition
14:10 - 14:30	Kaakinen, Johanna Karoliina	Shifts in attention during listening of a novel: Evidence from eye tracking

14:30 - 14:50	Kanerva, Oksana	Form-meaning iconicity facilitates semantic recognition of onomatopoeic words: Evidence from eye movements
14:50 - 15:10	Lopukhina, Anastasiya	Where do children look when watching videos with same-language subtitles?
15:10 - 15:30	Luo, Yuhan	Pupillary responses during extended listening in L2 learners of English
**15:30 - 16:00**	**Coffee**	

Wednesday - morning - Keynote 3 - Venue A

09:00 - 10:00	Li, Xinshang	Canyoureadthis? The critical but overlooked role of word-segmentation during reading
**10:00 - 10:30**	**Coffee break**	

Wednesday - morning - Reading Development - Venue A
Chair: Christopher Lonigan

10:30 - 10:50	Chevet, Guillaume	Effect of interruptions during reading on comprehension and eye movements in children
10:50 - 11:10	Dostálová, Nicol	Eye movements when reading pseudo-text in dyslexic children: Evidence from eye tracking

11:10 - 11:30	Günther, Thomas	Decoding Gender in German: Insights from Eye-Tracking Experiments on Language Comprehension in Children with Developmental Language Delay
11:30 - 11:50	Loningan, Christopher	Predicting young children's eye movements during reading from teachers' ratings of inattention
11:50 - 12:10	Milledge, Sara Victoria	Characterising Children's Eye Movement Control During Reading in English: A Corpus Study
12:10 - 12:30	Spichtig, Alexandra	Reading Efficiency, Academic Performance, and Visual Comfort: Exploring a Hierarchical Model

Wednesday - morning - Eye Movements in Social Interaction - Venue B
Chair: Lynn Huestegge

10:30 - 10:50	Mayrand, Florence	Mutual gaze in social interactions: The effects of collaboration and competition
10:50 - 11:10	Hessels, Roy	A setup for the cross-cultural study of gaze behavior and eye contact in face-to-face collaboration
11:10 - 11:30	Hoffmann, Alexandra	Dual mobile eye-tracking during social interactions to predict problem-solving performance & decision-making behaviour
11:30 - 11:50	Huestegge, Lynn	Semantics of gaze in person perception: A novel qualitative-quantitative approach
11:50 - 12:10	Cakir, Mehtap	Eye Contact Modulates Eyeblink Synchronization and Rapport

12:10 - 12:30	Valtakari, Niilo	Infant action prediction and gaze behavior in interaction with their parents

**Wednesday - morning - Visual Processing and Cognitive Control - Venue C**<br>**Chair: Ronan Reilly**		
10:30 - 10:50	Del Sordo, Giovanna Carosena	Understanding behavioral regulation: How the eyes reveal exploration-exploitation dynamics
10:50 - 11:10	Foucher, Valentin	Deceptive Intentions: Insights from Eye Movements in a Card Game Experiment
11:10 - 11:30	Nikolaev, Andrey	Deciphering episodic memory encoding and retrieval: a glimpse through eye movements and EEG
11:30 - 11:50	Orquin, Jacob L.	Covert Attention Leads to Fast and Accurate Decision-Making
11:50 - 12:10	Papesh, Megan H	Using Context to Improve Prospective Memory: A Pupillometry Study
12:10 - 12:30	Wolf, Christian	Purposive engagement is crucial for eliciting oculomotor markers of effort
**12:30 - 13:30**	**Lunch break**	

Wednesday - afternoon - High-level processing in Reading - Venue A<br>Chair: Jaana Simola		
13:30 - 13:50	Covarrubias, Santana	The effect of the internal characteristics of coherence relations on their processing and comprehension
13:50 - 14:10	Lee, Charlotte	Reading Comprehension Tests and the Jingle Fallacy: Implications for Eye Movement Research
14:10 - 14:30	Liceralde, Van Rynald T.	Overall effects and item-level measures port well across eye-tracking and self-paced reading but participant-specific effects port poorly
14:30 - 14:50	Mézière, Diane	Eye movements from a ‘read-only’ task predict reading comprehension as measured by recall
14:50 - 15:10	Olkoniemi, Henri	Role of Empathy in Children’s Processing and Comprehension of Written Irony
15:10 - 15:30	Simola, Jaana	The effect of state curiosity on eye movements during reading of health-related arguments

Wednesday - afternoon - Applications in Communication and Sport Science - Venue B<br>Chair: Chin-An Wang		
13:30 - 13:50	Castner, Nora Jane	Gaze Behavior of Dentists Inspecting Bitewings with an AI-Support Tool: Implications for Expert Diagnostic Strategies and AI Usability
13:50 - 14:10	Chen, Jiemiao	Children’s eye gaze during a face-to-face conversation: Effects of social anxiety and conversational role?

14:10 - 14:30	Neukirchen, Tobias	Hungry Eyes: Linking Food Cue Processing and Cognitive Glucose Sensitivity
14:30 - 14:50	Sciarra, Dalila	Dynamical Accommodation of Overt Attention, Accuracy and Time in Modern Pentathlon Athletes during Laser Run Shooting
14:50 - 15:10	Wang, Chin-An	Saccade latency and metrics in the interleaved pro- and anti-saccade task in athletes
15:10 - 15:30	Zeka, Fatime	iSCAN project: Examination of gaze behavior in social anxiety disorder using a virtual reality eye-tracking paradigm: a case–control study

Wednesday - afternoon - Individual Differences and Special Populations in Reading - Venue C
Chair: Karen Emmorey

13:30 - 13:50	Cooley, Frances Grosvenor	A corpus study of length, frequency, and surprisal effects in deaf and hearing readers
13:50 - 14:10	Egan, Ciara	Semantic and attentional processing during reading in adults with dyslexia
14:10 - 14:30	Emmorey, Karen	The unique eye movement profile of deaf readers reveals the plasticity of the reading system
14:30 - 14:50	Moncada, Fernando	Exploring the impact of individual differences and reading goals on eye movements and comprehension of academic texts

14:50 - 15:10	Wang, Jingxin	“老马识途” (“An Old Horse Knows the Direction”): Older Adults have Greater Difficulty Reading Transposed Chinese Idioms
15:10 - 15:30	Whyatt, Boguslawa	The role of text- and reader-related factors in narrative engagement when reading translated fiction: An eye-tracking study
**15:30 - 17:00**	**Coffee & Poster Session 2**	
**19:00 - 22:00**	**Conference Dinner at Barberstown Castle**	

Thursday - morning - Keynote 4 - Venue A

09:00 - 10:00	Gottlieb, Jacqueline	Allocating attention for information gain: the roles of uncertainty, information diagnosticity and cognitive costs
**10:00 - 10:30**	**Coffee break**	

Thursday - morning - Image and Scene Perception - Venue A
Chair: Jochen Laubrock

10:30 - 10:50	Farhangpour, Yasaman	Evaluating Emotional Perception in Qajar and Neoclassical Portraits: An Eye-tracking Approach

10:50 - 11:10	Foulsham, Tom	Does context matter? (Minimal) effects of minimal context on eye movements in scene viewing
11:10 - 11:30	Laubrock, Jochen	Reading scenes: Evidence for narrative guidance of visual attention in scene perception
11:30 - 11:50	Pertzov, Yoni	Unraveling Gaze Dynamics Towards Familiar Items: Implications and Insights
11:50 - 12:10	Popelka, Stanislav	Exploring visitor engagement through eye-tracking analysis at a science exhibition
12:10 - 12:30	Værnes, Bernard Matthew	All Eyes on The Cyber Canvas: Expert and Non-expert Online Viewing Patterns, Preferences and Memory of AI and Human Paintings

Thursday - morning - Reading in Different Languages - Venue B
Chair: Xinshang Li

10:30 - 10:50	AlJassmi, Maryam A.	The Role of Ligatures in Arabic Reading: Evidence from Eye Movements
10:50 - 11:10	Azeez, Rizwana	Lexical Processing in Urdu: An eye tracking study
11:10 - 11:30	Hodgins, Vegas Anton	Eye-Tracking Measures of Bilingual Irony Processing: A Within-Participant Look at L1 vs. L2 Reading Effects
11:30 - 11:50	Guan, Zheng-Hong	Task Effect on Multiple-text Reading and Writing: Insights from Eye-tracking Research

11:50 - 12:10	Tarin, Karla	How do bilingual adults draw mentalizing inferences from text? An eye-tracking study of natural reading.
12:10 - 12:30	Warrington, Kayleigh L.	A Comparison of Monospaced & Proportional Fonts in Arabic Reading: Effects on Foveal and Parafoveal Processing

Thursday - morning - Problem-Solving and Expertise - Venue C
Chair: Halszka Jarodzka

10:30 - 10:50	Chaudhuri, Saswati	Joint visual attention during computer-assisted collaborative problem-solving task: Case study of two dyads with high and low similarities in visual scanpath
10:50 - 11:10	Green, Christine	Eye movements during the verification of arithmetic calculations
11:10 - 11:30	Jarodzka, Halszka	Enhancing Debugging Performance with Eye Movement Modeling Examples (EMME): An Investigation into Type, Expertise, and Engagement
11:30 - 11:50	Li, Ren-Ping	Exploring Cognitive Load During Geometry Problem Solving Processes by Integrating Eye Movement and Handwriting
11:50 - 12:10	Lin, John J. H.	Automated prediction of problem solving performance using eye movement: Can AI help?

12:10 - 12:30	van Nooijen, Christine Charlotte Anja	Face to face with an expert: Exploring joint visual attention during forensic face comparison and feature comparison in three expert-novice dyads
**12:30 - 13:30**	**Lunch break**	

**Thursday - afternoon - Keynote 5 - Venue A**		
13:30 - 14:30	Chung, Susana	Fixational eye movements in the absence of central vision

**Thursday - afternoon - Awards & Farewell - Venue A**		
14:30 - 15:30	Ronan Reilly & Ralph Radach	

Posters

Monday afternoon - Poster Session 1		
1-1	Lee, Daniel John	Is social cognition a mediator on the relationship between literature consumption and empathy-related pupil dilation?
1-2	Balcombe, Faye O.	Eye movement behaviour during reading across older adulthood
1-3	Li, Meng-Huan	The Role of Individual Differences on English Incidental Vocabulary Learning: An Eye-Tracking Study
1-4	Benedi-Garcia, Clara	Difference of fixation stability for two progressive lens designs during dynamic visual tasks
1-5	Bényei, Gábor László	Promise of open-source, low-cost pupillometry – Contribution to the PupilEXT platform
1-7	Brady, Nuala	Eye movements reveal differences in attentional processing of words and faces between dyslexic and typical readers
1-8	Camilleri, Tracey	GUI-free EOG-based control of smart devices
1-9	Chen, Ting-Ai	The Differences in Cognitive Processes and Comprehension Results of L2 Readers with Different Language Proficiency Levels: An Eye movement Study
1-10	Concepcion-Grande, Pabl	A new algorithm for identifying saccade movements in eye-tracking experiments.
1-11	Copeland, Allyson L	Individual differences in working memory and lexical quality predict eye-movements, but not fixation-related potentials: Evidence from co-registration of EEG and eye-tracking

1-12	Culemann, Wolf	Eyeflow Studio: An Extensible GUI-Based Tool for Dynamic Eye-Tracking Data Processing and Analysis
1-13	Di Stasi, Leandro L.	Monitoring arousal levels among crew members of a Spanish battleship: An eye-tracking based longitudinal observational study
1-14	Fan, Danni	Art Through the Mind's Eye: How Cognitive Styles Shape Visual Attention with Eastern and Western Masterpieces
1-15	Fan, Xi	Do Chinese readers of English find text in capitals hard to read?
1-16	Fathkhani, Sadra	Exploring the role of the macaque lateral intraparietal area in voluntary and reflexive saccadic eye movements
1-17	Finney, Hunter Christian	Effects of Pupil Swim on Eye-Tracking in Virtual Reality
1-18	Gill, Donna	Parafoveal processing of word length and compound words in English children
1-19	Guan, Zheng-Hong	Who is addicted to games? Comparison of attentional bias through eye movements among e-athletes, gaming addicted, and normal gamers
1-20	Gutzeit, Julian	Saccade Automaticity and Sense of Agency
1-21	Henning, Miyuki	Parafoveal Processing of Kanji Characters during Reading Japanese
1-22	Salmela, Rosa	Stem Alternations in Easy Language and L2 Reading Fluency

1-23	Hofmann, Markus J	Individual text corpora for predicting personality and eye-movements during reading
1-24	Holleman, Gijs	The Pupil Labs Neon for the study of face-to-face conversation: A test of data quality and comparison of AOI-analyses
1-25	Huang, Linjieqiong	The Effects of Lexical and Sentence Level Contextual Cues on Chinese Word Segmentation
1-26	Ibáñez, Romualdo	Eye movement to measure the effect of syntactic complexity and socioeconomic status on school textbooks processing.
1-27	Iniesta, Antonio	How do first and second language readers deploy theory of mind during text processing? A cross-study comparison of mentalizing inferences and irony processing.
1-28	Janßen, Sven	A framework for gaze-analysis during multimodal interactions in spatial environments
1-29	Jeppesen, Alberte Cathrine Ehrhardt	EYEdentify - Investigating Unique Eye Gaze Patterns in Adults with Autism Spectrum Disorders Using Virtual Reality
1-30	Jostrup, Erica	The effect of white noise stimulation on oculomotor control in children with ADHD
1-31	Klumova, Sofya	The role of personal traits in individual eye movement strategies during facial expression recognition
1-32	Wong, Roslyn	Testing the familiarity check assumption of E-Z Reader using concurrent eye-tracking and magnetoencephalography
1-33	Körner, Christof	Inhibition of return and saccadic momentum in a saccade sequencing paradigm

1-34	Kovacs, David-Levente	Using eye movements for the identification of partners in dyadic interactions
1-35	Kovalev, Artem	The influence of pursuit eye movements on changes in brain activity during optokinetic exposure
1-36	Kreß, Alexander	Exploring Natural Sceneries: A Comparative Eye-Tracking Study of Freely Moving Participants in Virtual and Real Environments
1-37	Kus, Oliwia	The role of vocabulary size and contextual diversity on word learning during sentence reading
1-38	Ziaka, Laoura	Return Sweeps in Serial Naming Tasks: What does matter

Wednesday afternoon - Poster Session 2		
2-1	Attar, Eyad	Investigating Brain Seizure Activity Detection in Epilepsy: Insights from EEG Analysis of Seven Individuals
2-2	Li, Alice R. P.	Implementing Automatic Tagging to Address Dynamic Areas of Interest: A Human-Computer Vision Perspective
2-3	Barbara, Nathaniel	EOG-Based Ocular Angle Estimation Without Assuming Equal Vertical Ocular Angles
2-4	Liang, Feifei	Sub-lexical semantic decoding in incidental word learning during natural Chinese reading
2-5	Limachya, Rupali	Using Fixated-Related Potentials to Investigate Prediction Error during Natural Reading
2-6	Lin, Ting-Yi	The Influence of Mechanistic Plausibility on Reading Time for Mechanistic Information and Covariation Data in Scientific Texts
2-7	Liu, Nina	Unpacking the relation between morphological awareness and word processing during sentence reading in Chinese children
2-8	Malevich, Tatiana	Effects of spatially congruent and incongruent sounds on visually-driven microsaccade direction modulations during primary visual cortex inactivation

Board	Name	Title
2-9	Mézière, Diane C.	Eye Movement Indicators of Mind-Wandering during Reading: A Meta-Analysis
2-10	Nawabutsitthirat, Wiralpach	How reading on a computer affects comprehension in college-aged readers: An eye movement investigation
2-11	Niehorster, Diederick C.	gazeMapper: A tool for automated world-based analysis of wearable eye tracker data
2-12	Ovsepian, Rozana	Target size modulates smooth pursuit gain in patients with schizophrenia spectrum disorder
2-13	Pandey, Dr. Aparna	Eye movements in biliterate children with and without dyslexia in reading English and Kannada
2-14	Pingpank, Thore	Can Local Meaning Predict Task-Dependent Fixation Patterns?
2-15	Polgári, Patrik	An interplay between forward and backward saccade adaptation
2-16	Popelka, Stanislav	Advancing dyslexia intervention with gaze-based interactions in DeveLex software
2-17	Potthoff, Jonas	Cookie cravings – Sugar content information affects Christmas treat preferences
2-18	Ptukha, Anna	Eye movements as a potential mechanism for synchrony perception plasticity
2-19	Rajora, Ananya	An analysis of eye movements of novice and expert Wordle players

Board	Name	Title
2-20	Reintanz, Lisa	Experimental design of eye tracking based validation of customer requirements
2-21	Ryseva, Kseniia	Dynamics of Eye Movements during Schulte Tables Completion in Stressful Situation
2-22	Höfler, Margit	How much time is left? Effects of time pressure in visual search
2-23	Schwalm, Laura	Speeding through the lines: Effects of reading speed on eye movement control and word processing
2-24	Seernani, Divya Prakash	Webcam based eye-tracking -Validation Study Report
2-25	Staroverova, Vladislava	Perceptual span during reading in Russian
2-26	Tsai, Jie-Li	The influence of the unselected meaning of homographs in reading Chinese sentences
2-27	Vetter, Celina	Enhancing Human Performance in Air Traffic Control using Eye Tracking Technology and Artificial Intelligence Support
2-28	Virtanen, Oskari J.	The WHO said what? Interaction of source credibility and readers’ prior beliefs in the reading of social media posts
2-29	Vojtechovska, Michaela	Unveiling Religious Imagination Through AI and Eye-Tracking
2-30	Vorstius, Christian	Eye-movement patterns of subclinical body dysmorphic individuals in social situations

Board	Name	Title
2-31	Walter, Kendall E	Characterizing the Variability of Eye Movement Behaviours in Children Reading English: A Corpus Study
2-32	Kocdemir, Gamzee	An Eye-Tracking Study to Understand the Connections Between Social Rank Information, Task Performance, Sleep and Mental Health
2-33	Wu, Yushu	Is There a Preferred Viewing Locations during Chinese Reading? Novel evidence from Hong Kong Corpus (of Chinese Sentence and Passage Reading)
2-34	Yip, Michael C. W.	Processing Code-Switched Words Interactively: An Eye-tracking Study
2-35	Zang, Chuanli	Parafoveal processing of Chinese four-character idioms with symmetrical and asymmetrical structure
2-36	Zhou, Li	Attentional Disengagement Differences in Young Children with Autism: A Comparative Eye-movement Study Using Static and Dynamic Stimuli
2-37	Zhu, Jiahui	Diagnosis of Schizophrenia by Integrated Saccade Scores
2-38	Bertram, Raymond	Effects of Decoding and Linguistic Skills on Reading Fluency and Comprehension in Finnish 2nd and 3rd Graders

Abstracts

22nd European Conference on Eye Movements

August 25th - 29th, 2024

Keynotes

Monday, August 26th, 09.00 - 10.00

Venue A

Gaze and Eye Movement as Input for Human-Computer Interaction

Hans Gellersen
Lancaster University, UK

Where we look not only reflects our information needs, but also guides our movements and actions in the world. As gaze is so central to our interactions, it has been studied as an input modality for human-computer interaction (HCI) for as long as we have had modern computer interfaces. However, in decades of research, the approach has been to isolate eye movement as input, and to treat it as separate from other movements of the body that we use for interaction with computers. In this talk I will highlight how closely the movement of our eyes is coupled with other movement, of objects in the visual field, and the movement of our hands, head and body, and discuss examples of novel interfaces that leverage eye movement in concert with other motion and modalities.

Tuesday, August 27sth 9.00 - 10.00

Venue A

Transsaccadic integration of peripheral and foveal feature information

Alexander Schütz
University of Marburg, Germany

Visual processing is not homogeneous across the visual field: acuity and contrast sensitivity peak at the fovea in the center of the visual field. The peripheral visual field allows for a large field of view at lower resolution. Despite these differences, humans typically do not notice changes in visual information that result from bringing an object from the periphery to the fovea with a saccadic eye movement. I will present a series of studies demonstrating where, when and how pre- and postsaccadic feature information can be integrated in a manner close to statistically optimal. The detection of feature changes is impaired across saccades but shows that appearance is in general well calibrated between peripheral and foveal vision. These findings support the view that peripheral and foveal vision are interrelated despite their functional specialization.

Wednesday, August 28th, 9.00 - 10.00

Venue A

Canyoureadthis? The critical but overlooked role of word-segmentation during reading

Xinshang Li
Chinese Academy of Sciences, Beijing

Given that words serve as the fundamental processing units in all languages, the initial stage of reading involves the parsing of natural text into discrete units of text, or word segmentation. Without proper word segmentation, readers cannot comprehend text accurately. While this process may appear straightforward in languages that employ explicit cues to demarcate words (e.g., inter-word spaces), it poses considerable challenges for languages where words lack explicit demarcation, as seen in Chinese. Unfortunately, this crucial aspect of reading has historically been overlooked. Traditional reading models, which were usually designed for spaced languages, word segmentation is not an issue at all because readers can easily do so with the aid of inter-word spaces. Nevertheless, these models encounter substantial difficulties when applied to unspaced languages. In this talk, I will report recent experimental findings on the cognitive mechanisms involved in word segmentation and eye-movement control during Chinese reading. Then I will introduce a computational model, called the Chinese reading model (CRM), which was designed to simulate reading processes during Chinese reading. Furthermore, I will report some eye-tracking studies that investigate how word segmentation interacts with higher-level language processing, including syntax, semantics, and pragmatics. Finally, I will discuss how research on languages using different word demarcation methods have informed our insights into universal and script-specific reading mechanisms.

.

Thursday, August 29th, 9.00 - 10.00

Venue A

Allocating attention for information gain: the roles of uncertainty, information diagnosticity and cognitive costs

Jacqueline Gottlieb
Columbia University, New York

Attention has long been an unavoidable and highly controversial construct in cognitive science. Much of the controversy surrounding this topic, I argue, stems from a lack of theoretical frameworks describing “top-down” attention control – the mechanisms that align attention with behavioral goals. I will describe a novel theory proposing that attention – and, in monkeys and humans, saccades – are deployed to maximize expected information gains (EIG) – and a neurocomputational model of EIG-based saccadic control in the fronto-parietal and executive networks. I will discuss evidence supporting the model and some of its implications for mechanisms of uncertainty and EIG estimation in different contexts and tasks.

Talks

Monday - morning - Symposium 1: Crosslinguistic Explorations - Venue A

Presenting Wave 2 of the MulAlingual Eye Movement Corpus (MECO)

Victor Kuperman[1] & Noam Siegelman[2]
[1]McMaster University, Canada, [2]Hebrew University of Jerusalem, Israel
vickup@mcmaster.ca

Recent multilingual collections of eye movements during text reading have boosted cross-linguistic, behavioral and computational reading research. This talk presents Wave 2 of the Multilingual Eye-Movement Corpus (MECO), an expansion that adds eye movement records from 8 new written languages to the 13 languages covered in Wave 1 of MECO, including traditional and simplified Chinese and Hindi. First, we will validate hypotheses about universal oculomotor behavior based on the (largely alphabetic) languages of Wave 1 against a greater number and diversity of writing systems. This discussion touches upon the lexical benchmark effects on eye movements and the correlation between preferred landing positions and language-specific word length distributions. Second, we present new findings on the interaction between oculomotor control and the writing systems, with a focus on the well-established wrap-up effect. Finally, the combined two waves of MECO contain multiple eye movement samples from the same language (English, German, Hindi, Russian, Spanish). This "redundancy" helps determine whether each written language has a behavioral footprint distinct from other languages. The overarching question underlying this research asks which features of eye movement behavior during reading are universal and which are contingent on the structure and conventions of the specific written language and system.

Universality in eye movements and reading: A replication with increased power

Jukka Hyönä[1], Simon P. Liversedge[2], Henri Olkoniemi[3], Chuanli Zang[2,4,] Xin Li4, Guoli Yan[4], Xuejun Bai[4], Zhichao Zhang[4,1]
University of Turku, Finland, [2]University of Central Lancashire, UK, [3]University of Oulu, Finland, [4]Tianjin Normal University, China
hyona@utu.fi

We present findings from a large scale replicaAon of Liversedge, Drieghe, Li, Yan, Bai and Hyönä (2016) that invesAgated reading in Chinese, Finnish and English. The original results showed robust differences in fine grained characterisAcs of eye movements between languages whilst overall sentence reading Ames did not differ and were interpreted to reflect universality in aspects of processing in reading. The study was criAcized as being underpowered (Brysbaert, 2019), did not follow current best staAsAcal pracAce (e.g., no random slopes in LMMs), nor did it assess effects across all three languages simultaneously. Therefore, we tested 80 new subjects in each language and undertook beFer analyses. The new analyses showed shorter total sentence reading Ames for Chinese relaAve to Finnish and English readers (probably due to Chinese cultural changes). Other findings reported in the original study were consistent, and again, reflect universality in aspects of reading. We are currently invesAgaAng processing of more fine-grained, comparable, linguisAc units across languages and we will also discuss these results in the talk. And we will briefly comment on the criAcisms regarding the lack of staAsAcal power levelled against the original arAcle.

What is universal in eye movement control during reading? Landing Positions of Regressive and Progressive Saccades in 13 languages

Laura Schwalm, Ralph Radach
University of Wuppertal, Germany
lschwalm@uni-wuppertal.de

We examined how eye movement control during reading can be adjusted to changes in reading speed. Five-sentence paragraphs were presented in grey font, with only one line highlighted in black from top to bottom at a predefined speed. This method allows for good experimental control of speed while preserving nearly natural reading.
As a baseline, we determined the individual reading speed of forty participants. The Line-by-Line technique was applied at 100% of the baseline speed, followed by 125% and then 150%. Materials consisted of 72 items, each containing five sentences (279 – 423 words).
Results indicate that at higher speeds fewer words were fixated. However, there was no change in first-pass reading times, as faster word reading was mainly achieved through a reduction in regressions and re-reading. The word frequency effect persisted across all speeds, with a smaller difference between high and low-frequency target words in total viewing time at higher reading speeds.
These results suggest that experienced readers have a substantial residual capacity that can be utilized when reading speed increases, allowing for maintained word processing and comprehension. At higher speeds the rate of re-reading is reduced, indicating that a more relaxed criterion for comprehension (monitoring) is adopted..

Eye movements of children and adults reading in three different orthographies: The effects of the past, present, and future words

Tuomo Häikiö[1], Jukka Hyönä[1], Simon P. Liversedge[2], Ascensión Pagán[3], Sascha Schroeder[4]
[1]University of Turku, Finland; [2]University of Central Lancashire, UK; [3]University of Leicester, UK
[4]University of Goettingen, Germany
tuilha@utu.fi

We examined the developmental aspects of eye movements during reading in three languages, namely English, German, and Finnish. These European languages vary in their orthographic complexity and predictability, with the orthography being deeper in English and German than in Finnish, but the predictability being simple in German and Finnish while English has many exceptions. Fourth-grade children and adults read sentences that had been carefully translated and back-translated across all three languages. Each sentence contained a target word with frequency and length manipulation. In the present study, we analyzed all of the words in the target and filler sentences apart from some exceptions, such as first and last words of the sentences. In the analyses, we included several predictors, namely frequency and word length of the words N-1, N, and N+1 while controlling for whether the previous word had been refixated. Apart from the usual length and frequency effects for all reader groups, there were language differences. Most notably, both German children and adults showed consistent parafoveal frequency effects. In addition, English children and adults showed less parafoveal processing when encountered with a longer word. The results will be discussed from the viewpoint of complexity and predictability of the language systems.

Eye Movements of adults reading three different orthographies as a second language

Sascha Schroeder[1], Tuomo Häikiö[2], Ascensión Pagan[3], Jukka Hyönä2, & Simon P. Liversedge[4]
[1]University of Goevngen, Germany, [2]University of Turku, Finland, [3]University of Leicester, UK, [4]University of Central Lancashire, UK
sascha.schroeder@psych.uni-goevngen.de

Eye-tracking studies have shown that adults who are reading texts in a second language (L2) read more slowly and make more and longer fixaAons than adults who are reading the same texts in their nave language (L1). This behavior is similar to that of children who are reading in their L1. However, no study has directly compared adults' L2 eye-movements with that of children reading the same materials yet. In the present study, we invesAgated three groups of bilingual adults who had learned English, Finnish, or German as a second language. Each group read single sentences that had been carefully translated into all three languages. In addiAon, we are able to compare their data with that from skilled adults and 4th grade children who read the same sentences in their L1. Results show that adults' reading behavior in their L2 read the sentences slower, made more fixaAons, and had lower skipping but higher refixaAon and regression rates. Axer controlling for interindividual differences in language proficiency, eye movements of children reading in their L1 and adults reading in their L2 were nearly indisAnguishable. We will discuss our findings with regard to current models of reading development and second language processing.

Individual Differences in Word Skipping During Reading in English as L2

Denis Drieghe[1], Diana Esteve[2], Bernhard Angele[3], Victor Kuperman[4] & Manuel Perea[3,5]
[1] University of Southampton, UK, [2] University of Cambridge, UK, [3] Universidad Nebrija, Spain, [4]McMaster University, Canada, [5]Universitat de València, Spain
d.drieghe@soton.ac.uk

The Multilingual Eye-movement Corpus (MECO; Siegelman et al., 2022) contains data from unbalanced bilinguals reading in their first language (L1) for a variety of languages and in English as their second language (L2). We analysed word skipping in L2 on the basis of five predictors consisting of the frequency and length of the word in L2 and three measures of individual differences. Besides L2 proficiency, two novel measures were also constructed: the average amount of skipping in L1 across participants per language and whether an individual reader skips words in L1 compared to their peers. Word skipping in L2 increased for short and high-frequency words, for partipants with higher L2 proficiency, for readers whose L1 featured relaAvely high average skipping rates compared to the other languages, and especially for parAcipants who skip more oxen in L1 than their peers. Our results show that readers prefer to maintain a certain level of word skipping resembling how they read in L1. Due to lower L2 than L1 proficiency in unbalanced bilinguals, word skipping in L2 would oxen be based on a comparatively less advanced stage in parafoveal word recognition. Additional analyses including predictability as a predictor will also be presented.

Monday - morning : Visual Perception and Saccade Control- Venue B

When Time Disrupts: Interruptions in Visual Searches

Alejandro Javier Cambronero Delgadillo[1], Christof Körner[1], Iain Gilchrist[2], Margit Höfler[1]
[1]Department of Psychology, University of Graz, Austria; [2]School of Experimental Psychology, University of Bristol, United Kingdom
a.cambronero-delgadillo@uni-graz.at

Interruptions in visual search are a common occurrence in daily life; however, they have not been sufficiently explored in research. Our work investigates this topic by focusing on time pressure as a potential disruptor in visual search tasks. We conducted three experiments where participants were asked to locate a T-shaped target among L-shaped distractors. Each trial featured two consecutive, distinct displays, each containing one target. In the first experiment, we assessed the impact of various levels of time pressure on search performance. In the second experiment, we introduced a reward condition, placing a higher value on identifying the target in the second display. In the third experiment, we introduced a condition in which the number of items in the second display was reduced, making the second search presumably easier. Overall, our results revealed that search accuracy significantly decreased as time pressure increased. Moreover, under time pressure, participants often prioritized completing the first search over a potentially more rewarding or easier second search, opting to undertake the second search only when time allowed. These findings suggest that time pressure can significantly hinder the ability to optimally modify strategies according to the demands of the search task.

Influence of Saccades on Cortical Modularity During a Feature Discrimination Task

George Tomou[1], Bianca Baltaretu[2], Amirhossein Ghaderi[1], John Douglas Crawford[1]
[1]York University, Canada; [2]University of Giessen
jdc@yorku.ca

Visual cortex shows both dorsoventral and hemispheric modularity, but it is not clear if/how these modules persist, disperse and communicate when saccades require increased sharing of spatial information. Here, we address this issue using fMRI data measured while 17 human participants discriminated whether an object's shape or orientation changed, with or without an intervening saccade across the object. A region of interest analysis confirmed transsaccadic orientation modulations in right parietal cortex and shape modulations in dorsomedial occipital cortex (Balaretu et al. Sci. Reports 2023). We then applied graph theory analysis to BOLD time courses from 50 vision-related cortical nodes identify local and global network properties. Modularity analysis revealed three sub-networks during fixation: a bilateral 'visuospatial' module in parietofrontal cortex and two lateralized occipitotemporal networks linking areas implicated in object feature processing. When horizontal saccades required comparisons between visual hemifields, information transfer increased, and the two lateralized ventral modules became functionally integrated into a single bilateral sub-network. This network included 'between module' connectivity hubs in lateral intraparietal cortex and dorsomedial occipital areas implicated in transsaccadic integration. These results provide objective support for functional modularity in the visual system and show that the hemispheric sub-networks are modified and functionally integrated during saccades.

Saccades, blinks and pupil responses in youth with an eating disorder: Findings from a multi-site Canadian study using an interleaved pro-saccade/anti-saccade task

Ryan H Kirkpatrick[1,2], Linda Booij[3,4], Heidi C Riek[1], Jeff Huang[1], Isabell C Pitigoi[1], Donald C Brien[1], Brian C Coe[1], Jennifer Couturier[5], Sarosh Khalid-Khan[6], Douglas P Munoz[1,2]
[1]Centre for Neuroscience Studies, Queen's University, Kingston, Ontario, Canada; [2]Department of Medicine, Queen's University, Kingston, Ontario, Canada; [3]Eating Disorders Continuum, Douglas Mental Health University Institute, Montreal, Quebec, Canada; [4]Department of Psychiatry, McGill University, Montreal, Quebec, Canada; [5]Department of Psychiatry and Behavioural Neurosciences, McMaster University, Hamilton, Ontario, Canada; [6]Department of Psychiatry, Queen's University, Kingston, Ontario, Canada
12rhkl@queensu.ca

Early treatment for eating disorders (EDs) increases recovery likelihood. The identification of biomarkers may improve our understanding of EDs and guide early detection and treatment planning. This study used eye tracking to explore possible biomarkers in females with EDs using an interleaved pro-saccade and anti-saccade task. Individuals with an ED (n=65, Mage=17.16±3.5 years) were compared to controls (HC; n=65, Mage=17.88±4.3 years). The ED group was further divided into two ED phenotypes: individuals with a restrictive spectrum ED (ED-R; n=43) or a bulimic spectrum ED (ED-P; n=22). In pro-saccade trials, HCs made significantly less fixation breaks than ED ($F(1,128)=5.33$, $p=0.023$). The ED-P group made the most anticipatory pro-saccades, followed by ED-R then HCs ($F(2,127)=3.38$, $p=0.037$). HCs had the highest proportion of correct pro-saccades, followed by ED-R, then ED-P ($F(2,127)=4.93$, $p=0.009$). The ED group had a significantly lower baseline pupil size ($F(2,127)=3.60$, $p=0.030$) and lower pro-saccade dilation velocity ($F(2,127)=3.30$, $p=0.040$). HCs had the highest blink probability during the intertrial interval (ITI), followed by ED-P with ED-R having the lowest ITI blink probability ($F(2,125)=3.63$, $p=0.029$). These results suggest that eye tracking may identify objective biomarkers of ED in youth. This may assist clinicians to establish an ED diagnosis and treatment plan more quickly.

Sequences of eye movements: the role of the Gestalt

Eugene McSorley
University of Reading, United Kingdom
e.mcsorley@reading.ac.uk

We execute multiple saccades every day but a lot of basic research uses tasks that only require single responses. When sequences of saccades are required they are reported to be programmed in parallel: Saccade latencies reduce, and saccade metrics are affected by future saccade target locations. These effects extend over quite a large range future target locations. We have suggested that this pattern may, to some extent, be due to eye movement responses being driven by the coarse scale perceptual organization of the target sequence into a larger Gestalt path. This Gestalt guidance signal acts against the accurate localization of the individual path elements. To examine this, we manipulated the grouping of saccade target elements (by colour or connecting lines) in a task where participants were asked to saccade to each of the individual target spots without reference to groupings. Despite being immaterial to task completion, saccade accuracy was worse and saccade latencies were quicker as grouping increased. This shows that the Gestalt of the target pattern was extracted quickly and used as a guidance signal in control of saccades.

Eye movements reflect improvements in search for heterogeneous categories

Arryn Robbins
University of Richmond, United States of America
arobbins@richmond.edu

Subprocesses of categorical search, like attentional guidance and target verification, are shaped by featural regularities, including category-consistent features and those appearing across multiple trials. We investigated how category heterogeneity impacts the learning of these featural regularities. In two experiments, participants classified novel categories (rocks) during a training task, and later searched for them with an Eyelink 1000 recording eye movements. Using multidimensional scaling, we measured each category's level of heterogeneity. We found that heterogeneous rock categories, marked by inconsistent features, showed poorer classification performance during training and testing. However, continuous learning was evident throughout search trials, as shown by improvements in response time and eye tracking measures of target verification. In contrast, homogeneous categories exhibited minimal improvements, indicating a ceiling effect in learning potential. No changes in eye movements associated with attentional guidance were observed across trials, regardless of level of heterogeneity. These findings suggest that the ongoing featural learning during categorical search is not equal across categories nor sub processes of search. The results have implications for training of heterogeneous categories, which may facilitate ongoing learning, unlike homogeneous categories, where search improvements are less pronounced.

Unexpected sounds induce a rapid inhibition of eye-movement responses

Martin R. Vasilev[1], Zeynep G. Ozkan[2], Julie A. Kirkby[3], Antje Nuthmann[4], Fabrice B. R. Parmentier[5,6,7]
[1]University College London, United Kingdom; [2]University of Valencia, Spain; [3]Bournemouth University, UK; [4]Kiel University, Germany; [5]University of the Balearic Islands, Palma, Spain; [6]Balearic Islands Health Research Institute, Palma, Spain; [7]University of Western Australia, Australia;
m.vasilev@ucl.ac.uk

Unexpected sounds have been shown to trigger a global and transient inhibition of motor responses. Recent evidence suggests that the movement of the eyes may also be inhibited in a similar way, but it is not clear how quickly unexpected sounds can affect eye movement responses. Additionally, little is known about whether unexpected sounds affect only voluntarily- generated saccades or also saccades that are more reflexively generated. In this study, participants performed a pro-saccade and an anti-saccade task while the timing of sounds relative to stimulus onset was manipulated. Unexpected novel sounds inhibited the execution of both pro- and anti-saccades compared to standard sounds, but the inhibition was stronger for anti-saccades. The timeline of the novelty distraction effect was relatively stable– it was observed from 150 ms before the target onset to 25 ms after the target onset. Interestingly, unexpected sounds also reduced anti-saccade task errors, indicating that they aided inhibitory control. Overall, these results suggest that unexpected sounds yield a global and rapid inhibition of eye-movement responses. This inhibition also helps suppress reflexive eye-movement responses in favour of more voluntarily-generated ones.

Monday - morning - Symposium 2: Understanding Interaction - Venue C

Exploring the dynamics of ambient and focal visual attention of novices in continuous construction tasks. A heavy-machinery simulator and eye-tracking study

Baldisserotto, Filippo
Institute of Psychology SWPS University Warsaw, Poland
fbaldisserotto@swps.edu.pl

The distinction between the two main modalities of visual attention dynamics is a known theme in the literature. These dynamics describe modes of scene exploration (ambient) and local exploration (focal). The results from studies using still images show a consistent difference between saccadic length and fixation duration across the length of the task. Specifically, the first phases of image perception are characterized by longer saccades and shorted fixations (ambient) while later stages are characterized by more focal eye movement behavior with short saccades and longer fixation duration. Similar patterns are present when the participants are exposed to videos in which the scene suddenly changes after a cut. The present study aims to explore the visual attention dynamics of novices while operating a heavy-machine simulator, simulating load-unload and digging-unload tasks using eye-tracking. The attention dynamics of the visual task are analyzed, to discern ambient and focal phases during a continuous task, that are related to the task's characteristics. It is expected that the task's characteristics influence the ambient and focal dynamics of the task therefore each task has been divided into subtasks. A more focal visual behavior is expected when the operator is loading the material on the truck or during the excavating phase the visual behavior, as longer fixations are expected in areas where the main center of interest is located. When the specific sub-task is finished, on the contrary, it is expected a more ambient visual behavior in the first seconds when a new part of the task begins that progressively becomes more focal as the subtask progresses.

Gaze Interaction in XR

Duchowski, Andrew Ted
Clemson University (Clemson, SC, USA)
duchowski@clemson.edu

The talk starts with an overview of XR applications, featuring eye-tracking interaction, in virtual reality, games, and other venues. The focus is on five forms of applications: diagnostic (off-line measurement), active (selection, look to shoot), passive (foveated rendering, a.k.a. gaze-contingent displays), assistive (translation), and expressive (gaze synthesis).

Predictive Model of Intrinsic Cognitive Load from Physiological Measures

Ekin, Merve
Institute of Psychology SWPS University Warsaw, Poland
mekin@swps.edu.pl

Intrinsic cognitive load is associated with the inherited complexity and the number of the elements interacting in a mental task. The aim of this study was to predict the level of intrinsic cognitive load in mental calculations using eye movement and biometric parameters. Cognitive load was assessed using several physiological measures such as the K-coefficient (fixation duration- saccadic amplitude), pupil dilation, heart rate, and galvanic skin response. Thirty-four participants (aged 21.18 ± 3.42) performed mental calculations while their physiological data were recorded by the Gazepoint GP3 HD eye tracker with psychophysiology bundle. We also controlled for working memory with a digit span task. As expected, physiological measures changed according to the level of task difficulty. For example, changes in pupil activity were greatest for hard (requiring borrowing or carrying steps during addition or subtraction operations) and long (two-digit) calculations. The results lead to a reliable predictive model of intrinsic cognitive load based on psychophysiological metrics with high discriminative power. The model can be applied to future real-time predictions based on machine learning algorithms in educational settings.

Gaze-Led Audio Description. Eye-movement data driven technology for cultural inclusion

Krejtz, Izabela
Institute of Psychology SWPS University Warsaw, Poland
ikrejtz@swps.edu.pl

This talk presents the concept of Gaze-Led Audio Description (GLAD) and its application for the interactive system enhancing accessibility in urban environments. While Audio Description (AD) typically relies on the expertise of individuals with deep knowledge in the subject matter, such as art historians or architects, studies indicate that their perceptual biases may inadvertently create barriers for non-experts. For instance, descriptions of architectural heritage often follow a structured approach based on expert analysis, which may not align with the natural viewing patterns of lay observers. We argue that AD could benefit from aligning with the visual attention patterns of non-experts, thereby improving accessibility. Drawing from eye tracking experiments, GLAD represents a novel data-driven approach to accessibility technologies providing insights into the potential adjustments needed to optimize AD for broader audience comprehension and enhances accessibility.

Comparing Visual Attention Dynamics of Tram Drivers: Expertise Impact on Safety Awareness

Anna Warchoł-Jakubowsk
Institute of Psychology, SWPS University, Warsaw, Poland
awarchol-jakubowska@swps.edu.pl

Two eye-tracking studies compared visual attention among tram drivers of differing expertise levels. In the initial study, 23 experts and 24 novices viewed tram-driving simulations videos, focusing on windshields and control panels. Results revealed between-group differences in attention dynamics, with novices focusing more on the tram simulator's middle panel for speed control compared to experts. Experts demonstrated more focal attention and maintained their concentration for a longer time than novice tram drivers. This disparity was particularly notable in critical areas such as windshield views. In the second study, 25 experts and 20 novices navigated a simulated route featuring tram stop operations, track intrusions, and turns. Experienced drivers maintained a higher level of focused attention compared to novices, especially during crucial moments. Analysis indicated that expert tram drivers' ability to switch between focused and ambient attention aligned more closely with operational demands for safety. These findings will inform the development of visual attention training programs for novices.

Monday - afternoon - Visual Processing and Eye Movement Control in Reading - Venue A

Comparative Analysis of Saccadic Main Sequences in Horizontal and Vertical Reading

Yaqian Bao, Victor Kuperman
McMaster University, Canada
baoy47@mcmaster.ca

It is well-known that there is a consistent relationship between the duration, peak velocity, and amplitude of saccades, known as the ‘main sequence’. Specifically, as the amplitude of saccades increases, so do their duration and peak velocity (Bahill et al., 1975). Our study focuses on how the direction of reading influences this main sequence. Prior research on horizontal-vertical anisotropy (HVA) suggests that human visual processing is more efficient along the horizontal mid-line compared to the vertical mid-line at a given eccentricity (Rovamo & Virsu, 1979). HVA predicts differences between reading texts written vertically versus horizontally. We utilized the Multilingual Eye-Movements Corpus (MECO, Siegelman et al., 2022) to compare saccadic movements in vertical traditional Mongolian script with 13 horizontally written languages. The analysis confirmed that the systematic main sequence relationship is universal across multiple written languages and systems. A novel finding is that saccades of the same duration exhibit consistently shorter amplitudes in vertical reading, particularly in traditional Mongolian compared to horizontally written languages ($F_{1,626} = 117.5$, $p < .001$). This finding aligns with HVA predictions, and we discuss it from the perspectives of eye anatomy and visual attention.

Bayesian inference of the simplified SWIFT model of eye-movement control in reading

Ralf Engbert
University of Potsdam, Germany
ralf.engbert@uni-potsdam.de

Dynamical models are crucial for developing process-oriented, quantitative theories in cognition and behavior. Due to the impressive progress in cognitive theory, domain-specific dynamical models are complex, which typically creates challenges in statistical inference. Mathematical models of eye-movement control might be looked upon as a representative case study. In this tutorial, we introduce and analyze the SWIFT model (Engbert, Longtin, & Kliegl, 2002; Engbert, Nuthmann, Richter, & Kliegl, 2005), a dynamical modeling frame- work for eye-movement control in reading that was developed to explain all types of saccades observed in experiments from an activation-based approach. We provide an introduction to dynamical modeling, which explains the basic concepts of SWIFT and its statistical inference. We discuss the likelihood function of a simplified version of the SWIFT model as a key foundation for Bayesian parameter estimation (Rabe et al., 2021; Seelig et al., 2020). In posterior predictive checks, we demonstrate that the simplified model can re- produce interindividual differences via parameter variation. All computations in this tutorial are implemented in the R-Language for Statistical Computing and are made publicly available. We expect that the tutorial might be helpful for advancing dynamical models in other areas of cognitive science.

Unpacking Word Segmentation Processes by L2 Chinese Readers: Evidence from Eye Movements

Lin LI[1], Sha LI[2], Jingyi LIU[1], Shan WANG[2], Kevin B. Paterson[3]
[1]Tianjin Normal University, China, People's Republic of; [2]Fujian Normal University, China, People's Republic of; [3]University of Leicester, UK;
linpsy@outlook.com

Sentences in written Chinese are composed of continuous sequences of characters, without spaces or other visual cues to mark word boundaries. While skilled L1 readers can efficiently segment this naturally-unspaced text into words, little is known about the word segmentation capabilities of L2 readers, including whether they employ the same strategies to process temporary segmental ambiguities. Accordingly, we report two eye-movement experiments that investigated the processing of sentences containing temporarily ambiguous “incremental” three-character words whose first two characters can also form a word, comparing the performance of 48 L1 Chinese readers and 48 high-proficiency L2 Chinese readers in each experiment. In Experiment 1, either the three-character word or its embedded word was presented as the target word in sentence contexts where the three-character word always was plausible, and the embedded word either plausible or implausible. Both groups produced similar plausibility effects, consistent with readers accessing the embedded words early during ambiguity processing before ultimately assigning an incremental word analysis. Experiment 2 provided further evidence that readers can access the embedded word early during ambiguity processing but with both groups rapidly selecting its incremental word analysis. We discuss our findings in relation to models of eye movement control in Chinese reading.

Cross-linguistic comparison in reading sentences of uniform length: Visual-perceptual demands override readers' experience

Olga Parshina[1], Nina Zdorova[2,3], Victor Kuperman[4]
[1]Middlebury College; [2]HSE University; [3]Institute of Linguistics Russian Academy of Sciences; [4]McMaster University
oparshina@middlebury.edu

This study presents findings of a cross-linguistic comparative experiment that tests predictions of two accounts of eye-movement spatial control in reading. The *dynamic adjustment account* posits that readers plan saccade lengths based on immediate input characteristics and rapidly adjust saccade length to meet input demands. Conversely, the *discrete control account* argues that readers develop preferences for saccade lengths based on their experience with the language. The design of this study follows Cutter et al.'s (2017) study design in English: Russian speakers (N=24) read 4 sets of sentences, each set consisting of sentences composed of words of uniform length. Given the differences in word length distributions between two languages, these accounts yield differential predictions. Per the dynamic adjustment account, the saccades of Russian and English readers would be of the same length, resulting in similar landing positions. If the readers' experience matters as per discrete control account, Russian readers would produce longer saccades and farther landing positions. The results corroborated the former account: Russian readers showed rapid and accurate adjustment of saccade lengths and landing positions to the input constraints. Crucially, the saccade lengths and landing positions did not differ between English and Russian readers even in the cross-linguistically length-matched stimuli.

Perceptual and word identification spans do not differentially impact when and where saccade decisions

Elizabeth R Schotter[1], Casey Stringer[1], Emily Saunders[2], Frances G Cooley[3], Grace Sinclair[1], Karen Emmorey[2]
[1]University of South Florida, USA; [2]San Diego State University, USA; [3]Rochester Institute of Technology, USA; eschotter@usf.edu

Theories of reading posit that "where" and "when" saccade decisions are driven by visual and linguistic factors, extracted from the perceptual span and word identification span, respectively. We tested this hypothesized dissociation by masking, outside of a visible window, either the spaces between the words (to assess the perceptual span) or the letters within the words (to assess the word identification span). Analysis of reading rate (overall reading efficiency), suggested the perceptual span extended to between 10-14 characters and the word identification span extended to 8 characters to the right of fixation, but the spans did not dissociate between where decisions (saccade length) or when decisions (fixation duration). We also tested deaf readers because they have documented larger spans; however, neither dissociated span was larger than that of the hearing readers, suggesting their enhanced span requires integration of both types of information. They did show a larger increase in reading rate when leftward text was available, suggesting a more symmetrical distribution of attention. Our results challenge the assumed dissociation between type of reading span and type of saccade decision, and indicate that reading efficiency requires access to both perceptual and linguistic information in the periphery, and bilaterally for deaf readers.

Accounting for Chinese readers' eye-movement behavior using universal visuo-motor computations in the midbrain: Evidence against a role of word segmentation

Françoise Vitu[1], Hossein Adeli[2], Gregory J. Zelinsky[3,4]
[1]Aix-Marseille Université, CNRS, France; [2]Columbia University, Zuckerman Institute, USA; [3]Stony Brook University, Department of Psychology, USA; [4]Stony Brook University, Department of Computer Science, USA
Francoise.Vitu-Thibault@univ-amu.fr

Models of eye-movement control during reading classically assume that word segmentation, enabled by fast extraction of inter-word spaces in alphabetic languages, is a necessary process by which readers select the next word to be fixated. However, this suggests that Asian unspaced scripts are read using less-efficient and/or different saccade-targeting strategies. Here we show using MASC, our Model of Attention in the Superior Colliculus (SC), that word segmentation is unnecessary, and inter-word spacing superfluous, for eye-movement guidance. MASC predicts sentence-reading scanpaths by first computing the distribution of luminance contrast over the retina-transformed sentence image and then averaging this visual-saliency map over visual and motor neuronal populations in SC space. It was previously shown to generate prototypical word-based eye-movement behavior over French sentences. In the present study, MASC was presented with 120 unspaced sentences from the Beijing Sentence Corpus, and its behavior was compared to that observed in 60 Chinese reading the sentences for comprehension (Pan et al., 2021). MASC nearly perfectly reproduced Chinese readers' forward eye-movement patterns, including their tendency to fixate preferentially the words' first character rather than towards the words' center (as in Westerners). This suggests that illiterate visuo-motor computations underlie readers' eye movements across spaced and unspaced languages.

Monday - afternoon - Neural Basis of Eye Movements - Venue B

Physiological correlates of a simple saccadic-decision task to extended objects in superior colliculus

Baptiste Caziot[1,2], Bonnie Cooper[2], Mark R. Harwood[3], Robert M. McPeek[2]
[1]Marburg University, Germany; [2]SUNY College of Optometry, USA; [3]University of East London, UK
caziot@physik.uni-marburg.de

Saccades are typically studied using small point stimuli, despite the fact that most real-world visual scenes are composed of extended objects. Recent studies in humans have shown that the initiation latency of saccades is strongly dependent on the size of the target (the "size-latency effect"), perhaps reflecting a tradeoff between the cost of making a saccade to a target and the expected information gain that would result. Here, we investigate the neuronal correlates of the size-latency effect in the macaque superior colliculus. We analyzed the latency variations of saccades to different size targets within a stochastic accumulator model framework. The model predicted a steeper increase in activity for smaller targets compared to larger ones. Surprisingly, the model also predicted an increase in saccade initiation threshold for smaller targets. We found that the activity of intermediate-layer SC visuomotor neurons is in close agreement with the model predictions. We also found evidence that these effects may be a consequence of the visual responses of SC neurons to targets of different sizes. These results shed new light on the sources of delay within the saccadic system, a system that we heavily depend upon in the performance of most visuo-motor tasks.

Differences in the control of conjugate and disconjugate components of horizontal fixation drift

Thomas Eggert
LMU University Hospital, LMU Munich, Germany
eggert@lrz.uni-muenchen.de

Fixation is a complex motor behavior involving microsaccades and fixation drift. For frequencies below 80 Hz, the power spectral density (PSD) of the drift position resembles that of a random walk process (~$1/f^2$). Thus, the drift velocity resembles white noise, which is characterized by constant PSD. The fixation drifts of the right and left eye are positively correlated. These properties can be explained by white noise contaminating neural representations of conjugate and monocular components of slow-phase eye velocity (pursuit, vestibulo-ocular reflex, nystagmus). It is not known whether fixation drift is also subject to a specific mechanism for disconjugate control (vergence). To address this question, the PSD of fixation drift velocity was measured binocularly under two conditions. Subjects prepared to perform visual discrimination tasks based on either 1) visual acuity or 2) stereoscopic depth perception. As expected, the version component of the drift velocity resembled white noise. In contrast, the PSD of the vergence velocity increased significantly below 5 Hz. Only the vergence component of fixation drift increased in the stereo task compared to the visual acuity task. The version component was task-independent. These results suggest that the version and vergence components of fixation drift are controlled by different mechanisms.

Sound activates a dormant visual-motor pathway bypassing primary visual cortex

Tatiana Malevich[1,2,3], Matthias P. Baumann[1,2,3], Yue Yu[1,2,3], Tong Zhang[1,2,3], Ziad M. Hafed[1,2,3]
[1]Hertie Institute for Clinical Brain Research, Tuebingen, Germany; [2]Centre for Integrative Neuroscience, Tuebingen, Germany; [3]University of Tuebingen, Tuebingen, Germany
tatiana.malevich@cin.uni-tuebingen.de

Like in other species, the primate visual system features multiple parallel processing streams, besides the geniculostriate pathway. However, the functionality of alternative visual pathways in primates remains unknown; increasing evidence suggests their potential dormancy. We first tested this by reversibly inactivating the primary visual cortex (V1) in two macaque monkeys and investigating a reflexive oculomotor phenomenon, called saccadic inhibition. This reflex, believed to rely on subcortical eye-movement control circuits (Buonocore and Hafed, 2023), is characterized by a short-latency cessation of saccades by visual stimuli, accompanied by saccade direction biasing towards and then away from stimulus location. When we created a localized cortical scotoma, saccadic inhibition was abolished for stimuli in the blind field, confirming the geniculostriate pathway’s dominance. Superior colliculus visual responses were also affected. However, why do alternative visual pathways, including direct retinotectal ones, exist at all? We hypothesized that such pathways might retain functionality, albeit in a gated manner. During V1 inactivation, pairing visual onsets in the scotoma with bilateral sound pulses partially restored saccadic inhibition. Most importantly, it revived saccade direction biasing towards the visual stimulus location, even though the sound was not spatially informative. These results demonstrate that multi-sensory information can activate otherwise dormant visual-motor pathways.

Blinks as a critical oculomotor effector: using eye-tracking and electromyography to understand spontaneous blink behaviour

Isabell C Pitigoi, Brian C Coe, Don C Brien, Douglas P Munoz
Centre for Neuroscience Studies, Queen's University, Kingston, Canada
isabell.pitigoi@queensu.ca

Spontaneous blinks are crucial for lubricating the cornea. However, given the visual interruptions produced by eye closure, they must be strategically timed relative to environmental demands. We previously showed that subjects blink at implicit breakpoints in visual attention during both a structured pro/anti-saccade and unstructured video-viewing task (Pitigoi et al., 2024) and that this behaviour is quickly learned. Here we will present these findings alongside new electromyographic (EMG) data from orbicularis oculi (OO) muscles to demonstrate the accuracy of blink latency and duration measures obtained from Eyelink-1000 video-based eye-trackers. OO-EMG data was collected from 22 healthy participants (11F, ages 18-24yr) performing both voluntary and spontaneous blink tasks. Across subjects, the OO-EMG burst started ~50ms before Eyelink pupil data loss. OO-EMG durations were highly variable between subjects yet correlated strongly to Eyelink pupil data loss durations, which were substantially longer. Understanding these timing considerations will allow for more precise quantification of blink behaviour, which is becoming increasingly important as a supplement to other oculomotor effectors (saccade, pupil) obtained from eye-tracking. These findings are therefore crucial to establish the physiological validity of blink measures as the literature expands in this exciting new direction.

Fusion Sustaining Oscillations: Dependence on Sustained Convergence Level

John Semmlow[1], Tara Alvarez[2]
[1]Rutgers University, United States of America; [2]New Jersey Institute of Technology
semmlow@gmail.com

Introduction: Vergence fixation is highly accurate indicating some form of feedback control. Previous studies have shown that the fusion sustaining component of disparity vergence contains oscillatory behaviour as expected from a feedback system with neural delays. This study extends a previous examination of oscillatory behaviour to three different levels of sustained convergence.
Methods: Approximately three seconds of steady fixation were recorded on 11 subjects at three sustained convergence levels: 4.0, 8.0, and 12.0 deg. convergent. The frequency spectrum of the vergence component of these fixations was determined using the traditional Fourier transform. Peaks in these spectra associated with oscillatory behaviour were identified and examined.
Results: All subjects exhibited oscillatory behaviour with primary frequencies ranging between 0.35 and 0.65 Hz for all convergence levels along with higher frequency oscillations shown to be harmonics of the primary frequencies. The amplitude of these primary oscillations increased with increasing convergence demand.
Conclusion: The slow oscillatory behaviour found in all subjects at all convergence levels supports the supposition that the fusion sustaining component of disparity vergence is mediated by a feedback control. Dysfunction in feedback control may manifest clinically as additional vergence error that is reflected in the frequency spectrum.

A transient signal in foveal superior colliculus neurons for jumpstarting peripheral saccadic orienting

Tong Zhang[1,2], Ziad M. Hafed[1,2]
[1]Werner Reichardt Centre for Integrative Neuroscience, University of Tuebingen; [2]Hertie Institute for Clinical Brain Research, University of Tuebingen
tong.zhang@uni-tuebingen.de

The superior colliculus (SC) is critical for saccade generation. Recent work showed that SC population activity at the time of saccade motor bursts is more temporally aligned than for visual bursts (Jagadisan & Gandhi, 2022). Similarly, population activity in motor bursts resides in different subspaces to visual bursts, and even the sensory signal embedded in SC motor bursts is transformed relative to visual bursts (Baumann et al., 2023). However, how might such a transformation from a visual regime to a motor regime be realized? Here we first show that when a planned saccade is finally released with a go signal, peripheral SC neurons exhibit a robust, short-latency activity pause, before the motor bursts eventually erupt. When we then recorded from foveal SC neurons, we found that these neurons actually burst after the go signal. Remarkably, these foveal bursts occurred (and peaked) several milliseconds earlier than the pauses in the peripheral neurons, and they were not explained by offset responses to the removal of the fixation spot. They were also not sensitive to peripheral target appearance. Thus, we found a transient foveal SC signal jumpstarting peripheral saccadic orienting, likely facilitating a necessary state transition needed for saccade motor bursts to occur.

Monday - afternoon - Oculomotor Measurement Issues - Venue C

Using affordable eye tracking methods to study reading: the role of sampling rate

Bernhard Angele[1], Zeynep Gunes Ozkan[2], Marina Serrano-Carot[1], Jon Andoni Duñabeitia[1]
[1]Universidad Antonio de Nebrija, Spain; [2]Universitat de València, Spain
bangele1@nebrija.es

Eye-movement research has revolutionized our understanding of reading, but the use of eye-tracking techniques is still limited to only a few countries in the world. Publication statistics from the last 25 years show that most publications on eye-movements during reading have authors based in Western countries. We argue that eye-tracking is the ideal technique for reading and language research in countries with limited resources, and that it is crucially important to not just study a small subset of languages, but that more needs to be done to make eye-tracking technology accessible for researchers in those countries. This includes evaluating to what extent cognitive processes during reading can be measured with less expensive eye-tracking devices. One such way may be to use devices with a lower sampling rate, which may be much less expensive than high-sampling rate eye-trackers. We present findings from a study that recorded readers' eye movements during reading at different sampling rates. We show that it is possible to measure the classic effect of word frequency on fixation duration, reflecting ongoing processing during reading, even at sampling rates of 250 Hz and less.

Optimal Bipolar Channel Selection for EOG-Based Gaze Displacement Estimation

Nathaniel Barbara, Tracey A. Camilleri, Kenneth P. Camilleri
University of Malta, Malta
nathaniel.barbara@um.edu.mt

Electrooculography (EOG)-based gaze estimation conventionally relies on the processing of two bipolar EOG channels, obtained from two electrodes attached near the outer canthi and two electrodes attached above and below one eye, commonly referred to as the horizontal and vertical channels, respectively. This work aims to systematically investigate whether alternative pairs of bipolar EOG channels computed from 14 candidate electrodes around the eyes can result in more accurate gaze displacement estimation. In this work, each bipolar EOG channel is represented by the angle of elevation relative to the horizontal axis of the line connecting the constituent two electrode positions after they are projected onto the face-plane. While the conventional horizontal and vertical bipolar EOG channels have an ideal elevation of 0° and 90°, respectively, variations in electrode attachment across recording sessions may lead to an average variability of less than 5° when using these channels. This work shows that in comparison to using the conventional horizontal and vertical channels, a statistically significant ($p<0.05$) improvement in gaze displacement estimation performance is commonly achieved across eight subjects using bipolar EOG channels predominantly with elevations of 20°-60° coupled with 155°-180°, 40°-60° coupled with 110°-150°, and 110°-130° coupled with 165°-180°.

Validation of remote eye-tracker technology as a vehicle for investigation of language comprehension processes in the absence of a stable head position

Heather Graz, Alissa Melinger, Annalu Waller
University of Dundee, United Kingdom
2446733@dundee.ac.uk

Eye tracking has provided valuable insights into language processing mechanisms. Its application in populations who experience difficulty maintaining a stable head position is, however, untested. People with severe speech and physical impairments (SSPI) are one such population.
This paper presents original research carried out to validate use of a remote eye-tracking set-up for comparison of language comprehension processing in adults with SSPI and a neurotypical control group, in a between groups experimental design.
A Cued Attention prosaccade visual search task as per Posner (1980) was designed as proof-of-concept of eye movement timing and patterns across groups. A spoken language comprehension task was later presented within a Visual World Paradigm (VWP) framework.
Broadly similar scanpaths were seen across the experimental SSPI group and neurotypical control group, both in the proof-of-concept Cued Attention task and the subsequent spoken language comprehension task. Automatic calibration yielded comparable results across groups. Overall patterns of accuracy and precision were comparable across groups, with less variance in the control group. Recovery time was similar, but eye gaze recovery was required more often in SSPIs. Individual-specific eye movement differences notwithstanding, remote eye-tracking is a valid technology to study language processing in people with SSPI.

How to record 140° gaze shifts?

Ignace T.C. Hooge[1], Diederick C. Niehorster[2], Marcus Nyström[2], Roy S. Hessels[1]
[1]Utrecht University, The Netherlands; [2]Lund University, Sweden
i.hooge@uu.nl

We have developed a new eye tracking setup capable of capturing large head-gaze shifts, reaching up to 140°. This setup comprises a wearable eye tracker (Pupil invisible) along with a high-speed camera attached to the ceiling. The participant was equipped with a hat with a fiducial marker to monitor head movements. To assess its effectiveness, we conducted experiments replicating established findings from the literature on eye-head gaze shifts. We found that this cost-effective setup sufficiently facilitates the exploration of the dynamics involved in large eye-head gaze shifts. Besides its applicability to studying such gaze shifts, this setup is suitable for investigating gaze behavior in various contexts, such as human interaction. Additionally, we discuss world and head-centered reference frames and terminology concerning head-free eye tracking. Despite the shift from head-fixed eye tracking to head-free gaze tracking, there persists a tendency among researchers to use terminology associated with head-fixed eye movements when discussing gaze phenomena relative to the world. To address this, we suggest employing more precise terminology, including terms such as gaze fixation, gaze pursuit, and gaze saccade, to describe world-fixed phenomena accurately.

A Concurrent Comparison of EyeLink 1000+ and TrackPixx3 on a Benchmark Test Battery

Anna Pryslopska, Benedikt Ehinger, Titus von der Malsburg
University of Stuttgart, Germany
anna.pryslopska@ling.uni-stuttgart.de

The EyeLink 1000+ eye-tracker by SR Research has become the gold standard for research on eye movements. Recently, a new eye-tracker, VPixx' TRACKPixx3, has made its debut. This binocular eye-tracker is desktop-mounted and records at up to 2 kHz, like EyeLink. While the strengths and weaknesses of EyeLink are well-understood, there is no comprehensive evaluation of TRACKPixx3 and only few published studies used it.
We assessed these eye-trackers through the concurrent recording of eye movements across six tasks: grid viewing, free viewing, smooth pursuit, microsaccades, blink detection, pupil dilation (Ehinger et al., 2019). They were designed to evaluate aspects such as decay of accuracy, fixation durations, pupil dilation, smooth pursuit movement, microsaccade classification, blink classification. Additionally, we included a new text reading task.
Data from 27 participants indicated that the performance of both eye-trackers is largely similar. The winsorized average spatial accuracy and the fixation counts were nearly the same. We detected shorter fixations and more blinks in TRACKPixx3 data, whereas EyeLink registered longer saccades. TRACKPixx3 recorded smoother data, potentially due to an internal filter. In sum, although EyeLink continues to be the benchmark, TRACKPixx3 presents itself as a serious alternative worth considering.

Webcam based eye-tracking -Validation Study Report

Divya Prakash Seernani, Morten Mosbaek Pedersen, Kerstin Wolf
iMotions A/S, Denmark
divya.seernani@imotions.com

The purpose of the validation study was to run a large scale study to evaluate how the iMotions WebET 3.0 algorithm performs on a truly diverse, global, sample, "in-the-wild". Data was globally collected from 255 participants over 35 days. Participants conducted a short study comprising of gifs, images, videos, and surveys. Self-reported parameters for ethinicity, eye-color, wearing glasses or not, having facial hair or not, and self-reported lighting conditions in the room were evaluated against accuracy. Over 50% of participants had an accuracy of 2 degrees of visual angle (dva) or lower. Over 70% had an accuracy of 3dva or lower and over 90% had an accuracy of 5dva or lower. Of the parameters measured, only the presence of glasses had a significant effect on accuracy. Over time, fixation classification stays stable in the center of the screen but classification may reduce in accuracy towards the bottom corners of the screen. Longer studies and internet problems can cause problems with participant compliance and a suboptimal user experience. The individual differences for ethinicities, regions, eye-colour and the presence of facial hair did not have a significant impact as people collected data in their natural environments, indicating an unbiased dataset on a demographic level.

Tuesday - morning - Parafoveal Processing in Reading - Venue A

Neural correlates of parafoveal n+1 and n+2 word processing during sentence reading

Petar Filipov Atanasov[1], Simon P. Liversedge[1], Federica Degno[2]

[1]University of Central Lancashire, United Kingdom; [2]Bournemouth University, United Kingdom;
patanasov@uclan.ac.uk

Multiple eye-tracking studies have investigated to what extent readers may be able to parafoveally process word *n+2* at the point of fixating on word *n*. Evidence on the topic is mixed and the implications of existing findings concerning how readers lexically process words are still debated. In the present study, participants' eye movements and fixation-related potentials (FRPs) were simultaneously recorded to examine whether *n+2* effects could be observed at a behavioural and/or neural level. One-line sentences were presented using the boundary paradigm (Rayner, 1975), and two target words were manipulated, such that their preview could be identical, one or both target words could be masked by a string of random letters, or the two target words could be transposed. The eye movement data showed that disruption to reading occurred mainly during the processing of word *n+1* and that an invalid preview of *n+1* largely drove this disruption consistent with an *n+1* parafoveal preview effect. Effects were also observed at word *n+2*, but again these likely reflected processing difficulties of word *n+1*. FRP data are currently being analysed. Implications for the parallel versus serial lexical processing debate will be discussed.

Relating foveal and parafoveal word processing efficiency with eye-movement measures of reading Chinese

Timo T. Heikkilä[1], Yongsheng Wang[2,3,4], Chuanli Zang[2,3,4,5], Xin Li[2,3,4], Jukka Hyönä[1]
[1]University of Turku, Turku, Finland; [2]Key Research Base of Humanities and Social Sciences of the Ministry of Education, Academy of Psychology and Behavior, Tianjin Normal University, Tianjin, China; [3]Faculty of Psychology, Tianjin Normal University, Tianjin, China; [4]Tianjin Social Science Laboratory of Students' Mental Development and Learning, Tianjin, China; [5]University of Central Lancashire, Preston, UK; tithei@utu.fi

We examined the relationship between the processing efficiency of single- and two-character words presented in fovea and parafovea and word-level eye-movement parameters in reading Chinese among a heterogenous sample of 70 adult readers (age range 18-60 years). Foveal processing efficiency was assessed with performance accuracy in lexical decision, where one- and two-character words of medium frequency and nonwords were presented backward-masked for identification using different exposure times (20-60ms). Parafoveal processing efficiency was assessed by presenting similar stimuli for 150ms to the right and left of the central fixation point (1.33-2.24 degrees of visual angle). Measures of foveal and parafoveal processing efficiency were used to predict eye-behavior in reading 8 expository texts. The Random Forests technique was used to assess the relative importance of individual difference measures in predicting readers' eye-movement patterns. The results show that the ability to recognize two-character words in fovea best predicts gaze durations in words. Word skipping was best predicted by the efficiency in foveally recognizing one and two-character words and also by the efficiency in parafoveally recognizing. two-character words. The results are consistent with an analogous study conducted in Finnish, which found that efficiency in foveal word processing best predicts gaze durations and word skipping.

What's up with "the"? – Co-registered ET-EEG investigation of parafoveal syntax processing

Otto Loberg[1], Bernhard Angele[2], Federica Degno[1]
[1]Bournemouth University, United Kingdom; [2]Nebrija University, Spain; oloberg@bournemouth.ac.uk

Word skipping is essential for efficient reading. "The" is the most frequent word in English, appearing at least once in most English sentences, and is, accordingly, skipped extremely often. This happens even when the gaze-contingent boundary paradigm is used to show previews of "the" in positions that would be syntactically inappropriate. One possible explanation is that the syntactic properties of "the" are not processed early enough to prevent the word from being skipped. The present study aimed to test this hypothesis by manipulating the preview of a three-letter target word in a single-line sentence. The preview could be the target word itself (identity preview), a meaningless string of letters, a word semantically related to the target, or the syntactically illegal "the". Participants' eye movements and Fixation Related Potentials (FRPs) were simultaneously recorded. Preliminary results showed a target word was skipped more often when its preview was the syntactically illegal "the" compared to all other preview conditions. However, we found no difference in the FRPs time-locked to the pretarget word between identity and "the" previews, suggesting that parafoveal syntactic information is either suppressed or not processed. We discuss the implications of these findings on theories of eye movements of reading.

Parafoveal processing of morphological structure for deaf and hearing readers

Emily Saunders[1], Elizabeth Schotter[2], Karen Emmorey[1]
[1]San Diego State University; [2]University of South Florida
ecsaunders@sdsu.edu

Parafoveal processing of morphological structure is observed in morphologically rich languages, but evidence is mixed for English; parafoveal preview benefit from morphological relationships is observed for suffixed words, but not compound or prefixed words. Morphological information in English may be particularly accessible for deaf readers due to the consistent mapping between orthography and semantics. Using a gaze contingent display change paradigm, we tested whether deaf and hearing readers with varying morphological awareness (MA) skill showed differences in parafoveal processing of morphology during sentence reading. When participants' eyes crossed a boundary, a preview was replaced by a morphologically complex target word (i.e., sadness). Preview words were in one of four conditions: 1) identical (sadness), 2) pseudomorphological nonword (sadment), 3) nonmorphological nonword (sadnard), 4) unrelated nonword (florous). Preliminary data from an ongoing experiment (16 deaf, 19 hearing) showed a marginally significant interaction between group and the contrast between conditions 2 and 3 for gaze duration ($p = 0.056$). This effect was driven by deaf readers with high MA skill, who processed target words faster if the preview contained a pseudomorphological ending. This result suggests that morphological awareness differentially impacts deaf readers' parafoveal processing because of the visual accessibility of morphology in English.

Parafoveal preview of short words during reading and skimming

Shi Hui Wu[1], Kayleigh L Warrington[2], Erik D Reichle[3], Kevin B Paterson[2], Sarah J White[2]
[1]University of Edinburgh, United Kingdom; [2]University of Leicester, United Kingdom; [3]Macquarie University, Australia
swu34@ed.ac.uk

Parafoveal processing is central to the mechanisms underlying eye movement control during reading for comprehension. However, little is known about the role of parafoveal preview during skimming for gist. This OSF-preregistered study utilized a gaze-contingent boundary paradigm to assess how task demands can affect parafoveal processing of short words. 64 participants read or skimmed sentences containing a 3-letter low-frequency critical word (e.g., nap), for which the preview was either identical or visually-similar (e.g., noy). For first-pass measures there were additive effects of task and preview: for both tasks, words were more likely to be skipped, and had shorter single fixations when receiving an identical preview. In contrast, there were larger preview effects during reading for comprehension for later measures (e.g., regression path duration). The results indicate that the effects of parafoveal preview on initial processing of short words can be similar during reading and skimming, but task demands can modulate effects of preview on subsequent rereading behaviour. We also employed a lexical decision cross-context repetition priming paradigm to examine whether reading task can modulate continued lexical activation of words after reading. The implications for developing theoretical accounts of the mechanisms underlying eye movement control during skimming will be discussed.

The influence of foveal processing load on parafoveal preview extent in Chinese reading

Simon P. Liversedge[1], Manman Zhang[2], Zhichao Zhang[2], Fang Li[2], Xuejun Bai[2], Chuanli Zang[1]
[1]University of Central Lancashire, United Kingdom; [2]Tianjin Normal University, China
SPLiversedge@uclan.ac.uk

Using the boundary paradigm, we report two experiments to investigate how foveal lexical processing load (high/low frequency) of a pretarget word influences preview extent (zero, one, two or three parafoveal characters, or the full sentence) of upcoming target word(s) in Chinese reading. In Experiment 1, three characters comprised a single target word while in Experiment 2 they formed multiple words (two or three words). Pretarget word analyses showed an effective foveal load manipulation with low frequency pretargets being fixated for longer than high frequency pretargets. Target analyses showed that with increased preview extent, fixation durations were reduced. The magnitude of these effects in Experiment 1 (130 participants) was numerically greater under low- than high-foveal load conditions with robust differences when the preview was limited to zero or one character extent. However, such modulation of preview extent by foveal load was not evident in Experiment 2 (120 participants). These results indicate that the lexical status (one word or multiple words) of the upcoming word might modulate the foveal load effect on parafoveal processing extent during Chinese reading. We will discuss results from a large-scale replication of the two experiments with increased power (by doubling the sample size) in the talk.

Tuesday - morning - Eye Movement Data Analyses - Venue B

Systematic Drift Correction in Eye Tracking: Integrating Line Assignments with Implicit Recalibration

Wolf Culemann, Angela Heine
University of Duisburg-Essen, Germany; wolf.culemann@uni-due.de

The quality of eye tracking data is often compromised by systematic error, commonly referred to as drift. Most manual and automated approaches to drift correction have limitations, such as only correcting for the y-dimension, thus neglecting horizontally misaligned fixations. Available approaches that allow for horizontal correction, involve inferring the systematic error from probable fixation locations, but have not been used in conjunction with alignment algorithms. In this talk, we discuss the similarities and differences between alignment and implicit recalibration. Furthermore, we present a new approach for extracting the inherent systematic error across multiple multiline reading trials. While our approach is based on an initial trial-by-trial line-to-word assignment using dynamic time warping, it can be based on other alignment algorithms as well. The systematic part of the drift is then extracted by spatial and temporal filtering to reduce artificial noise. Preliminary results show that this method reduces drift in both the horizontal and vertical dimensions significantly, as shown by comparisons with manual ground-truth alignments and validation grids. This approach has the potential to add value to both the automated and, by speeding up and streamlining, the manual alignment process.

eyeScrollR: A software method for reproducible mapping of eye tracking data from scrollable web pages

Nathanael Larigaldie[1], Anna Dreneva[1], Jacob L. Orquin[1,2]
[1]Aarhus University, Denmark; [2]Reykjavik University
larigaldie.n@mgmt.au.dk

An increasing number of researchers use eye tracking technology to examine attention and behavior in online environments. Researchers, however, face a significant challenge in mapping eye tracking data from scrollable web pages. We describe the R package eyeScrollR for mapping eye tracking data from scrollable content such as web pages. The package re-maps eye tracking gaze coordinates to full page coordinates with a deterministic algorithm based on mouse scroll data. The package includes options for handling common situations, such as sticky menus or ads that remain visible when the user scrolls. We test the package's validity in different hardware and software settings and on different web pages and show that it is highly accurate when tested against manual coding. Compared to current methods, eyeScrollR provides a more reproducible and reliable approach for mapping eye tracking data from scrollable web pages. With its open code and free availability, we recommend eyeScrollR as an essential tool for eye tracking researchers, particularly those who adhere to open science principles. The eyeScrollR package offers a valuable contribution to the field of eye tracking research, facilitating accurate and standardized analysis of eye tracking data in web scrolling contexts.

What is a blink? Classifying and characterizing blinks in eye openness signals

Marcus Nyström[1], Richard Andersson[2], Diederick C. Niehorster[1], Roy S. Hessels[3], Ignace T. C. Hooge[3]
[1]Lund University, Sweden; [2]Tobii AB, Sweden; [3]Utrecht University, the Netherlands;
marcus.nystrom@humlab.lu.se

Post-microsaccadic eye movements recorded by high frame-rate pupil-based eye trackers reflect movements of different ocular structures such as deformation of the iris and pupil-eyeball relative movement as well as the dynamic overshoot of the eye globe at the end of each saccade. These Post-Saccadic Oscillations (PSO) exhibit a high degree of reproducibility across saccades and within participants. Therefore in order to study the characteristics of the post-saccadic eye movements, it is often desirable to extract the post-saccadic parts of the recorded saccades and to look at the ending part of all saccades. In order to ease the studying of PSO eye movements, a simple tool for extracting PSO signals from the eye movement recordings has been developed. The software application implements functions for extracting, aligning, visualising and finally exporting the PSO signals from eye movement recordings, to be used for post-processing. The code which is written in Python can be download from https://github.com/dmardanbeigi/PSOVIS.git

EyeFeatures: the package for preprocessing, visualisation, statistical and machine learning analysis of eye movement data

Anton Surkov, Vagiz Daudov, Daniil Tikhonov, Zakhar Kondaurov, Elena Artemenko
HSE University, Social and Cognitive Informatics lab., Russian Federation
aysurkov@hse.ru

EyeFeatures is an open-source Python package for analyzing eye movement data in any visual task (https://github.com/hse-scila/EyeFeatures). Its capabilities encompass preprocessing, visualization, statistical analysis, feature engineering and machine learning. Its unique feature is its architecture and versatility. Accepting data in .csv format containing gaze position coordinates, the package allows filtration of raw data to remove noise and detecting fixations and saccades with different algorithms. Having fixations any standard descriptive statistical eye movement features (such as totalFD, meanFD etc.) can be computed, including AOI-wise features. AOIs can be predefined or assigned automatically. More complex features, such as chaos measures, topological features, density maps, scanpath similarities for various distance metrics can be computed as well. The package allows to account for the panel structure of the data, calculating shift features relative to group averages. The visualization module allows output a variety of visualization options, including static and dynamic scanpath plots, customized heatmaps and histograms. The architecture of the package allows seamless embedding of its preprocessing and feature extraction classes in Sklearn pipelines. Moreover, it provides datasets and models for deep learning with Pytorch. Since the work is in progress all functionality will be implemented by the time of the report.

Analyzing large mobile eye tracking datasets: An example from educational research

Senne M. Van Hoecke
Open University, Netherlands
senne.vanhoecke@ou.nl

Managing a classroom is a key competence of teachers that enables students' learning (Berliner, 2001). Effective classroom management requires a teacher to monitor visual cues, notice and interpret them in time and act accordingly. While this skill is invaluable in teaching, novice teachers seem to struggle with it (van Es & Sherin, 2002). Previous research has already revealed some eye movement differences between experienced and beginning teachers, though further research into the matter is required. However, when conducting eye tracking research in authentic classroom settings, the datasets produced are generally immense, dynamic and hard to analyze. The question thus arises: How can a similar sizable, dynamic eye tracking dataset be analyzed?

Van Driel et al. (2023) collected one of the biggest eye tracking datasets recorded in authentic classroom settings. Data from 58 secondary school teachers were collected using SMI eye tracking classes. The resulting 216.95GB of data amounted to 44 hours of video. To analyze this data, each classroom was time-coded based on the activity and/or position of the teacher. The present talk shows how such a time and activity-based approach can offer insight into eye movements. Machine learning is used to support the results from this approach.

Streamlining Scarf Plot Generation for Eye-Tracking Research

Michaela Vojtechovska, Stanislav Popelka
Palacký University Olomouc, Czech Republic
mail@vojtechovska.com

Scarf plots are pivotal in eye-tracking visualization, revealing how individuals allocate their visual attention to areas of interest (AOI) over time, thus providing valuable insights into cognitive processes and task performance. However, their creation typically demands proficiency in programming languages like R, Python, or Matlab. In addition, existing software solutions lack advanced customization options and support for dynamic AOIs while necessitating upfront data transformations by users.

To address these challenges, we have developed GazePlotter - a tool for automatic generation of scarf plots. Freely available on https://gazeplotter.com as a multiplatform SvelteKit web app, GazePlotter constructs adaptable scarf plots from various eye-tracking software exports, including Tobii Pro Lab, SMI BeGaze, or GazePoint Analysis. Researchers can create multiple scarf plots in the workspace, allowing the comparison of data on three timeline types for various participant groups and stimuli side-by-side. Moreover, researchers can upload dynamic AOIs and visualize their visibility to participants during an experiment.

With a user-friendly interface that eliminates the need for programming expertise, GazePlotter makes scarf plots more customizable and accessible to researchers. In ongoing development, we sincerely invite feedback and collaboration on the open code to foster the development of the new features in upcoming releases.

Tuesday - morning - Eye Tracking in (near) Natural Settings - Venue C

Yarbus Revisited: The Webcam Edition

Leen Catrysse[1], Marijn Gijsen[2,3], Margot van Wermeskerken[4], Halszka Jarodzka[1]
[1]Open Universiteit, Netherlands; [2]Tilburg University, Netherlands; [3]University of Antwerp, Belgium; [4]Utrecht University, Netherlands
leen.catrysse@ou.nl

This study explores the viability of webcam-based eye-tracking as an alternative or adjunct to traditional eye-tracking methods in educational research. Replicating Yarbus's seminal study, participants viewed a painting under varied instructions while their gaze patterns were recorded with their webcams using the iMotions platform. Out of 53 participants, 26 yielded usable data. We compared eye-tracking metrics for two different instructions: (1) estimate ages and (2) remember positions of people and objects. The painting was divided into four AOIs of equal size. For each AOI, fixation count and fixation duration were calculated. In addition, the GINI coefficient (GC) was calculated per instruction which is a measure of inequality of gaze distribution. Results from mixed effects models revealed significant differences in fixation counts and durations for some AOIs between the two instructions. In addition, the GC showed a significantly higher inequality in gaze distribution for age estimation compared to recall of positions of people and objects. Findings are in line with results from traditional lab studies. The findings support the utility of webcam-based eye-tracking in replicating established links between gaze and task demands, offering potential for wider application in online experimentation within educational sciences.

Pupil-driven music machine

Leonardo Cardinali, Livia Colucci, Silvestro Roatta
Dept of Neuroscience, University of Torino
livia.colucci@unito.it

A number of studies have demonstrated the potential for utilizing the “voluntary” control of the pupil in the development of Augmentative and Alternative Communication (AAC) systems, with a particular emphasis on leveraging the Pupil Accommodative Response (PAR). However, the naïve user may be little aware of PAR characteristics such as latency and speed of pupil constriction, which may impair accuracy and effectiveness in communication. Consequently, there is a need of tools that support user training and engagement.
In this study, we introduce a new application of the PAR that utilizes the pupil diameter to create a melody. The variation in pupil diameter is controlled by voluntarily shifting the focus from a distant to a closer target, and the ensuing degree of pupillary constriction is associated with a musical note. To enhance the appeal of the system, the selected notes, that belong to a pentatonic scale, are played over a background jazz track. Preliminar experiments in healthy subjects confirmed the feasibility of the system and stimulated great interest and enjoyment among participants. This tool holds promise for both training and entertainment of healthy individuals and patients in the need of AAC.

Navigating the Sea of Eco-Labels: Unveiling Consumer Attention through Eye-Tracking Analysis on Italian Aquaculture Products

Migena Proi[1,2], Emilia Cubero Dudinskaya[1], Simona Naspetti[3], Raffaele Zanoli[1]
[1]Department of Agricultural, Food and Environmental Sciences - Università Politecnica delle Marche, Italy; [2]Department of Bioscience and Agro-Food and Environmental Technology - Università degli Studi di Teramo; [3]Department of Science and Engineering of Matter, Environment and Urban Planning - Università Politecnica delle Marche, Italy
e.cuberod@agrecon.univpm.it

Consumers growing interest in sustainability has led to a proliferation of eco-labels in food products. However, the efficacy of these labels is hindered by the visual overload consumers encounter when making food choices. This study uses eye-tracking technology to investigate how consumers allocate visual attention to five eco-labels in the Italian context. Visual attention is measured sequentially, providing information on how top-down (e.g., health involvement) and bottom-up (e.g., saliency) factors affect visual attention at different times. Unlike previous investigations focused on eco-labels from agricultural products, our study explores eco-labels found in aquaculture products, a crucial area for sustainability due to marine stock overexploitation.
Results showed that eco-label noticeability was mainly determined by consumers' motivational mindset, such as the need for information, health concerns, and interest in food naturalness. Saliency emerges as a critical factor in maintaining consumer interest in eco-labels. Meanwhile, visual recognition has no significant effect. Findings suggest the importance of educational campaigns to increase consumers' knowledge and awareness of health aspects, making eco-labels more likely to capture attention. Simultaneously, promoting the visual saliency of eco-labels can aid in retaining consumers' attention. Finally, increased advertising of eco-labels is recommended to boost awareness

Consumer search patterns in online retailing

Anna Dreneva[1], Jacob Orquin[1,2]
[1]Aarhus University, Denmark; [2]Reykjavik University, Iceland;
annadrenyova@gmail.com

Most online retail studies lack ecological validity due to using artificial stimuli, such as simulated websites, webpage screenshots, or separate product images. In our study, we used a live online grocery website to investigate consumers' search strategies in conditions close to realistic. A diverse cohort of 121 Danish citizens were to select a product from 12 categories. For recording eye movements, the Aurora smart eye eye-tracker 120 Hz was used. Since the participants had scrolled webpages up and down while searching, we converted the collected fixations from screen coordinates to webpage coordinates using the innovative eyeScrollR R package. Then, we identified seven types of behavioral events: examination, comparison, systematic or non-systematic scanning, scrolling up, scrolling down, and purchase. Based on the differences across event sequences, we clustered search strategies into three clusters: Fast, Intermediate, and Slow. Each cluster presented a unique online search strategy type comprising various event numbers, observed product numbers, and the average purchased product's locations. The binomial logistic regression results showed a highly significant influence of both cluster and product location on purchase probability ($p<.0001$). The findings can be used to develop targeted marketing strategies to contribute to consumer welfare.

Usability verification of several gaze control methods of the Breakout game

Katarzyna Kotyńska, Jacek Matulewski
Nicolaus Copernicus University, Poland
jacek@phys.uni.torun.pl

The usability study discussed in the talk focused on evaluating a gaze-controlled classic Breakout game with an adaptive difficulty level. Additionally, the study considered the participation of a disabled person who solely uses gaze for communication and environmental control. Four variants of the gaze control method were tested, which included two modifications: the method by which a paddle follows the gaze and the restriction of the gaze-aware area.
The results of the study revealed:

1. The discrepancy in player preferences and performance: the discrepancy between what gaze control method players preferred and which method led to better game scores. It suggests that player preferences may only sometimes align with optimal performance outcomes.
2. Impact of limiting gaze interaction area: the study found that restricting the gaze interaction area negatively impacted objectively and subjectively assessed usability. It indicates that limiting the area within which gaze control operates can hinder player experience and effectiveness in controlling the game.

Impact of data quality on driving performance related eye-tracking measures

Raimondas Zemblys[1], Christer Ahlström[2,3], Svitlana Finér[1]
[1]Smart Eye AB, Sweden; [2]Swedish National Road and Transport Research Institute (VTI), Linköping, Sweden; [3]Department of Biomedical Engineering, Linköping University, Linköping, Sweden
raimondas.zemblys@smarteye.se

In this study, participants drove in a vehicle on a test-track, first while sober and then under the influence of alcohol. Three distinct camera setups were used: a five-camera system representing an idealized research configuration, and two single-camera systems similar to those found in production vehicles. The objective was to analyse how different eye-tracking measures were affected by intoxication and whether these effects could be reliably estimated in realistic recording scenarios.
The results show that glance duration proportions were highly sensitive to data loss. Some glance metrics even lost their relation with intoxication when assessed using single-camera systems. The impact of different setups was mostly noticeable in absolute values of psychophysiological measures. Nevertheless, effects of intoxication could be measured and were similar in all three setups. As intoxication levels increased, driver behaviour was altered in a way that negatively impacted data quality in both single-camera systems.
The results imply that lab findings based on higher quality eye-tracking systems are not directly applicable in production settings. While general trends may still hold across different camera types and placements, the absolute values may deviate significantly. Researchers and developers working on intoxication detection systems should account for data loss and noise when using eye-tracking metrics.

Tuesday - afternoon - Word Processing in Reading - Venue A

The effects of reading ability and vocabulary on incidental word learning during reading: An eye movement investigation

Emily J. Bellerby[1], Sara V. Milledge[2], Kristofor McCarty[1], Hazel I. Blythe[1]
[1]Northumbria University, United Kingdom; [2]University of Central Lancashire, United Kingdom
emily.bellerby@northumbria.ac.uk

A large proportion of an individual's vocabulary is learned incidentally, during reading. We examined individual differences in lexical acquisition during reading and compared the processing of pseudowords and low-frequency words during lexical acquisition. Participants' eye movements were measured as they read sentences each containing a novel word (low-frequency or pseudoword). Each novel word was presented in eight meaningful sentences, providing a diverse semantic context. Individual, standardised assessments of both reading ability and vocabulary were also collected. Two measures of lexical acquisition were employed (1) eye-movement recordings, to provide an index of the ease with which participants were able to read the novel words, and (2) a semantic categorisation task, to examine whether participants had successfully formed semantic representations for the new words. No significant interactions were found between phase and word type, suggesting lexical acquisition during reading did not differ between pseudowords and low-frequency words. Analyses show that individual differences in reading ability, but not vocabulary, are associated with successful lexical acquisition. This research validates the use of pseudowords in word-learning experiments. Furthermore, we show that individuals with stronger reading skills, as measured by standardised tests, are more successful in instantiating new lexical representations as they read.

Do readers not fully process "not"? An eye movement investigation of the processing of negated statements

Victoria A. McGowan[1], Shi Hui Wu[2], Anna M. Plunkett[1], Meyrem Tompson[1], Faye O. Balcombe[1], Summayah Mullick[1], Kevin B. Paterson[1], Sarah J. White[1]
[1]University of Leicester, United Kingdom; [2]University of Edinburgh, United Kingdom
vm88@le.ac.uk

Statements containing negations ("no" or "not", e.g., "There are no cookies in the jar") are more difficult to comprehend and accurately remember than non-negated statements. This cost is typically attributed to a two-step account, in which a negated statement is initially represented as positive ("There are cookies in the jar") before being represented as negative ("There are no cookies in the jar"). However, another possibility is that eye movement behaviour (specifically word skipping) may contribute to difficulties in comprehending negations. Indeed, the length and frequency of negations mean that they are likely to be frequently skipped, and so may not be fully processed. Accordingly, this experiment aimed to reveal: a) how skipping affects the processing of negations, and b) whether increasing the visual salience of negations reduces skipping and thus facilitates their processing. In an eye tracking study, 60 participants read sentences containing either no negations, a negation in a standard presentation format, or a negation that was bold and underlined to increase visual saliency. Comprehension accuracy was tested immediately, and after a delay. The implications of the findings for our understanding of how skipped words are processed will be discussed.

Eye Movements and Noisy-Channel Inference-Making during Reading

Michael Cutter[1], Ruth Filik[1], Kevin Paterson[2]
[1]University of Nottingham, United Kingdom; [2]University of Leicester, United Kingdom
kbp3@le.ac.uk

Current models of sentence processing assume that perceptual input to the language-processing system will contain errors. Because of this, readers are assumed to make “noisy-channel” inferences that modify this input (e.g., by adding, deleting or re-ordering words) to achieve a plausible interpretation. However, the extent to which these perceptual errors are a consequence of how readers visually sample text is unclear. Accordingly, we investigated the relationship between readers’ eye movements and their use of “noisy-channel” inferences when reading implausible sentences. Young (18–26 years) and older (65–87 years) participants read sentences which were either plausible or implausible. Crucially, readers could assign a plausible interpretation to the implausible sentences by inferring that a preposition (i.e., “to”) had been unintentionally omitted or included. Our results reveal that readers’ fixation locations within such sentences are associated with the likelihood of them inferring the presence or absence of this critical preposition to reach a plausible interpretation. Moreover, the older adults were more likely to make these noisy-channel inferences than the younger adults, potentially because their poorer visual processing and greater linguistic experience promote such inference-making. We argue that these findings provide novel experimental evidence for a perceptual contribution to noisy-channel inference-making during reading.

Article-noun agreement in the parafovea does not affect skipping in Spanish

Marina Serrano-Carot, Bernhard Angele
Universidad Antonio de Nebrija, Spain
mserranoc6@alumnos.nebrija.es

This study investigates how Spanish native speakers utilize parafoveal information to guide eye movements during reading. Readers often skip short and common words, like the article "the" in English, even when encountering a preview of the word that conflicts with the preceding context. This implies that the oculomotor system prioritizes parafoveal information over contextual cues. However, this may not hold true when both the high-frequency word and the conflicting information are in the parafovea. In Spanish, there is mandatory gender agreement between articles and nouns. We manipulated the preview readers received of the article in article-noun phrases such that there either was agreement between the article and the noun (e.g. “la mesa”) or not (e.g. “el* mesa”). After readers crossed an invisible boundary at the left of the article the display changed to show the correct article. We find that readers do not take article-noun agreement into account when making the skipping decision; however, the mismatch affects fixation times on the noun and the subsequent word. This suggests that either parafoveal preprocessing before skipping exclusively involves the next word (the article) or parafoveal processing is very superficial and does not involve higher-level information as syntactic relationships between the upcoming words

The processing of the definite article in Brazilian Portuguese: When “the” carries gender and number marking

João Vieira[1], Elisângela Teixeira[2], Denis Drieghe[1]
[1]University of Southampton, UK; [2]Federal University of Ceará, Brazil
joao.vieira@soton.ac.uk

Two eye movement studies in reading in English indicate special status of the definite article “the” compared to content words. Angele and Rayner (2012), using a gaze-contingent paradigm, reported that when the preview of a word was a grammatically incorrect "the", it was skipped more often than a grammatical continuation of the sentence. Staub et al. (2018) found that readers noticed the repetition of "the" less frequently than the repetition of content words. We repeated both studies in Brazilian Portuguese (BP), where compared to English, definite articles carry more information in the form of gender and number marks. In a gaze-contingent experiment, we found that the preview of an ungrammatical definite article was skipped more often than a grammatical continuation, replicating Angele and Rayner. The mechanism of automatically skipping an article – even when it is grammatically incorrect – is also present in BP potentially because gender and number marking is typically repeated in the next word. However, contrary to Staub et al., repeated definite articles were noticed nearly as frequently as content words. When directly fixated, definite articles in BP have too much semantic/syntactic information for them to be ignored.

Predictability effects in Chinese reading: Evidence from eye movements during corpus reading

Roslyn Wong[1], Jin Tian[1], Lili Yu[1], Aaron Veldre[2], Erik D. Reichle[1]
[1]Macquarie University; [2]University of Technology Sydney
roslyn.wong@mq.edu.au

It is well-established that contextual predictability influences the probability and duration of fixations on words when reading alphabetic scripts like English. However, comparatively less is known about these effects in non-alphabetic scripts like Chinese, where text is written as strings of characters with no spacing cues to demarcate word boundaries. The present research reanalysed the eye-movement data of 60 participants who read a corpus of Chinese sentences for which the predictability of each character and word were estimated by the cloze responses provided by separate samples of participants. Linear mixed-effects models assessed the relative contributions of character- and word-based probabilities, as well as the impact of partial syntactic and semantic information, on readers’ eye movements across the time course of lexical and post-lexical processing. The results provide insights into the functional units that influence predictability effects in Chinese reading. The implications for theories of prediction in language comprehension and models of eye-movement control across different writing systems will be discussed.

Tuesday - afternoon - Clinical Eye Movement Research - Venue B

Eye-tracking During Naturalistic Free Viewing Allows for Highly Specific Classification of Parkinson's Disorder

Donald C Brien[1], Heidi C Riek[1], Brian Coe[1], Brian White[1], Isabell C Pitigoi[1], Jeff Huang[1], David Grimes[2], Anthony Lang[3], Connie Marras[4], Mario Masellis[5], Douglas P Munoz[1]
[1]Centre for Neuroscience Studies, Queen's University, Canada; [2]Ottawa Hospital, Ottawa Brain and Mind Research Institute, Canada; [3]Edmond J. Safra Program in Parkinson's Disease, University Health Network and the Department of Medicine, Division of Neurology, University of Toronto, Canada; [4]Toronto Western Hospital Movement Disorders Centre and Edmond J Safra Program in Parkinson's Disease, University of Toronto, Canada; [5]Sunnybrook Health Sciences Centre, Toronto, Canada
briend@queensu.ca

Neurodegenerative disorders are the leading cause of disability worldwide. In particular, the incidence of Parkinson's disease (PD) is expected to double by 2040. Neurodegeneration occurs years before symptomatic presentation of PD, suggesting that biomarkers could be detected in the early stages, allowing for early identification and potentially personalized therapeutics. Eye-tracking has proven to be a sensitive proxy for the neurodegeneration occurring in PD. Our recent work has shown that a structured interleaved pro-/anti-saccade task (IPAST) can identify cognitive subtypes of PD and classify PD with an accuracy of 81% and ROC-AUC of 88%. Here, we extend our classification work by adding eye-tracking features extracted during 10-minutes of a naturalistic free-viewing task (FV) in a diverse population of early-stage PD (N=113) and age-matched controls (N=89). A machine learning model was developed by augmenting IPAST features with new saccade, pupil, and blink features extracted from the FV task. This model was able to classify PD with an accuracy of 92% on a held-out test set, and an AUC-ROC of 94% (+/- 5%) on cross-validated training data. We demonstrated that features extracted from the FV task allow the creation of highly sensitive machine-learning models of PD, paving the way for clinical utility.

Attention deficit and hyperactivity disorder disrupts selective mechanisms of action as revealed by eye movements

Olivier A. Coubard
The Neuropsychological Laboratory CNS-Fed; olivier.coubard@cns-fed.com

People with attention deficit and hyperactivity disorder (ADHD) present attentional and emotional deficits and show paradoxical qualities such as hyperfocus. Using eye movements, previous studies have reported errors, slowness, and reaction time (RT) variability. The goal of this study was to explore the underlying mechanisms of ADHD further. Thirty French children and teenagers, 15 with ADHD and 15 neurotypical (NT), underwent a saccadic eye movement task. I conducted conventional analysis (movement duration, precision, velocity, RT) and Bayesian analysis. Saccade duration and velocity failed to differentiate the two groups, whereas amplitude was higher in ADHD than in NT participants. Saccade RT and variability were higher in ADHD than in NT participants. In the Bayesian analysis, ADHD altered the main distribution of saccades and of early saccades but did not influence the express saccade triggering. ADHD disrupts two mechanisms of action: it reduces the gain of the decision signal, thus explaining slowness and variability. It also fastens the decision process of early responses at the origin of short-latency but controlled movements. These premises and their interconnections explain previous observations and support the striatal-frontal wiring of ADHD, thus explaining ADHD complexity.

Attention deficit and hyperactivity disorder disrupts selective mechanisms of action as revealed by eye movements

Olivier A. Coubard
The Neuropsychological Laboratory CNS-Fed; olivier.coubard@cns-fed.com

People with attention deficit and hyperactivity disorder (ADHD) present attentional and emotional deficits and show paradoxical qualities such as hyperfocus. Using eye movements, previous studies have reported errors, slowness, and reaction time (RT) variability. The goal of this study was to explore the underlying mechanisms of ADHD further. Thirty French children and teenagers, 15 with ADHD and 15 neurotypical (NT), underwent a saccadic eye movement task. I conducted conventional analysis (movement duration, precision, velocity, RT) and Bayesian analysis. Saccade duration and velocity failed to differentiate the two groups, whereas amplitude was higher in ADHD than in NT participants. Saccade RT and variability were higher in ADHD than in NT participants. In the Bayesian analysis, ADHD altered the main distribution of saccades and of early saccades but did not influence the express saccade triggering. ADHD disrupts two mechanisms of action: it reduces the gain of the decision signal, thus explaining slowness and variability. It also fastens the decision process of early responses at the origin of short-latency but controlled movements. These premises and their interconnections explain previous observations and support the striatal-frontal wiring of ADHD, thus explaining ADHD complexity.

Identifying an eye movement phenotype for major depressive disorder

Blake K Noyes[1], Linda Booij[2], Heidi C Riek[1], Isabell C Pitigoi[1], Jeff Huang[1], Don C Brien[1], Brian C Coe[1], Brian J White[1], Sarosh Khalid-Khan[1], Douglas P Munoz[1]
[1]Centre for Neuroscience Studies, Queen's University, Canada; [2]Department of Psychiatry, McGill University, Canada; blake.noyes@queensu.ca

Eye-tracking can provide important insight into the impact of psychiatric disorders (e.g., major depressive disorder; MDD) on cognitive control, arousal, attention, and orienting responses. The goal of this study is to characterize saccade behaviour, pupil responses, and blink rate in adolescents with MDD, and compare to the behaviour of healthy control participants. The current sample includes 85 adolescent controls (mean age [M] 16.8 years, Patient Health Questionnaire [PHQ; measures level of depression] score=2) recruited from the community and 26 adolescents with MDD (M=15.9 years, PHQ=14.3) recruited from a local psychiatric outpatient program. All participants completed self-report mental health questionnaires and the Interleaved Pro-Anti Saccade Task (IPAST). Preliminary analyses showed that adolescents with MDD generated more direction errors on anti-saccade trials, blinked more during presentation of important visual instructions, and had blunted pupil responses throughout the task compared to control participants. These preliminary results indicate a step towards identifying the eye movement phenotype of MDD, and understanding how MDD impacts oculomotor circuits in the brain to produce altered behaviour.

Cross-task patterns of saccade abnormality in Parkinson's disease suggest multiple affected oculomotor processes

Heidi C Riek[1], Donald C Brien[1], Brian J White[1], Brian C Coe[1], David A Grimes[2], Anthony E Lang[3], Connie Marras[3], Mario Masellis[4], Douglas P Munoz[1]

[1]Queen's University, Kingston, ON, Canada; [2]The Ottawa Hospital, Ottawa, ON, Canada; [3]University Health Network, Toronto, ON, Canada; [4]Sunnybrook Research Institute, Toronto, ON, Canada; heidi.riek@queensu.ca

Saccade behaviour is underpinned by well-established circuitry spanning diffuse brain regions. Therefore, patterns of saccade alteration caused by Parkinson's disease (PD) can provide insight into inter-individual and longitudinal variation in pathology location and severity. These patterns can be measured using video-based eye tracking; 119 PD patients and 104 age-matched healthy controls completed an interleaved pro- and anti-saccade task (IPAST) and instruction-free viewing of rapidly changing video clips (FV). We compared behaviour between groups and across tasks to determine the effects of pathology and explore underlying neural mechanisms. PD demonstrated voluntary saccade control deficits (e.g., increased antisaccade errors, reduced FV voluntary saccade rate), indicating heightened inhibitory basal ganglia output. PD made more correct express prosaccades (90-140ms latency) and faster prosaccades overall, but these parameters did not correlate with voluntary control deficits, suggesting a different causal process (e.g., mechanism compensating for impaired movement initiation by enhancing responses to visual stimuli). Finally, saccade amplitude was reduced across tasks but was uncorrelated with abovementioned measures, suggesting a third dysfunctional mechanism. Together, these results illuminate three independent PD-affected neural processes that can be indexed by eye tracking, which may have implications for the development of screening or diagnostic tools.

Identifying early-stage Parkinson's Disease from Eye Movements During Steady State Fixation

Yiting Wang[1], Panagiota Tsitsi[1,2], Ioanna Markaki[1,2], Per Svenningsson[1,2,3], Gustaf Öqvist Seimyr[1], Mattias Nilsson[1]

[1]Karolinska Institutet, Department of Clinical Neuroscience, Sweden; [2]Center of Neurology, Academic Specialist Center, Sweden; [3]Karolinska University Hospital, Neurology Department, Sweden
yiting.wang@ki.se

Parkinson's disease (PD) is a progressive neurodegenerative condition affecting patients' motor systems, causing severe disability over time. Early diagnosis is crucial to improve quality of life through timely interventions. Ocular motor abnormalities in early PD have sparked interest in using eye movements for early detection. While predictive machine learning models on PD have focused on certain eye movements such as regular saccades and blink rate, fixational eye movements have been overlooked. However, these small involuntary movements can be easily captured through simple fixation tasks, potentially offering an easy and objective means to screen PD. This study explores whether microsaccades can indicate early-stage PD using data from a fixation task including 50 early-stage patients with unilateral to mild-to-moderate Parkinson's disease and 43 age-matched healthy controls. A Support Vector Machine (SVM) is trained to predict whether a microsaccade is more likely from a PD patient or a healthy control based on its spatial and temporal features. By aggregating the prediction results at the microsaccade level using majority voting, we are able to show a markedly improved classification performance at the trial and subject level. Results show 57.5% accuracy at the microsaccade level, 61.4% at the trial level, and 72.1% at the subject level, underscoring the relevance of microsaccade dynamics for early-stage PD screening.

Tuesday - afternoon - Eye Movements in the Study of Language - Venue C

Leveraging the Visual World Eye-Tracking Paradigm in Analyzing Second Language Pronoun Processing

Linghui Diao, Leah Roberts
University of York, United Kingdom
linghui.diao@york.ac.uk

Pronoun comprehension can be challenging for second language (L2) speakers, especially in contexts where the referent for the pronoun is ambiguous, for instance, where a pronoun can grammatically refer to either of two potential antecedents in the preceding discourse. However, few studies have investigated ambiguous pronoun resolution with L2 speakers using the visual world eye-tracking technique to observe how participants retrieve antecedents from working memory and identify locations of interest visually. Against this background, this study investigated subject pronouns in English by 50 Mandarin Chinese speakers learning English, comparing with 50 native English controls, on Gorilla Experiment Builder to analyze eye movement patterns during pronoun processing. Participants first listened to 24 sets of sentences while viewing corresponding displays where two possible antecedents of pronouns and a distracted item/launch pad are introduced. The results revealed that when there were two possible referents with same gender in the discourse, L2 English speakers, compared to L1 speakers, behaved differently in determining the resolution of pronouns. In unambiguous discourse contexts, L2 speakers showed a preference for shifting the topic, reflecting the influence of their first language. These results enhance our understanding of L2 pronoun processing and acquisition, offering insights into second language learning theories.

A transposed-syllable effect in Chinese spoken word recognition

Haibin Han[1], Danni Fan[1], Xingshan Li2,[3]
[1]Hebei Normal University, China; [2]Department of Psychology, University of Chinese Academy of Sciences, Beijing, China; [3]Key Laboratory of Behavioral Science, Institute of Psychology, Chinese Academy of Sciences
haibinhan@outlook.com

Early models of spoken word recognition assumed that words are represented as sequences of phonemes which activated based on linear positional matching during the temporal unfolding of the spoken word, as seen in models like the COHORT or TRACE model. However, recent evidence suggests a more flexible phonetic encoding, as research has shown that words can also be activated by transposing the order of phonemes. Since Chinese words are represented as syllables and the spelling-sound relationship in Chinese is not transparent, our experiments aimed to investigate whether the transposed-syllable effect exists in Chinese spoken word recognition. Using the printed-word paradigm, we found that listeners showed more fixations on target words (e.g., "冰锥/bing1 zhui1/, meaning *ice cones*") than on anadromes of the spoken words (e.g., "追兵/zhui1 bing1/, meaning *pursuers*") and distractors in two experiments. Most importantly, fixation probability on anadromes was significantly higher than on distractors, indicating a transposed-syllable effect. Moreover, the frequency of the spoken words and target words were also under controlled, revealing that word frequency moderates this transposed-syllable effect. These results provide further evidence for the flexible and position-independent phonetic encoding during spoken word recognition, as postulated in the TISK model.

Shifts in attention during listening of a novel: Evidence from eye tracking

Johanna Karoliina Kaakinen[1], Diane Mézière[1], Jonathan Smallwood[2], Karin Kukkonen[3], Jaana Simola[4]
[1]University of Turku, Finland; [2]Queen's University, Ontario, Canada; [3]University of Oslo, Norway; [4]University of Helsinki, Finland
johkaa@utu.fi

The present study examined how different types of thought patterns detected during listening of an audiobook are reflected in eye movements. Participants (N=58) were asked to focus their eye gaze on a fixation target while listening to an audio recording of a novel. Participants' eye movements were recorded, and they responded to a 13-item mind-wandering questionnaire 30 times during the listening task. A principal components analysis (PCA) of the mind-wandering questionnaire responses produced three components, which were labelled as Immersion, Mind-wandering, and Verbalizing. We then examined how these three thought patterns captured by the component scores extracted from the PCA were reflected in six outcome variables computed from the eye-tracking data: fixation duration, number of fixations, fixation dispersion, saccade amplitude, blink duration, and number of blinks. The results showed that Immersion was characterized by fewer and less dispersed fixations on the screen, shorter saccades, and longer blinks. Mind-wandering was related to more dispersed fixations. Verbalization resulted in more fixations, higher dispersion across the screen, and more blinks. The results suggest that eye movements reflect shifts in attentional focus during an auditory task that does not require visual processing. The results provide important information about the processes underlying literary experience.

Form-meaning iconicity facilitates semantic recognition of onomatopoeic words: Evidence from eye movements

Oksana Kanerva[1,2], Tuomo Häikiö[2]
[1]University of Helsinki, Finland; [2]University of Turku, Finland
oksana.kanerva@helsinki.fi

Iconicity refers to the one-on-one correspondence between form and meaning of words. It is considered to be instrumental in establishing connection between linguistic units and sensorimotor experience. Onomatopoeic words (e.g., Rus. *bac* 'bang') are known to depict various environmental sounds and indicate action connected to these sounds. This study investigated the sensitivity of adult Finnish L1 speakers with no prior knowledge of Russian to the iconicity of 50 Russian onomatopoeic words perceived by ear. First, an iconicity rating test was used to establish the iconicity levels for each token from the perspective of Finnish L1 speakers (N=28) who had never learned Russian before. Second, an eye-tracking experiment employing the visual world paradigm was used to examine visual recognition of the meaning of spoken onomatopoeic words. For this, we tested a different group (N=27) of native Finnish participants unfamiliar with Russian using the same tokens. Our results revealed that: 1) iconicity ratings varied among the tested onomatopoeic words suggesting that different tokens are unequally successful in mapping form and meaning; 2) higher iconicity substantially facilitated visual recognition of the semantics of stimulus words perceived by ear. In conclusion, form-meaning iconicity underpins semantic accessibility of unknown words.

Where do children look when watching videos with same-language subtitles?

Anastasiya Lopukhina[1], Walter van Heuven[2], Rebecca Crowley[1], Kathleen Rastle[1]
[1]Royal Holloway, University of London, United Kingdom; [2]University of Nottingham, United Kingdom
nastya.lopukhina@gmail.com

Influential campaigns in the UK and USA have argued that turning on same-language television subtitles could dramatically improve children's reading outcomes (https://turnonthesubtitles.org/). However, it is unclear whether children even look at subtitles. We aimed to determine whether primary-school children pay attention to and read same-language subtitles. We tested 180 British children from Years 1-6 (age range 5-11). Each participant watched two videos with and two without subtitles, while their eyes were tracked with the Eyelink Portable Duo eye-tracker. We measured children's reading fluency using the TOWRE. For the analysis, we divided the screen into the video and the subtitle regions. All children looked at the subtitle region more often when the subtitles were present. Crucially, superior readers were more likely to look at the subtitles than less proficient readers and spent more time attending to them. Fixations on words in the subtitles indicated their linguistic processing. We concluded that some degree of reading fluency is necessary before children pay attention to subtitles. Poor readers tend to not look at the subtitles, suggesting that children will not learn to read from subtitles. We conclude by discussing some differences between reading subtitles and reading text that motivate further research.

Pupillary responses during extended listening in L2 learners of English

Yuhan Luo, Dagmar Divjak, Petar Milin
University of Birmingham, United Kingdom
YXL1547@student.bham.ac.uk

Pupillometry's role in measuring listening effort is well-documented in second language (L2) learning research (Schmidtke, 2018), yet its efficacy in extended listening tasks is less understood. This study explores pupillary responses of 80 native Chinese learners of English engaged in listen-to-summarize tasks. This task started with a 1-1.5 minute lecture, followed by a summary writing of the lecture. Through analysing their responses, we aim to shed light on the nature of listening effort as a physiological indicator and its relationship with L2 task performance.

Data were analysed using Generalized Additive Mixed-Effect Modelling (GAMM) for time-series analysis and Spearman correlation to link pupillary response with performance. We observed a nonlinear pattern of listening efforts, with a significant initial peak in pupil dilation and smaller peaks later, indicating variable engagement as the listening task unfolds. In other words, the nonlinear trend suggests a high initial cognitive effort, with subsequent phases of lesser engagement. In addition to this, a moderately significant correlation (rho=0.45) between pupil dilation and performance suggests that increased listening effort correlates with better task outcomes.

This research highlights pupillometry's value in assessing listening effort and cognitive engagement in L2 learning, establishing a clear connection between physiological responses and language performance.

Wednesday - morning - Reading Development - Venue A

Effect of interruptions during reading on comprehension and eye movements in children

Guillaume Chevet[1], Saoussane Ouahmiden[1], Thierry Baccino[2], Annie Vinter[1], Xavier Aparicio[3], Véronique Drai-Zerbib[1]
[1]University of burgundy, France; [2]University Paris 8, France; [3]Laboratoire CHArt, Université Paris-Est Créteil
guillaume.chevet@hotmail.fr

We conducted a study to evaluate the impact of interruptions during reading in children in 2nd and 5th grade. Children were asked to read an age-appropriate text on a computer screen while their eye movements were recorded. They took part in two conditions: interrupted reading and uninterrupted reading. In the interrupted condition, the interruption occurred when the gaze entered into a visual trigger area. The interruption was presented auditory: the children had to listen to a short lesson and then answer a question about what they had just heard before they could resume reading. Surprisingly, there was no decline in comprehension performance in the interrupted condition. However, there were more markers of rereading (more fixations and longer fixation durations for 2nd pass reading) than in the uninterrupted condition in the part of the text before the interruption as well as in the interrupted sentence itself. Interestingly, this was more pronounced in 2nd graders than in 5th graders. These data suggest that children employ a rereading strategy adapted to their reading competence in order to achieve and maintain good text comprehension.

Eye movements when reading pseudo-text in dyslexic children: Evidence from eye tracking

Nicol Dostálová, Roman Švaříček
Masaryk University, Czech Republic
nicol.dostalova@mail.muni.cz

Dyslexia is a specific learning disorder that is manifested by difficulties with reading, which is evident also in the gaze performance. This paper aims to present the results of a study that focused on the detection and analysis of specifically selected parameters of eye movements (fixations and saccades), in child dyslexic readers during the pseudo-text reading, as a part of diagnostic batteries in the Czech Republic. For these purposes, 70 participants (35 dyslexic, 35 intact) of elementary school readers in the age range of 9-10 years were measured, including eye movement detection during the performance of pseudo-text reading. Data analysis focused on parameters of fixations and saccades (i.e. fixation duration, number of fixations, saccadic amplitude, and number of regressions). These metrics were analyzed at the level of the text as a unit, individual sentences, and separate words. Results show that dyslexic readers exhibit a higher number of fixations as well as a longer fixation duration compared to intact readers. Dyslexics also perform a higher number of regressions and shorter saccadic amplitude. These results may provide new insights into the diagnostics of dyslexia, which can be followed up with precisely targeted intervention to support further development of the dyslexic reader.

Decoding Gender in German: Insights from Eye-Tracking Experiments on Language Comprehension in Children with Developmental Language Delay

Thomas Günther[1], Annika Kirschenkern[2], Frederike Steinke[1], Jürgen Cholewa[3]
[1]Department of Psychological Assessment and Intervention, Institute of Psychology, RWTH Aachen University; [2]Child Neuropsychology Section, Department of Child and Adolescent Psychiatry, Psychotherapy and Psychosomatics, Medical Faculty, RWTH Aachen University; [3]Institute of Special Education, Heidelberg University of Education, Heidelberg
guenther@psych.rwth-aachen.de

In contrast to English, German incorporates a gender system. Our eye-tracking experiments highlight the significant impact of external gender cues, like "der [masculine] Hund" (the dog) versus "die [feminine] Katze" (the cat), enhancing language comprehension via predictive processing (Cholewa et al., 2019; Bürsgens et al., 2021). Investigations show ongoing development in predictive processing for grammatical gender during primary school years (Günther et al., 2023).
Our subsequent inquiry delves into how children with developmental language delay (DLD) utilize gender information in language comprehension. Analyzing eye movements in a language–picture matching task involving 26 control children and 28 with DLD (aged 6–10), the study aims to identify DLD-specific differences in gender cue processing during both slow and normal speech rates.
Results reveal that, under normal speech conditions, children with language development disorders don't benefit from external gender cues. However, at a slower pace, they demonstrate the ability to use gender cues for predictive noun comprehension. Findings suggest that challenges in employing gender information for language comprehension in children with DLD stem from limitations in language processing speed, despite their expressive correctness in using gender.

Predicting young children's eye movements during reading from teachers' ratings of inattention

Christopher J. Lonigan[1], Ralph Radach, Christian Vorstius
[1]Florida State University, [2]University of Wuppertal

Attention problems are significantly associated with children's reading skills. Higher levels of inattention are associated with lower scores on most measures of reading, and there is substantial overlap between reading disabilities and ADHD. The aim of this study was to determine how children's inattention affected their moment-by-moment reading, including problems with more difficult text, compared to measures of overall reading skill. The sample included 345 third- to fifth-grade children from Florida schools. Eye-movements were recorded using an EyeLink1000 while children read 48 declarative sentences in both silent- and oral-reading conditions. Target words (all nouns) varied in character length and lexical frequency. Children completed a large battery of standardized psychoeducational measures, including word-decoding and reading comprehension, and teachers rated children's inattentive behaviors. Multilevel analyses revealed main effects of word-length, word-frequency, and decoding skills or reading comprehension skills on fixation, refixation, rereading times for both reading silently and aloud. Inattention was sometimes a main effect. The effects of word characteristics were sometimes moderated by decoding and reading comprehension but rarely by inattention, with effects primarily on refixation duration. These results indicate that although children's inattention contributes to lower reading performance, it is primarily a general effect not influenced by text characteristics.

Characterising Children's Eye Movement Control During Reading in English: A Corpus Study

Sara V. Milledge[1], Chuanli Zang[1], Hazel I. Blythe[2], Simon P. Liversedge[1]
[1]University of Central Lancashire; [2]Northumbria University
smilledge@uclan.ac.uk

Past research examining English child readers' eye movement behaviour has primarily compared when, rather than where, the eyes move relative to skilled adult readers. We used an English eye movement corpus to characterise where typically developing 8- to 9-year-old child readers target their eyes during silent sentence reading. We assessed differences in initial landing position (ILP) dependent on word length, launch site, reading skill, and frequency. The results show that children's saccadic targeting is not interactively constrained by launch site and word length in an adult-like manner. Reading skill interacted with launch site: as reading skill increases, ILPs are further into words at closer launch sites and for far launch sites, ILPs are less far into words. Bayesian analyses indicate that this interactive pattern is also present in 8- to 9-year-old child readers. Also, when word length and launch site are controlled, word frequency affects the ILP: as word frequency increases, readers land further into a given word. Overall, the results suggest that there is developmental change in the saccadic targeting system, a change for which no model of eye movement control during reading can account. The effect of reading skill also suggests a role for cognitive processing.

Reading Efficiency, Academic Performance, and Visual Comfort: Exploring a Hierarchical Model

Alexandra Spichtig[1], John Ferrara[1], Nancy Brower[1], Kristin Gehsmann[2]
[1]Stanford Taylor Foundation, United States of America; [2]Clemson University, United States;
alex@stfvision.org

Four indicators of comprehension-based silent reading efficiency (rate, fixations, regressions, and fixation duration) were measured in U.S. students in grades 2-8 (n=99) and compared to students' academic measures (STAR Reading and Missouri Assessment of Progress (MAP) English and Math scores) and visual comfort. Reading efficiency data were obtained by an eye-movement recording system used in previously published studies, while visual comfort data were obtained by a researcher-developed visual skill questionnaire addressing four domains of visual skill proficiency (Binocular Fusion/Eye Teaming, Tracking/Eye-Movement Control, Eye Focusing & Accommodation, and Secondary Symptoms/Behavioral).

All reading efficiency measures showed significant correlations with academic measures ($p<0.001$; Pearson's r ranging between -.380 to .590 with MAP measures and -.720 to .790 with STAR scores). Binocular Fusion/Eye Teaming and Tracking/Eye Movement Control were significantly correlated with reading rate, fixations, fixation duration, and the academic measures. Secondary Symptoms/Behavioral and Eye Focusing/Accommodation were only correlated with the STAR measure. Additional analyses by grade groups (e.g. 2-3, 4-5, 6-8) showed potential developmental implications, and multiple regressions revealed that only some correlations had predictive qualities for some grade groups, which needs to be considered in understanding students' instructional needs.

Wednesday - morning - Eye Movements in Social Interaction - Venue B

Mutual gaze in social interactions: The effects of collaboration and competition

Florence Mayrand, Jelena Ristic
McGill University, Canada; florence.mayrand@mail.mcgill.ca

Shared eye gaze is a fundamental aspect of human social communication. Past work has shown that, contrary to a common intuition, humans spend little time engaged in eye-to-eye mutual looks during naturalistic interactions. Here to address how the nature of the task impacts this result, we used dual mobile eye tracking eye glasses to record gaze patterns of interacting dyads while they performed a survival task. Critically, some groups were asked to collaborate to solve the task together while the others were asked to compete to solve the task individually. We measured the amount mutual gaze as a function of whether participants looked at the upper half of the face (the eye region) or the lower half (the mouth region) of their partner. Preliminary data show that overall, the amount of time spent in mutual gaze remained low during the interaction, on par with previous studies. However, dyads who competed to solve the task engaged in less mutual looks overall compared to dyads who collaborated, specifically in the Eye-to-Mouth combination. Thus, while these results once again show that mutual looks are infrequent during natural interactions, looking patterns appear to be influenced by the social nature of an interactive task.

A setup for the cross-cultural study of gaze behavior and eye contact in face-to-face collaboration

Roy Hessels[1], Toshiki Iwabuchi[2], Diederick Niehorster[3,4], Ren Funawatari[2], Jeroen Benjamins[1,5], Sayaka Kawakami[2], Marcus Nyström[3], Momoka Suda[2], Ignace Hooge[1], Motofumi Sumiya[2], Julie Heijnen[1], Martin Teunisse[1], Atsushi Senju[2]
[1]Experimental Psychology, Helmholtz Institute, Utrecht University, Utrecht, the Netherlands; [2]Research Center for Child Mental Development, Hamamatsu University School of Medicine, Hamamatsu, Japan; [3]Lund University Humanities Lab, Lund University, Lund, Sweden; [4]Department of Psychology, Lund University, Lund, Sweden; [5]Social, Health and Organisational Psychology, Utrecht University, Utrecht, The Netherlands
r.s.hessels@uu.nl

Gaze avoidance during interaction is suggested to be characteristic of East-Asian compared to Western-European cultures. However, most reports are anecdotal, or based on annotation from videos or eye-tracking studies with images of faces. Few cross-cultural studies on eye contact in interaction were conducted using eye-trackers. We developed a setup for the cross-cultural study of gaze behavior in interaction, with automated eye-tracking data analysis. Japanese and Dutch dyads participated in conversation, mutual collaboration, and instructor-instructee collaboration. Gaze behavior was recorded with the Tobii Glasses 3 (TG3, Japan) and the Pupil Invisible (PI, the Netherlands) wearable eye-trackers. A top-view camera filmed the dyad and scene. Fiducial marker technology and face-detection software were used for AOI analyses. Setup validation: Data collection was technically successful for 21/24 Japanese and 24/30 Dutch dyads. Eye-tracking data quality was sufficient for accurate AOI assignment. Mean accuracy, precision and data loss were respectively 2º, 0.5º and 12% for the TG3, and 4º, 0.2º and 0% for the PI. The synchronization of eye-tracking data to top-view video was accurate to 33±15 ms.Our setup allows the investigation of context-specific and context-generic differences in eye contact and gaze behavior between cultures in a more objective manner than previous research.

Dual mobile eye-tracking during social interactions to predict problem-solving performance & decision-making behaviour

Alexandra Hoffmann
Leopold-Franzens-Universität Innsbruck, Austria
alexandra.hoffmann@uibk.ac.at

In my talk I would like to present two studies, where we applied dual mobile eye-tracking during social interactions to predict (1) problem-solving performance and (2) decision-making behavior. In the first study, we tracked the snychronization of blink rate between two individuals working virtually together (N = 38) to predict their performance in a problem-solving team task. In the second study, we tracked mutual eye-contact during 240 speed dating interactions (60 subjects; 4 speed dates each) and predicted indiviual's mate choice while controlling for perceived attractiveness. After presenting design and results of those two studies, I would talk about the advantages and challenges of dual mobile eye-tracking during social interactions.

Semantics of gaze in person perception: A novel qualitative-quantitative approach

Lynn Huestegge, Eva Landmann, Christina Breil, Anne Boeckler
University of Würzburg, Germany
lynn.huestegge@uni-wuerzburg.de

Interpreting gaze is essential in social interactions. Surprisingly, research on gaze semantics is rather limited and often shaped by strong (probably unwarranted) theoretical preconceptions. We investigated effects of different gaze behavior patterns on gaze perception using a two-step, qualitative-quantitative approach. Participants watched videos of single persons listening to narrations by another (invisible) person. The listener’s gaze behavior was systematically manipulated in terms of gaze direction, frequency and direction of gaze shifts, and blink frequency; emotional context was manipulated by presenting neutral vs. negative narrations. In Experiment 1 (qualitative-exploratory), participants freely described which states/traits they attributed to the listener across conditions, allowing us to identify relevant aspects of person perception in a bottom-up manner and to construct rating scales that were later implemented in Experiment 2 (quantitative-confirmatory). Overall, results revealed systematic meanings ascribed to the listener’s gaze behavior. For example, rapid blinking and fast gaze shifts were rated more negatively (e.g., as restless/unnatural) than slower gaze behavior; downward gaze was evaluated more favorably (e.g., as empathetic) than other gaze aversion types (e.g., sideways or upwards), particularly in an emotionally negative context. Overall, our study represents a first step towards a more systematic (and less theoretically pre-determined) understanding of social gaze semantics.

Eye Contact Modulates Eyeblink Synchronization and Rapport

Mehtap Cakir, Anke Huckauf
Ulm University, Germany
mehtap.cakir@uni-ulm.de

Eye contact is a rich social and affective cue that can ease communicational and collaborative tasks and enable perceptions of others' traits and mental states. However, understanding how gaze behavior and moment-to-moment feedback during eye contact modulate behavior and emotions, as well as the role of eyeblinks in this modulation, poses ongoing challenges. In our research, the interactive gaze behavior of pairs is to be examined in real-world dual eye-tracking setups. In our investigation, we analyzed the synchronization of eyeblinks during interactions distinguished by eye contact, devoid of task-related or conversational components that can elicit similarities in visual, auditory, or cognitive processing. We isolated the role of gaze in three conditions: face-to-face eye contact, mediated eye contact through a mirror, and self-directed gaze in a mirror. The results revealed that during interactions facilitated by direct eye contact, there was a concurrent increase in eyeblink synchronization, along with a heightened sense of rapport between the pairs. Even minor deviations resulting from mediated interaction led to decreased synchronization and a diminished sense of connection among partners. The findings propose that eyeblink synchronization can serve as an indicator of rapport, and provide the potential of grasping authenticity of human-robot interaction.

Infant action prediction and gaze behavior in interaction with their parents

Niilo V. Valtakari, Ignace T.C. Hooge, Chantal Kemner, Roy S. Hessels
Experimental Psychology, Helmholtz Institute, Utrecht University, Utrecht, The Netherlands
niilovaltakari@gmail.com

Considering that most actions infants observe are performed by their parents in face-to-face contexts, one might expect parents to play a role in the development of action prediction. Despite this, previous research has focused on infants observing video recordings of actions performed by strangers. We recruited 41 parent-infant dyads and had each parent perform a set of functional and nonfunctional actions in front of their infant. Gaze and video data from parent and infant was recorded using a dual eye-tracking setup. We outline four distinct theoretical models that may explain infants' anticipatory gaze behavior. Infants' anticipatory gaze behavior was best described by a repetition-based prediction model. However, a bias to look at the mouth was also observed. Following, we quantified parents' nonverbal behaviors while they performed the actions and report that some aspects of parents' nonverbal behaviors were related to the anticipatory gaze behavior of the infants. We conclude that in face-to-face interaction, disentangling biases in gaze behavior from action prediction is not trivial. Regardless, parents provide infants with nonverbal cues that may guide their gaze to the targets of actions. Providing such cues may foster the development of action understanding.

Wednesday - morning - Visual Processing and Cognitive Control - Venue C

Understanding behavioral regulation: How the eyes reveal exploration-exploitation dynamics

Giovanna C Del Sordo[1], Megan H Papesh[2]
[1]New Mexico State University, United States of America; [2]University of Massachusetts Lowell, United States of America
delsordo@nmsu.edu

The exploration-exploitation tradeoff is a key element of Adaptive Gain Theory, suggested to promote the optimization of behavior toward high performance. This tradeoff is closely related to the phasic and tonic modes of the locus coeruleus-norepinephrine (LC-NE) system. Pupillometry has been shown to serve as a valuable tool to monitor shifts in exploration and exploitation control states. To measure exploration and exploitation using a paradigm with tonic (baseline) and phasic (task-evoked pupil responses) recordings, the present study used a novel Complex Card Sorting task. Participants learnt complex classification rules through trial and error using correct-incorrect feedback across varying difficulty levels. This task tapped into cognitive flexibility, as participants alternated between exploration trials (i.e., testing new rules) and exploitation trials (i.e., exploiting the rule that currently applies). The present study measured the relationship between LC-NE functioning modes and the adaptive regulation of cognitive control through pupillometry. This allowed us to confirm the role of the LC-NE's adaptive regulation in optimized behavioral performance. Furthermore, we examined the impact of task difficulty on behavioral performance and tonic/phasic LC activity.

Deceptive Intentions: Insights from Eye Movements in a Card Game Experiment

Valentin Foucher, Anke Huckauf
Ulm University, Germany
valentin.foucher@uni-ulm.de

The potential of eyes to unveil cognitive processes is widely acknowledged. However, comprehending the intricate relationship between eyes and deceptive intentions is challenged by the eyes' susceptibility to external and internal factors. To address this, our study delved into the role of fixations and pupil motion as markers of deceptive intentions within the context of a card game. Participants were instructed to select a number from 1 to 6 on a card and were then presented with all card options either simultaneously or sequentially on a screen. We examined two levels of deceptive intentions: concealing or faking the chosen number, and compared them to an honest control condition. Our findings revealed distinct patterns: concealing intentions were associated with increased and shorter fixations when multiple cards were displayed, and decreased and longer fixations when only one card was shown. Moreover, while fixations identified the cards participants intended to disclose in both honest and faking conditions, pupil size further distinguished the hidden chosen card in both concealing and faking conditions. Our results underscore the importance of differentiating at least two kinds of deceptive intentions from eye movements, while also exposing how the number of objects in the design influences deceptive eye behaviours.

Deciphering episodic memory encoding and retrieval: a glimpse through eye movements and EEG

Andrey R. Nikolaev, Roger Johansson, Inês Bramão, Mikael Johansson
Lund University, Sweden
andrey.nikolaev@psy.lu.se

Eye movements support episodic memory encoding and retrieval processes. During encoding, they actively link spatiotemporal features, thus contributing to the formation of coherent episodic memories. During retrieval, the reinstatement of eye movements from encoding facilitates the reactivation of the corresponding memories. Despite the importance of these interactions, little is known about the neural mechanisms subserving episodic memory through eye movements. Here, we utilize concurrent EEG and eye-tracking to examine episodic memory in a free viewing task. Participants first encoded a series of multi-element events. In a subsequent forced-choice memory test, participants retrieved associations specifying each event, selecting a target element related to the cue against a distractor from another event. Compatible with prior research, subsequent memory performance increased with the cumulative number of gaze transitions between elements during encoding. During retrieval, gaze was disproportionately allocated to target elements. Fixation-related EEG analyses during encoding showed that theta activity following between-element gaze transitions predicted subsequent memory. Theta and alpha activity corresponding to different fixation intervals within a test trial predicted memory performance during retrieval. Collectively, these findings suggest that the interplay between fixation-related theta and alpha activity supports the encoding and retrieval of episodic memories across eye movements.

Covert Attention Leads to Fast and Accurate Decision-Making

Sonja Perkovic, Martin Schoemann, Carl-Johan Lagerkvist, Jacob L Orquin
Aarhus University, Denmark
jalo@mgmt.au.dk

Decision-makers are regularly faced with more choice information than they can directly gaze at in a limited amount of time. Many theories assume that because decision-makers attend to information sequentially and overtly, that is, with direct gaze, they must respond to information overload by trading off between speed and decision accuracy. By reanalyzing five published studies, we show that participants, besides using overt attention, also use covert attention. That is, without being instructed to do so, participants attend to information without direct gaze to evaluate choice attributes that lead them to either choose the best or reject the worst option. We show that the use of covert attention is common for most participants and more so when information is easily identifiable in the peripheral visual field due to being large or visually salient. Covert attention is associated with faster decision times suggesting that participants might process multiple pieces of information simultaneously using distributed attention. Our findings highlight the importance of covert attention in decision-making and show how decision-makers may be gaining speed while retaining high levels of decision accuracy. We discuss how harnessing covert attention can benefit consumer decision-making of healthy and sustainable products.

Using Context to Improve Prospective Memory: A Pupillometry Study

Megan H Papesh[1], Giovanna C Del Sordo[2], Madison Rowley[1]
[1]University of Massachusetts Lowell, United States of America; [2]New Mexico State University
megan_papesh@uml.edu

How do you remember to stop at the pharmacy on your way home from work? Accomplishing this sort of prospective memory (PM) task can be done via both overt reminders (e.g., an alarm at the end of the day) and awareness of contextual cues (e.g., "spontaneous" retrieval when you pass the pharmacy). Prior work has shown that contextual expectations can change attention allocation during laboratory PM tasks: While participants completed an ongoing short-term memory color-matching task, they monitored for PM targets with varying levels of contextual expectations. When expectations were high, they slowed ongoing performance and incidentally remembered more from the task, despite that information being task irrelevant. In the present study, we recorded pupil diameter as a measure of attentional arousal to determine when contextual expectations heighten attentional states. Behavioral results replicate prior findings, showing that contextual expectations slow ongoing task performance and enhance incidental memories. By measuring pupil diameter during both the context recognition and expectation violation phases, we can determine how context influences both prospective memory and incidental memory formation.

Purposive engagement is crucial for eliciting oculomotor markers of effort

Christian Wolf[1], Michael B. Steinborn[2], Lynn Huestegge[2]
[1]University of Muenster, Germany; [2]University of Wuerzburg, Germany
chr.wolf@uni-muenster.de

Parameters of saccadic eye movements are known to reflect mental states like effort or fatigue. For example, peak-velocities are higher and latencies shorter when a person is mobilizing resources for speeded action. Here we examined the role of purposiveness in the effective mobilization of resources. Therefore, we used a saccadic selection task that requires inhibiting a distractor and selecting the correct target. Participants were either instructed to look at the center of the target as quickly as possible without making mistakes (standard instructions) or to mobilize all resources and respond even faster and more accurately ("*to give 110%*"; effort instructions). Each participant completed three blocks on different days. One block contained standard trials only (baseline). The other two blocks contained brief passages of 10 successive effort trials. In one of the two effort blocks, effort was rendered purposive by rewarding correct effort trials contingent on the response time. Results show that effort instructions reduced latencies, and increased amplitude and peak-velocities – but mainly when effort was rewarded. These findings cannot be attributed to changed behavior in standard trials. Altogether, the present evidence underscores that purposiveness is essential for action mobilization and should constitute a central element in theories of action.

The effect of the internal characteristics of coherence relations on their processing and comprehension

Andrea Carolina Santana Covarrubias
Pontificia Universidad Católica de Valparaíso, Chile
andrea.santana@pucv.cl

Coherence relations play a pivotal role in constructing a mental representation of the situation described in the text. The Cumulative Cognitive Complexity Hypothesis (CCCH) claims that coherence relations have different internal complexity, which implies differences in terms of acquisition (e.g., causal, and contrastive relations are acquired later than additive relations) (Evers-Vermeul & Sanders, 2009). This leads us to question whether the internal complexity of coherence relations also affects their processing and comprehension. This proposal aims to determine the effect of the internal characteristics of coherence relations on their processing and comprehension. To achieve this goal, an eye tracking experiment was conducted with 40 university students. We compared Total Fixation Duration, First and Second Pass Reading Times of segment 2 of coherence relations under two conditions: complex (*causal-negative-subjective-implicit*) and simple (*additive-positive-objective-explicit*). Data were collected using Portable Duo Eye Tracking equipment and analyzed using linear mixed-effects models (LMM) via the lme4 package in the R statistical software. These preliminary results revealed longer fixation times and more regressions in the complex condition, thus demonstrating consistency with the CCCH.

Reading Comprehension Tests and the Jingle Fallacy: Implications for Eye Movement Research

Charlotte Lee[1], Hazel I Blythe[2], Hayward J Godwin[1], Ascensión Pagán[3], Denis Drieghe[1]
[1]School of Psychology, University of Southampton, United Kingdom; [2]School of Psychology, Northumbria University, Newcastle, United Kingdom; [3]School of Psychology and Vision Sciences, University of Leicester, United Kingdom
charlotte.lee@soton.ac.uk

The *Jingle Fallacy* (Thorndike, 1904) is the false assumption that instruments that share the same name measure the same underlying construct. Over two large individual differences studies examining eye movements during reading, data from 88 and 91 participants respectively, revealed that the reading comprehension subtests of the Nelson Denny Reading Test (NDRT) and the Wechsler Individual Achievement Test (WIAT-II) did not load together in principal components analyses amongst other reading related skills. Reading comprehension tests currently used in eye tracking research are often inconsistent in predicting individual differences in skilled adult populations depending on which test is used. In our two eye tracking experiments, one examining word frequency effects during sentence reading, and another examining comprehension demands during paragraph reading, we also found that different patterns of eye movements were predicted by each reading comprehension test. Together these results indicate that these different comprehension measures should not be used interchangeably, and researchers should be cautious when selecting reading comprehension tests for research and comparing findings from studies using one of these tests with studies using the other.

Overall effects and item-level measures port well across eye-tracking and self-paced reading but participant-specific effects port poorly

Van Rynald T Liceralde[1], Tessa Warren[2], Ariel N James[3], Duane G Watson[1]
[1]Vanderbilt University, United States of America; [2]University of Pittsburgh, United States of America; [3]Macalester College, United States of America
vrtliceralde@gmail.com

Eye-tracking and self-paced reading (SPR) are commonly used to study sentence processing. Psycholinguists often assume that their outcomes are *portable*, i.e., that outcomes in one method directly map onto the other. We evaluate this assumption. Specifically, we ask how averages and effects port across methods overall and at the participant and item levels. 116+ participants read the same 120 English sentences in the same order in separate eye-tracking and SPR sessions that were 7+ days apart. Eighty sentences included a subject/object-extracted relative clause (RC) manipulation; 40 sentences included a lexical age-of-acquisition (AoA) manipulation. We examined the portability of lexical (word frequency, length, AoA) and syntactic (RC) effects. All expected fixed effects were present in both methods. Participant-specific and item-specific random intercepts correlated well, indicating that overall processing speed ports across methods. After adjusting for processing speed, item-level measures (e.g., adjusted gaze duration, RTs) also correlated well across methods, whereas participant-level measures did not. Moreover, participant-specific random effects correlated poorly across methods, with the RC effect porting more poorly than the lexical effects. These results suggest that although mapping overall effects and item-level measures across methods is warranted, researchers may need to be more careful about comparing people's performance across methods.

Eye movements from a 'read-only' task predict reading comprehension as measured by recall

Diane C. Mézière[1], Lili Yu[2], Titus von der Malsburg[3], Erik D. Reichle[2], Genevieve McArthur[4]
[1]University of Turku; [2]Macquarie University; [3]University of Stuttgart; [4]Australian Catholic University
diane.meziere@utu.fi

In recent years, studies have shown that eye-movement measures can predict performance on standardized reading comprehension measures. However, results suggest that eye movements are influenced by differences in task demands, such that no specific set of measures were useful across comprehension tasks. In this study, we investigate whether eye movements collected during a reading task with no additional demands (e.g., questions) can predict comprehension as measured by recall. 62 adults were given 9 passages to read while we tracked their eye movements. The passages were divided into three conditions: read-only, recall-1, and recall-2. The first 3 passages were read with no additional task, and the remaining 6 passages were followed by a recall task. Passages in the read-only and recall-1 conditions were counterbalanced. We used linear models and cross-validation to predict performance on the recall-2 task from eye movements in the read-only condition and the recall-1 condition separately. Results showed that eye-movement measures were useful in predicting recall in both conditions (R^2 = 0.26 and 0.27 respectively). Similarly, the most useful predictors of recall performance were nearly identical across conditions. These results demonstrate the usefulness of eye movements collected during reading with no additional task to predict comprehension.

Role of Empathy in Children's Processing and Comprehension of Written Irony

Henri Olkoniemi[1], Tuomo Häikiö[2], Matti Laine[3], Penny M. Pexman[4]
[1]University of Oulu, Finland; [2]University of Turku, Finland; [3]Åbo Akademi University, Finland; [4]Western University, Canada
henri.olkoniemi@oulu.fi

Verbal irony is a relatively late-developing skill. It involves understanding the opposite intention behind the literal meaning of a phrase. This is particularly challenging for children because many of the underlying skills needed to understand verbal irony are not fully developed. One of these skills is the ability to understand other people's feelings, which is an integral part of empathy. In this study, we investigated how children's level of empathy affects their processing and comprehension of written irony. We analysed data from two of our previous eye-tracking experiments with 10-year-old children (n = 97, 46 girls). In both experiments, children had read both ironic and literal sentences embedded in story contexts. After each story, they answered a text memory question and a comprehension question. In addition, their reading ability and empathy were assessed. The results showed that children with higher empathy skills showed better comprehension of irony and were less likely to reread ironic target sentences. These findings enhance our understanding of how the development of emotional skills influences language comprehension. They also have implications for current theories of figurative language comprehension that do not take into account developmental changes.

The effect of state curiosity on eye movements during reading of health-related arguments

Jaana Simola[1], Hanna Julku[1], Caitlin Dawson[1], Tuomo Häikiö[2]
[1]University of Helsinki, Finland; [2]University of Turku, Finland; jaana.simola@helsinki.fi

Curiosity is critical for learning and selective information processing. However, it is unclear how it affects eye-movement patterns during reading. We tracked participants' (n=49) eye movements in a task with 18 health-related questions. Each question was followed by maximum of 15 arguments from three argument quality categories: scientific relevant (SR), scientific irrelevant (SI), and non-scientific relevant (NR). These arguments were divided into three boxes based on the argument quality without the participants knowing the contents of the boxes. The participants could click on any box to reveal a new argument until they had read all the arguments or were ready to answer a multiple-choice question. After reading each argument, participants rated their level of state curiosity. Eye movements were analyzed at word-level controlling for the effects of word length and frequency. Curiosity did not affect first-pass reading. Total fixation duration was overall the longest for high quality (SR) arguments, with no effect of curiosity. However, total fixation durations were higher for low quality (SI and NR) arguments when curiosity was rated high. With increasing curiosity, low quality arguments were read more carefully, possibly helping readers to determine the credibility and usefulness of the information.

Gaze Behavior of Dentists Inspecting Bitewings with an AI-Support Tool: Implications for Expert Diagnostic Strategies and AI Usability

Nora Jane Castner
Carl Zeiss Vision International GmbH, Germany
nora.castner@zeiss.com

In medicine, human-AI collaboration can promote diagnostic performance. Expert gaze models can offer insight into how professionals adapt their behavior to AI. Moreover, improving interaction design of medical AI-systems improves understanding and trust. We summarize three articles addressing expert gaze behavior while interacting with a medical AI support system and investigate diagnostic visual strategies and system usability.
We combine two fields in eye tracking, expert gaze behavior during medical image examination and gaze for usability evaluation. Thus, we started with: How do experts scanpaths relate to bitewing inspection for caries detection? Then, how does an expert's scanpath deviate from their usual approach due to AI? Ultimately, bringing us to the question: Does AI-support potentially disrupt typical, expert scanpaths? Our methodology is detailed in the supplementary figure. We found systematic visual inspection style was the standard approach for dentists. However, with AI, systematic strategies became interrupted, but more attention was directed to relevant features (caries). Concerning usability, there were more transitions from the bitewing to the UI. This could have negative implications on workflows and fatigue but help design these systems. Ultimately, this knowledge of expert gaze features can aid developing proficient diagnostic workflows and seamless interaction with medical AI systems.

Children's eye gaze during a face-to-face conversation: Effects of social anxiety and conversational role?

Jiemiao Chen, Esther van den Bos, Michiel Westenberg
Institute of Psychology, Leiden University, the Netherlands
j.chen@fsw.leidenuniv.nl

Social anxiety (i.e. anxiety about negative evaluation and rejection) has long been associated with eye-contact avoidance. Supportive evidence is primarily based on adults and adolescents, whereas results on children are contradictory with studies reporting less, normal, or even *more* eye-contact. Further, those results are mainly based on observer coding of gaze behavior. Additionally, reduced eye gaze during speaking (compared to listening) was observed in adults, whereas it remains unclear whether children display the same pattern. Taken together, utilizing wearable eye-tracking technology, the present study aimed to elucidate how social anxiety is related to eye gaze in children during a face-to-face conversation. The effect of conversational role was examined. Approximately 60 children (aged 9 and 10 years) had a getting acquainted conversation with a young, female adult (i.e. confederate) for approximately 13 minutes. They alternated between the roles of listener and speaker, with their eye movements tracked by two pairs of eye-tracking glasses throughout the conversation. Social anxiety was measured using questionnaires. Fixation duration and fixation counts on the confederate's eye-region were extracted. In this presentation, we present the preliminary results of the study.

Hungry Eyes: Linking Food Cue Processing and Cognitive Glucose Sensitivity

Tobias Neukirchen, Ralph Radach, Christian Vorstius
University of Wuppertal, Germany
neukirchen@uni-wuppertal.de

Cognitive glucose sensitivity (CGS) measures an individual's reliance on external sources of glucose for cognitive functioning. Our previous research has shown that high CGS is associated with disadvantages in cognitive performance, weight-loss, and long-term blood sugar regulation. Compensating for cognitive deficits by ingesting glucose is a short-term regulatory strategy that is likely to increase CGS long term.
In the present study, we examined the relation between CGS and eye movement behavior while looking at food cues, extending Potthoff and Schienle's (2020) research. Gaze behavior was recorded with an EyeLink1000 during the presentation of stimulus pairs consisting of high-caloric (HC), low-caloric (LC), and non-food (NF) items in three combinations (HC-LC; HC-NF; LC-NF). CGS was assessed with a novel questionnaire.
Results indicate that a strong reliance of cognitive functioning on glucose sources (high CGS) manifests in gaze behavior, e.g., distinct processing of certain high-caloric foods. This supports the idea of visual attention guidance as a possible underlying mechanism in a short-term compensatory strategy to avoid imminent performance decline. These results support the notion that CGS plays a central role in the emergence and maintenance of maladaptive eating behaviors.

Dynamical Accommodation of Overt Attention, Accuracy and Time in Modern Pentathlon Athletes during Laser Run Shooting

Dalila Sciarra[1], Tommaso Palombi[1], Federica Galli[2], Alessandra De Maria[2], Dario Fegatelli[1], Andrea Chirico[1], Moreno Coco[1], Fabio Lucidi[1]
[1]Sapienza University, Italy; [2]University of Rome "Foro Italico", Italy
dalila.sciarra@uniroma1.it

Many sports require athletes to dynamically control their visual attention to achieve optimal performance, especially when under time-pressure. In this real-world eye-tracking study, we uncover the interplay between attention and performance in 11 Modern Pentathlon elite athletes of the Italian National Team during "Laser Run", which requires athletes to achieve five valid shots in four series. In real competitions, following each series, athletes complete a run, which forces them to trade-off accuracy (how well they hit the target) and time (how fast they shoot). In this study, athletes completed five blocks of four shootings series. Fixations acquired with a Tobii Pro Glasses 2 were time-locked to each critical shoot (when the trigger was pulled) and considered only if occurring within a single or spanning across multiple shoots. While we found that higher accuracy and longer fixations increase the time to complete a shoot, elite athletes learn to dynamically adjust this trade-off between attention and accuracy across the shooting blocks, so significantly reducing their overall timings too. Our results show a direct relationship between eye movement and performance in terms of timing and accuracy, emphasizing the necessity for attention to dynamically accommodate motor-actions in time-limited sport activities.

Saccade latency and metrics in the interleaved pro- and anti-saccade task in athletes

Chin-An Wang[1,2], Cesar Barquero[3], Wei-Kuang Liang[3]
[1]Taipei Medical University, Taiwan; [2]Shuang Ho Hospital, Taipei Medical University, Taiwan; [3]National Central University, Taiwan
josh.wang@tmu.edu.tw

Evidence has demonstrated that athletes exhibit superior cognitive performance associated with executive control. In the oculomotor system, this function has been examined using the interleaved pro-saccade and anti-saccade task (IPAST), wherein subjects are instructed to either automatically look at the peripheral target (pro-saccade) or suppress the automatic response and voluntarily look in the opposite direction (anti-saccade). We examined saccade latency and metrics in the IPAST among athletes (N=40) and non-athletes (N=40), hypothesizing enhanced performance among athletes. Higher direction error rates were obtained in the anti-saccade compared to the pro-saccade condition, with no differences between athletes and non-athletes noted. Faster saccade latencies were observed in athletes compared to non-athletes in both conditions. Furthermore, athletes demonstrated a higher frequency of anticipatory saccades compared to non-athletes, and a significant main sequence relationship between saccade peak velocity and amplitude in anticipatory saccades was observed in athletes but not in non-athletes. Additionally, athletes showed significantly higher frequencies and faster latencies of express saccades compared to non-athletes. Overall, these findings demonstrate that athletes displayed superior saccade performance associated with sensorimotor and preparatory processing, highlighting the potential of using saccade latency and metrics to objectively investigate sensory, motor, and cognitive functions in athletes.

iSCAN project: Examination of gaze behavior in social anxiety disorder using a virtual reality eye-tracking paradigm: a case–control study

Fatime Zeka[1,2], Lars Clemmensen[1], Benjamin Arnfred[1], Mette Øllgaard Pedersen[3], Merete Nordentoft[4], Teresa Hirzle[5], Louise Birkedal Glenthøj[1,2]
[1]VIRTU Research Group, Copenhagen Research Center for Mental Health CORE, Mental Health Center Copenhagen, Denmark; [2]University of Copenhagen, Department of Psychology, Denmark; [3]Psykoterapeutisk Center Stolpegård, Region Hovedstadens Psykiatri, Denmark; [4]Copenhagen Research Centre for Mental Health CORE, Mental Health Center Copenhagen, University of Copenhagen, Denmark; [5]University of Copenhagen, Department of Computer Science, Denmark;
fatime.zeka@regionh.dk

Objective: Social Anxiety Disorder (SAD) is a prevalent and debilitating disorder. It is considered a risk factor for developing other mental disorders, stressing the importance of early detection specifically by examining potential behavioral markers of the disorder. The advances within Virtual Reality (VR), involving the integration of eye-tracking technology in head mounted VR display is allowing research to be conducted with high ecological validity since VR enables simulation of real-life environments. The objective of the iSCAN project is to examine gaze behavior as a behavioral marker in SAD utilizing a VR-based eye-tracking paradigm.
Methodology: Participants were presented to stimuli in VR consisting of high-resolution 360° 3D stereoscopic videos of three social-evaluative tasks designed to elicit social anxiety. Outcome measures are fixation-based parameters and raw data parameters.
Results: Thirty individuals with SAD and a matched healthy control group of thirty individuals were recruited between January 2023 and February 2024. The study investigated between-group gaze behavior differences during stimuli presentation. The analysis of the data has begun, and results will be presented at the conference.
Conclusion: The findings may advance the current understanding of gaze behavior in SAD and contribute to the scarce research using VR eye-tracking for assessment of mental disorders.

Wednesday - afternoon - Individual Differences and Special Populations in Reading - Venue C

A corpus study of length, frequency, and surprisal effects in deaf and hearing readers

Frances Grosvenor Cooley[1], Karen Emmorey[2], Emily Saunders[2], Grace Sinclair[3], Casey Stringer[3], Elizabeth Schotter[3]
[1]Rochester Institute of Technology, United States of America; [2]San Diego State University, United States of America; [3]University of South Florida, United States of America
fgcdls@rit.edu

Deaf readers are a unique population of efficient readers, demonstrating increased skipping and decreased fixation durations without a negative impact on comprehension. While skilled deaf readers reportedly have a wider perceptual span to the left and right of fixation, the information to which they attend in their wider span to support their efficient reading remains unclear. We report results from a corpus analysis comparing the effects of the "Big Three", word length, frequency, and context predictability/surprisal, on skilled deaf and matched hearing readers' word skipping and gaze durations while reading 200 sentences. Deaf readers demonstrated increased skipping and shorter gaze durations compared to hearing readers and exhibited stronger effects of all three word characteristics. Deaf readers skipped the longest, lowest frequency, and least predictable words at similar rates to hearing readers, but showed increased skipping for all other words. Deaf readers spent less time reading short, high frequency, and predictable words compared to hearing readers, but did not differ on the most difficult words. Thus, deaf readers exhibit reading efficiency most prominently for easy to process words, perhaps because their wider perceptual span allows such words to be quickly processed at multiple time points throughout the timecourse of reading.

Semantic and attentional processing during reading in adults with dyslexia

Ciara Egan[1], Joshua S. Payne[2,3], Manon W. Jones[2]
[1]School of Psychology, University of Galway, Ireland; [2]School of Human and Behavioural Sciences, Bangor University, Wales, LL57 2AS; [3]School of Psychology, Glyndŵr University, Wales, LL11 2AW
ciara.egan@universityofgalway.ie

Readers with developmental dyslexia are thought to be impaired in representing and accessing phonology from text, but their ability to process meaning is generally considered intact. Here, we simultaneously recorded event-related potentials (ERPs) and pupil dilation from adults with and without dyslexia while they completed semantic congruency judgements. Dyslexic readers showed an overall reduction in the ability to accurately judge semantic congruency, suggestive of a subtle semantic impairment. Whilst no group differences emerged in the electrophysiological measures, our pupil dilation measurements revealed a reduced attentional response to these word stimuli compared with typical readers. This study represents the first investigation into pupillary responses to lexical input in readers with dyslexia. Our results show a broad manifestation of neurocognitive differences in processing of print between adults with and without dyslexia, at the level of autonomic arousal as well as in higher level semantic judgements.

The unique eye movement profile of deaf readers reveals the plasticity of the reading system

Karen Emmorey[1], Casey Stringer[2], Emily Saunders[1], Grace Sinclair[2], Frances Cooley[3], Elizabeth Schotter[1]
[1]San Diego State University, United States of America; [2]Univesity of South Florida, United States of America; [3]Rochester Institute for Technology; kemmorey@sdsu.edu

A unique reading profile is emerging for deaf individuals who are early signers that differs from reading-matched hearing individuals. Despite weaker phonological skills, deaf readers with robust early language exposure exhibit very efficient eye movement behaviors while reading. Evidence from moving-window studies reveals a unique profile for deaf signers. Compared to their hearing peers, deaf people read faster, skip more words, and generally have shorter fixation durations, without loss of comprehension. In addition, deaf readers have a larger rightward reading span, which provides a wider parafoveal preview, and a larger leftward span, which enhances their ability to confirm previously read text. Like hearing readers, reading skill is positively correlated with the size of the rightward span; however, unlike hearing readers, it is also correlated with the size of the leftward span. Thus, reading skill for deaf people is supported by a wider, bilateral distribution of visual attention. The mechanism that gives rise to this distinct reading profile is unclear, but one likely possibility is the redistribution of attentional resources to the visual periphery, which is associated with early deafness and/or extensive experience with sign language processing. This deaf-specific reading profile highlights the experience-dependent plasticity of the reading system.

Exploring the impact of individual differences and reading goals on eye movements and comprehension of academic texts

Fernando Moncada
Pontificia Universidad Catòlica de Valparaìso, Chile
fernando.moncada@pucv.cl

This ongoing study explores how individual differences and reading goals influence eye movement patterns and comprehension during the reading of academic texts. In a repeated measures design, forty Chilean undergraduates completed three questionnaires to assess various factors associated with individual differences (Need for Cognition, Reading Experience, and Reader's Approach to Text), in addition to a standardized reading comprehension test (Deep Cloze Test). In the eye-tracking task, participants read 12 specialized texts (150-170 words) under two conditions: reading to answer True/False items or multiple-choice questions, simulating common study tasks. Texts were counterbalanced across conditions and participants. After reading each text, participants performed the task on which they were instructed according to their reading goal. Eye movement measures, such as regressions, fixation times, and transitions between regions of interest, as well as comprehension performance, were analyzed. Preliminary results suggest that readers with higher scores in Need for Cognition and deep approaches to reading exhibit more fixations, longer fixations, and more regressions, especially in key regions, which is associated with better comprehension in both conditions. In addition, preliminary analyses suggest potential differences in eye movement patterns between the True/False and multiple-choice conditions, which will be explored further in subsequent analyses.

"老马识途" ("An Old Horse Knows the Direction"): Older Adults have Greater Difficulty Reading Transposed Chinese Idioms

Jingxin Wang[1], Xuran Cao[1], Fengjun Zhang[1], Kuo Zhang[2], Kevin B. Paterson[3]
[1]Tianjin Normal University, People's Republic of China; [2]Nankai University, People's Republic of China;
[3]University of Leicester, UK; wjxpsy@126.com

Written Chinese contains a large number of idiomatic four-character words that often are used figuratively. Many of these idioms are created by the concatenation of two parallel word-pairs. For instance, the idiom "春暖花开" ("spring warm, flowers bloom", meaning the scenery in spring is beautiful) is highly familiar to readers. With the present experiment, we investigated the potential for older readers to more strongly expect highly-familiar idioms to conform to their normal word order, and therefore to have greater difficulty compared to young adults when processing sentences containing its reversed forms. Young (18-30 years) and old (65+ years) adults read sentences containing normal idioms (e.g., 春暖花开) and its reversed forms (e.g., 花开春暖). Both age groups had longer reading times for reversed forms compared to normal forms, although this reading time cost was larger for older adults. We discuss these findings in relation to theories proposing reduced cognitive flexibility in old compared to young adults, possibly due to old readers having greater knowledge and experience of written language.

The role of text- and reader-related factors in narrative engagement when reading translated fiction: An eye-tracking study

Tim J. Smith, Jonathan P. Batten, Boguslawa Whyatt, Olga Witczak, Ewa Tomczak-Łukaszewska, Olha Lehka Paul, Maria Kuczek, Agata Kucharska
Faculty of English, Adam Mickiewicz University, Poznan, Poland
bwhyatt@amu.edu.pl

The research into whole text reading has shown that there are many text- and reader-related variables which contribute to the overall reading experience. In this presentation we report on an experimental eye-tracking study in which three groups of readers (3x n20) with different levels of L2 proficiency read three excerpts from popular fiction translated from English (the readers' L2) into Polish (the readers' L1) while their eye movements were recorded by EyeLink 1000 Plus. After reading an excerpt, the participants rated their narrative engagement on a Transportation Scale (Appel et al. 2015). The three excerpts (~460 words) came from published official translations and differed in text complexity and translation quality. The results show that for all readers, reading a low-quality translation and a more complex text required more cognitive effort indexed by fixation duration (per word) and resulted in lower scores on the transportation scale than reading a high-quality and less complex text. Although, the effect of *L2 proficiency* on the eye movement measures and narrative engagement was not as prominent as expected, we found significant *group x text* interaction effects. The findings contribute to the under-researched area of translation reception and a better understanding of the effects of bilingualism.

Thursday - morning - Image and Scene Perception - Venue A

Evaluating Emotional Perception in Qajar and Neoclassical Portraits: An Eye-tracking Approach

Yasaman Farhangpour
University of Milan, Italy; yasaman.farhangpour@unimi.it

To delve into the intricate nuances of emotional perception within visual art, understanding how individuals receive and interpret emotional cues is imperative. Leveraging eye-tracking technology offers a pivotal starting point for investigating these dynamics. This study undertakes a comparative exploration of emotional perception within portraits from the Qajar and Neoclassical eras. It scrutinizes the impact of these emotions on eye movements and scene perception, particularly in contrast to traditional face-to-face viewing experiences. Selected portraits are treated as contemporary styles with visual patterns analyzed among 20 subjects exposed to 40 stimuli categorized by conveyed emotions. Participants also completed questionnaires, including the BIG Five personality traits test, the AJSS questionnaire for aesthetic judgment styles, and inquiries regarding general interest in art, aiming to discern correlations with emotional perception. The findings are assessed through correlation and regression analyses. Employing a comprehensive methodology, this study endeavors to contribute to the ongoing discourse on art perception, while shedding light on how technological advancements enhance our comprehension of the intricate interplay between human cognition and visual art.

Does context matter? (Minimal) effects of minimal context on eye movements in scene viewing

Tom Foulsham
University of Essex, United Kingdom
foulsham@essex.ac.uk

There are many descriptions of where people look in scenes, and models for predicting this. Almost all of this work is based on experiments with randomly ordered scenes. However, in the real world, as well as in media such as film and comics, the sequence of images that we see is predictable. I will describe a scene encoding procedure where a minimal amount of sequential context is introduced. Participants are shown a series of scenes blocked according to category (gist), such that several images from the same category are displayed one after the other. If the context matters, then high context images appearing after several similar scenes will be inspected differently due to recent category experience. However, the results of two experiments show only minor effects of sequential context. Changes in fixations and saccades over the course of the trial were consistent with a move from global to local processing, but this did not vary with context. This indicates that, at least in these experiments, participants start with a blank slate and are not influenced by the preceding images. I will discuss these results with reference to models of scene viewing and other influences of context on where people look.

Reading scenes: Evidence for narrative guidance of visual attention in scene perception

Jochen Laubrock, Pelin Çelikkol, Eslem Alemdar, David Schlangen
University of Potsdam, Germany
jochen.laubrock@uni-potsdam.de

What guides our attention when viewing visual scenes? Early models of scene perception emphasized saliency computed from low-level visual feature. In recent years evidence for semantic and syntactic guidance of attention in scene perception has accumulated. Here we go a step further and show that the narrative structure of scenes influences how and when we attend to objects in a scene. We compare verbal scene descriptions generated by human participants or a multimodal large language model with eyetracking data generated by a different set of human participants. Both fixation durations and fixation probabilities were associated with when objects were mentioned in the verbal descriptions. Furthermore, we adopted the Term Frequency - Inverse Document Frequency (TF-IDF) metric to object-scene category relations. TF-IDF computed on the verbal descriptions as a measure of object relevance for the scene predicted fixation selection: more relevant objects were fixated earlier and for longer. Our work shows that linguistic analyses can be used to predict where and when people will attend in visual scenes, suggesting that a higher level cognitive narrative structure guides attention allocation. Thus it appears as if a common underlying cognitive process is responsible for sequencing in language production and eye movement control.

Unraveling Gaze Dynamics Towards Familiar Items: Implications and Insights

Yoni Pertzov, Oryah Lancry Dayan, Tal Nahari, Gershon Ben-Shakhar
The Hebrew University of Jerusalem, Israel
pertzov@gmail.com

Understanding human gaze dynamics towards familiar items holds significant implications across various research domains, spanning psychology, neuroscience, and applied fields such as concealed information tests and marketing. My talk will include a synthesis of studies that explored the intricate patterns of gaze dynamics towards familiar faces and objects across diverse populations and task instructions. Our findings consistently demonstrate a default tendency to prioritize familiar items within the initial seconds of visual exploration. Interestingly, this bias undergoes a distinct reversal when task demands prioritize unfamiliar stimuli, persisting despite voluntary attempts to control gaze. Notably, this pattern persists even in individuals with prosopagnosia when they do not explicitly recognize the presented face, underscoring the robustness of the underlying cognitive processes. The divergent gaze patterns towards familiar and unfamiliar items carry significant implications for detecting concealed knowledge, particularly in forensic settings where accurate identification of relevant information is crucial. By elucidating the underlying cognitive mechanisms driving gaze dynamics, this research not only enhances theoretical understanding but also offers practical applications in various domains.

Exploring visitor engagement through eye-tracking analysis at a science exhibition

Stanislav Popelka, Jiří Vysloužil
Palacký University Olomouc, Czech Republic
stanislav.popelka@upol.cz

The aim of the submission is to introduce a study aiming to evaluate the attractiveness of a geographic exhibition. A total of 36 participants attended the study. They were recruited from real visitors and were divided into three groups – children, teenagers, and adults. Data collection took place in the "Living Water" exhibition at Fort Science – an interactive science centre in Olomouc, Czech Republic.
Tobii Pro Glasses 3 were used for data collection. The quality of recorded data was assessed using GlassesValidator. For data analysis, Tobii Pro Lab software was used. Using the snapshot function, the fixations were projected onto photographs of the exhibits. Distribution of the attention among marked AOIs was visualized using sequence charts displayed using the open-source application GazePlotter.
The exhibition's appeal was evaluated through an analysis of the sequence in which exhibits were visited, the duration of interaction, and a thorough examination of how participants focused on AOIs. The outcomes facilitated the categorization of exhibits based on their attractiveness and led to recommendations for rearranging the exhibits to enhance the overall visitor experience. Eye-tracking showed that textual descriptions were frequently overlooked. These insights have been shared with the Fort Science staff to refine the centre.

All Eyes on The Cyber Canvas: Expert and Non-expert Online Viewing Patterns, Preferences and Memory of AI and Human Paintings

Bernard Matthew Værnes, Thomas Espeseth
University of Oslo, Norway; bernard.vaernes@gmail.com

Digitization has given artists and non-artists easy access to a variety of art forms, from abstract to figurative, and recently works created by artificial intelligence. This study aims to assess how visual art expertise impacts viewing patterns, decision making and memory retention of different human and AI works when viewed in an online (real life) setting. To achieve this, 120 images of different works of art from the collection of the Norwegian National Gallery, and 120 equivalent AI generated works are presented to visual art students from Fine Art Academies and University Art programs, and non-art University students from Europe on their own iPhone screens, while their selfie camera records their eye-movements. Participants rate the aesthetic appeal of each painting, and then complete a memory test of the previously viewed and new human and AI works. Eye tracking data is analyzed for the different groups and stimuli, together with with memory scores and aesthetic evaluations in order to objectively compare expert's and non-expert's visual processing differences, aesthetic preferences and memory of images of different human and AI works in a real life setting. The findings could be relevant for galleries, artists, graphic and UX designers, and educators.

Thursday - morning - Reading in Different Languages - Venue B

The Role of Ligatures in Arabic Reading: Evidence from Eye Movements

Maryam A. AlJassmi[1,2,3], Aysha Alsharif[3], Manuel Perea[4], Kevin B. Paterson[2]
[1]UAE University, United Arab Emirates; [2]University of Leicester, UK; [3]Zayed University, UAE;
[4]Universitat de València, Valencia, Spain
maryam.aljassmi@uaeu.ac.ae

Previous studies have shown that increasing the length of Arabic words by adding letters increases fixation times and decreases word-skipping. We examined the effects of ligatures (connecting lines between letters), which widen words for aesthetic reasons without adding letters, in two experiments using the same stimuli. Experiment 1 adjusted the width of all words in sentences, while Experiment 2 focused on a specific target word, assessing the effects of no ligatures (محيط) versus adding one (محيط) or two (محيط) ligatures. In both experiments, we manipulated target word frequency to test whether the effect of the ligatures modulates lexical access beyond letter-encoding. Experiment 1 showed that adding ligatures increased reading times and reduced word-skipping, including for the target words. Experiment 2 replicated this effect for the target words. Effects of word frequency were obtained regardless of the number of added ligatures. Thus, increasing a word's length by adding ligatures can influence both the probability of the reader fixating a word and the time spent reading it without affecting the recognition of the word. We discuss these findings in relation to the use of ligatures for both aesthetic reasons and to emphasize words in text, and the **theoretical implications for Arabic reading.**

Lexical Processing in Urdu: An eye tracking study

Rizwana Azeez[1], Padakannaya Prakash[2]
[1]JSS Academy of Higher Education and Research, India; [2]Christ University, Bengaluru, India
azeez@jssuni.edu.in

Urdu, an Indo-Aryan language spoken in many countries worldwide, particularly South Asia, is similar to Hindi in syntax, grammar and stem-based morphology with Nastaliq script and words extensively borrowed from Arabic. This linguistic/orthographic amalgamation makes Urdu a unique case for studying lexical access. We examined lexical access of four categories of Urdu words – a) Persian words درد, (dard) b) words with Persian prefix باادب, (ba adab) c) Arabic tri-consonantal root words اخبار, (akhbar) and d) compound words خبرنامه (khabarnama) using a sentence reading paradigm. The target words appeared in the third position in sentences. First fixation duration and dwell time were recorded as early and late processing measures. We were particularly interested in testing whether the tri-consonantal root advantage reported in Arabic lexical access is carried over to Urdu. The results showed that no difference between the conditions on first fixation duration, suggesting no early processing advantage for tri-consonant root words of Arabic origin in Urdu. However, Dwell time was significantly longer for Persian and Arabic root word conditions, suggesting lexical access to borrowed root words demanded more cognitive resources than other common words. This paradox highlights the language/orthographic specificity hypothesis as the tri-consonantal advantage observed in Arabic is absent in Urdu.

Eye-Tracking Measures of Bilingual Irony Processing: A Within-Participant Look at L1 vs. L2 Reading Effects

Vegas Anton Hodgins[1], Mehrgol Tiv[2], Chaimaa El Mouslih[1], Karla Tarin Murillo[1], Antonio Iniesta[1], Debra Titone[1]
[1]McGill University, Canada; [2]US Census Bureau, United States of America
vegas.hodgins@mail.mcgill.ca

Understanding irony is often more difficult than understanding literal utterances, reflected by both early and late measures of eye-tracking during natural reading (Olkoniemi & Kaakinen, 2021). In addition, ironic compliments are known to be marked compared to ironic criticisms (Pexman & Olineck, 2002). Processing ironic utterances such as these requires mentalizing capacity (Tiv et al., 2023). Bilingualism is known to impact social cognition, however, its influence on mentalizing (including irony processing) still requires investigation. In this study, we investigated how bilingual adult readers processed irony in written sentences on first pass reading time, total gaze duration, and go-past time. Contrasting past studies that examined L1 and L2 irony processing between-participants, participants read ironic or literal phrases (compliments and criticisms) in BOTH their L1 and L2, enabling us to examine L1 and L2 irony processing within-participants. Linear mixed effects modelling of data was conducted following a maximal approach. Results indicated no significant effect of L1 or L2 on irony processing and replicated the markedness of ironic complements in L2 processing. However, the extra processing involved in resolving ironic vs. literal phrases dampened the general facilitative impact of increased general language entropy (an individual difference measure) on L2 go-past times.

Task Effect on Multiple-text Reading and Writing: Insights from Eye-tracking Research

Zheng-Hong Guan, Sunny S. J. Lin
National Yang Ming Chiao Tung University, Taiwan
a0935220867@gmail.com

Multiple-text reading is a goal-oriented work where readers integrate texts to construct the document model to address issues. In this study, we employed eye-tracking technology to investigate how different task requirements influence readers' processing of multiple texts with high and low trustworthiness. Sixty-one students were divided into two groups, asking for different tasks: summaries and argumentative essays. Both groups read four texts with conflicting issues addressing genetically modified technology. Each text featured high or low trustworthiness and comprised three paragraphs: source, evidence, and conclusion. The result showed that for high-trustworthiness texts, the argument group spent more first-pass reading time constructing the meaning of the evidence than other paragraphs, whereas the summary group allocated more time to the source and evidence than the conclusion. As for rereading time, regardless of the trustworthiness, both groups reread more of the evidence than other paragraphs. Besides, the summary group spent more time integrating the meaning of source information than the argument group. Regarding writing, the summary group produced more paraphrased responses and utilized more citations, whereas the argument group generated more new information and evaluated the information in the texts. Overall, this study provides robust evidence of a task effect on the document model.

How do bilingual adults draw mentalizing inferences from text? An eye-tracking study of natural reading

Karla Tarin[1], Mehrgol Tiv[2], Antonio Iniesta[1], vegas Hodgins[1], Chaimaa El Mouslih[1], Debra Titone[1]
[1]McGill University, Canada; [2]U.S Census Bureau, United States
karla.tarin@mail.mcgill.ca

Mentalizing skills (i.e., the capacity to attribute mental states to oneself and others) are crucial for social cognition and reading comprehension. However, little is known about mentalizing during natural reading, particularly when people read in a first vs. second language (L1, L2; but see Tiv, O'Regan & Titone, 2023; Navarro, DeLuca & Rossi, 2022). Here, 28 English-dominant and 29 French-dominant bilingual adults read 120 English texts requiring either logical or mentalizing inferences for comprehension (e.g. *"Jane took out the house keys, she locked the front door that day" vs "Jane read about the increase in crime, she locked the front door that day"*). Eye-tracking data revealed no early-stage differences between inference types, however, later-stage measures showed significantly longer reading times and more regressions for mentalizing inferences, particularly among L2 readers. Interestingly, individual variations in language entropy (i.e., how bilinguals compartmentalize language use) moderated reading times and regression counts for L1 readers exclusively. Higher entropy led to increased early and late-stage L1 reading times for mentalizing as well as reduced L1 regression counts for logical inferences, suggesting a heightened attention to mentalizing. Taken together, mentalizing and logical inferences are jointly influenced by text language and readers' language usage patterns.

A Comparison of Monospaced & Proportional Fonts in Arabic Reading: Effects on Foveal and Parafoveal Processing

Kayleigh L Warrington[1], Maryam A AlJassmi[1,2], Sami Boudelaa[2], Kevin B Paterson[1], Manuel Perea[3]
[1]School of Psychology and Vision Sciences, University of Leicester, United Kingdom; [2]Department of Cognitive Science, United Arab Emirates University, UAE; [3]Department of Methodology and ERI-Lectura, Universitat de València, Valencia, Spain
klw53@le.ac.uk

Monospaced, fixed-width fonts, such as Courier, where every character occupies identical horizontal space, are extremely common in eye-movement research in Latinate languages (Slattery, 2016). However, their use has been avoided in Arabic research due to their rarity and perceived unnatural appearance (Hermena et al., 2017), which changes the natural semi-cursive interconnection between letters, and may interfere with processing. The spatial uniformity of monospaced fonts is methodologically advantageous, allowing researchers to match the spatial-width of words containing equal numbers of letters. This imposes difficulties for researchers employing boundary-change techniques in Arabic reading, as the spatial-width of the target and the preview mask cannot readily be matched using a proportional font. The purpose of this study was therefore to investigate the effect of monospaced vs. proportional fonts on linguistic processing and parafoveal preview in Arabic reading. Participants read sentences in a monospaced (Kawkab Mono) or proportional font (Arial). Using the boundary-change paradigm, letters of the parafoveal word were transposed (invalid preview) or written correctly (valid preview) prior to fixation. The results revealed comparable reading times and parafoveal preview benefit, with robust effects of preview validity on fixation durations for both font types. We discuss the methodological implications for research on Arabic reading.

Thursday - morning - Problem-Solving and Expertise - Venue C

Joint visual attention during computer-assisted collaborative problem-solving task: Case study of two dyads with high and low similarities in visual scanpath

Saswati Chaudhuri[1], Ville Heilala[1], Johanna Pöysä-Tarhonen[2], Päivi Häkkinen[2]
[1]Faculty of Education and Psychology, University of Jyväskylä, Finland; [2]Finnish Institute for Educational Research, University of Jyväskylä, Finland
saswati.s.chaudhuri@jyu.fi

Collaborative problem-solving (CPS) is an activity that is performed in pairs/ small groups to achieve a joint goal through problem-solving, communication, and collaboration. During a CPS activity, joint visual attention (JVA) on relevant targets shows the way shared attention is established between individuals. The aim of this study was, first, to investigate JVA through similarities in the visual scanpaths of the two individuals in the dyad(s), and second, to examine in-depth two dyads showing high and low similarity in visual scanpaths. In the present study, 24 sixth grade students' visual attention in 12 dyads were measured using SMI screen-based eye trackers during a computer-assisted collaborative problem-solving task in an online environment. In addition, the data included eye-tracking video recordings and log data, which comprised of their individual interaction with resources in the CPS task and their communication through chat. The ScanMatch Matlab toolbox was used to analyze the similarities in visual scanpath within each dyad, then dyads with highest and lowest similarity indices were chosen for further investigation using a case study approach. Results from case study analyses showed that there were visible differences in CPS process and amount of communication through chat between the two selected cases of dyads.

Eye movements during the verification of arithmetic calculations

Christine Green, Simon Paul Liversedge, Reyhan Furman
University of Central Lancashire, United Kingdom
CGreen11@uclan.ac.uk

Little is known about the cognitive processes involved in addition and subtraction calculations. In three experiments we recorded eye movements during the processing of correct and incorrect arithmetic stimuli, consisting of three-digit sums (hundreds, tens, and units in formal vertical format). Experiment 1 investigated processing of addition and subtraction sums without "carries" or "borrows". Experiment 2 examined operand order effects (e.g. "6+2" vs "2+6") and Experiment 3 investigated "carry procedures" in addition sums. In all experiments the effect of the position of the error was manipulated and analysed.
Results show fixations were positioned most frequently on the digits forming the middle row of sums, showing parafoveal processing of numerical information. Patterns of fixation differentially reflected error detection at different positions within sums (earlier discontinuation of processing for errors in units vs. tens vs. hundred columns respectively), suggesting incremental processing. Preliminary analyses suggest effects associated with operand order and carry/borrow processes. Overall, the eye movement data indicate that mathematical computations follow distinct, step-by-step, computations. The ease with which a sum can be computed affects fixations (duration and number). Foveal and parafoveal processing of visual information is necessary for effective computations.

Enhancing Debugging Performance with Eye Movement Modeling Examples (EMME): An Investigation into Type, Expertise, and Engagement

Kshitij Sharma[1], Halszka Jarodzka[2]
[1]Norwegian University of Science and Technology, Norway; [2]Open Universiteit, the Netherlands
hjr@ou.nl

This study investigates the impact of different types of Eye Movement Modeling Examples (EMME) on debugging performance in novice programmers. We explore three EMME conditions—Teacher-Initiated (T-EMME), Learner-Initiated (L-EMME), and Automated (Auto-EMME)—against a control group, to determine which EMME type is most effective and how programming expertise influences debugging success. Our methodology included designing a system to display EMME alongside programming tasks, with eye-tracking data collected from programming experts. We conducted a between-subject experiment with 120 computer science students, using the Index of Pupillary Activity to measure mental effort and control EMME display in the Auto-EMME condition. Results from an ANOVA indicate significant differences in debugging performance across conditions, with both T-EMME and L-EMME showing superior performance compared to the control and Auto-EMME conditions. No significant difference was found between T-EMME and L-EMME, suggesting that the presence of EMME, rather than its initiation method, is crucial for enhancing debugging performance. Additionally, a positive correlation between programming expertise and debugging success was observed, but only in the control group, implying that EMME may reduce the reliance on prior programming knowledge. Our findings underscore the potential of EMME in programming education and highlight the importance of incorporating eye movement data to support learning.

Exploring Cognitive Load During Geometry Problem Solving Processes by Integrating Eye Movement and Handwriting

Alice R. P. Li[1], Jui-Jen Chou[2], John J. H. Lin[1]
[1]Graduate Institute of Science Education, National Taiwan Normal University, Taiwan; [2]Taipei Fuhsing Private School, Taiwan
alice.renpingli@gmail.com

Eye movement is a sensitive indicator linked to attention and cognitive load. Integrating eye movement with individuals' handwriting traces in a digital environment helps to observe in-depth cognitive processes. Given that both eye movement and handwriting have the potential to identify individuals' difficulties in problem-solving, this study aims to explore the relations between teenage students' cognitive load and their cognitive processes in terms of eye movement and handwriting when they solve geometry problems. We integrated an eye tracker and a tablet with handwriting functions. The digital platform, named Evaluation of Digital Problem-Solving in Geometry (EDPS-G), was developed to record students' eye movement and handwriting data simultaneously during their problem-solving processes. The results indicated that three eye movement indicators: dwell time, fixation count, and saccade length were positively related to an individual's cognitive load in polygon rotation problems, respectively. However, the seven handwriting indicators, including maximum handwriting pressure, suspension count, ratio of suspension time, mean writing speed, and mean writing speed in X and Y directions, were not related to an individual's cognitive load in polygon rotation problems, respectively. The EDPS-G could be helpful for developing a digital environment in which auto-detecting students' problem-solving difficulties is possible.

Automated prediction of problem solving performance using eye movements: Can AI help?

John J. H. Lin, Alice R.P. Li
Graduate Institute of Science Education, National Taiwan Normal University, Taiwan
john.jrhunglin@ntnu.edu.tw

With the ability to predict learning behaviors in complex scenarios, artificial intelligence (AI) shows its potential in assessing the problem-solving performance. Given that AI, data-driven algorithms, could be helpful to address random signals (e.g., random fixations), using AI to predict learning performance based on eye movements seems promising. The aims to explorer the potential of an AI, designed based on the long-short-term memory neural structure, to predict whether mathematics problems could be solved in a digital problem-solving scenario using eye movements. Sixty-one students participated in this study. We examined whether types of eye movement features (AOI-based vs. fixation-based features) and information (Separated vs. integrated steps) during solving problems could impact the performance of AI models. In additional, the effects of two hyper-parameters, activations in hidden layers and number of neuros, were examined. The results suggested that fixation-based features outperform AOI-based features. Furthermore, information in separated steps could provide higher accuracy than that in the integrated steps. Regarding the impact of hyper-parameters on AI performance, the activation function 'tanh' and thirty neurons in each layer could be used to train AI models with higher accuracy. The inconsistent between human and AI-based assessment were discussed and visualized.

Face to face with an expert: Exploring joint visual attention during forensic face comparison and feature comparison in three expert-novice dyads

Christine C. A. van Nooijen[1], Bjorn B. de Koning[1], Ellen Kok[2], Anna Isahakyan[3], Maryam Asoodar[3], Fred Paas[1,4]
[1]Erasmus University Rotterdam, the Netherlands; [2]Universiteit Utrecht, the Netherlands; [3]Maastricht University, the Netherlands; [4]University of Wollongong, Keiraville, Australia vannooijen@essb.eur.nl

Visual domains are professions where comprehension of visual material is central to performance e.g. radiology, air traffic control or forensics. Despite the prominence of apprenticeship as an instructional method in visual domains, the didactic strategies employed by visual experts to scaffold novices during one-on-one interactions remain insufficiently explored.
This multiple case study introduces a contrast-based approach to capturing/analysing dyadic interaction based on joint visual attention (JVA) analysis in long-form interaction data. Three experts working for >10 years at the Netherlands Forensic Institute were recruited to teach novices both how to use a structural checklist on facial features to determine whether the subjects of two photographs were the same person, and how to construct a digital version of a traffic scenario in 3ds-Max to assess car speed. Six sessions were captured with dual mobile eye-tracking. Results are currently being processed but initial analysis shows differences in scaffolding and eye-contact at high/low JVA, and aligns with results from previous research both about expert/novice control during learning and about common expert scaffolding actions.
Isolating segments of high and low JVA captured with mobile eye-tracking allows for a portable and naturalistic contrast-based approach to expert-novice interaction that captures nonverbal actions like gesturing in-camera.

Posters

Monday afternoon – Poster Session 1

Is social cognition a mediator on the relationship between literature consumption and empathy-related pupil dilation?

Daniel John Lee, Ciara Egan
University of Galway, Ireland
D.lee15@nuigalway.ie

Affective empathy (feeling for others) and social cognition (knowing what others are feeling) work in tandem to facilitate social interaction. These constructs are intimately related, yet ultimately dissociable. While research suggests affective empathy is a stable trait, social cognition seems to be a skill that can be learned. Reading fiction is suggested to be one such method to enhance social cognition, with increased print exposure correlated with enhanced social cognitive skills. It is possible, then, that literature consumption is a moderating variable that enables trait affective empathy to be "translated" into learned social cognition. Here we examined this relationship by asking participants to read emotional vignettes, and assessing their own emotional reaction via self-report, while pupil dilation was simultaneously recorded. All participants also underwent a measure of print exposure (Author Recognition Test; ART) and of social cognition (Reading the Mind in the Eyes Test; RMET). Data collection is currently underway (with a planned sample of 55 participants), and we predict that participants with higher print exposure will have greater pupillary responses to emotional vignettes, and that this will be mediated by their RMET scores. We will also present results regarding the pupillary response to various elicited emotions.

Eye movement behaviour during reading across older adulthood

Faye O. Balcombe, Victoria A. McGowan, Sarah J. White
University of Leicester, United Kingdom
fob5@leicester.ac.uk

In comparison to young adult readers (18-35 years), older readers (65+ years) make longer saccades, longer and more frequent fixations, more regressions, and more word skips. A key theoretical account attributes this to a "risky" reading strategy, in which older readers attempt to guess the identities of upcoming words. However, it is not yet known whether this pattern holds across older adulthood. Indeed, studies have previously focused on the "younger-old" (65-75 years), and so we have little understanding of reading in the "older-old" (80+ years). Previous research into the component processes underlying reading has indicated that age-related changes do not always follow a linear trajectory. For example, sensory abilities decline increasingly rapidly with advancing age, whereas vocabulary knowledge appears to increase until young-older age, and then decline in older-old age. Thus, the cognitive mechanisms underlying reading may differ markedly between younger-old and older-old adults. Accordingly, the eye movements of young, younger-old, and older-old participants will be monitored as they read sentences. The findings will provide crucial insights into how the processes underlying reading change across older adulthood, as well as highlighting the methodological challenges involved with researching older populations.

.

The Role of Individual Differences on English Incidental Vocabulary Learning: An Eye-Tracking Study

Meng-Huan Li, Yu-Cin Jian
National Taiwan Normal University, Taiwan
limenghuan04@gmail.com

The present study investigates the role of individual differences in working memory, English proficiency, and English self-evaluation on incidental vocabulary learning from reading English articles. Seventy-one college students in Taiwan read two adapted articles (600-750 words) while their eye movements were recorded. Each article contained six target pseudowords, with exposure frequency ranging of six and two. Participants then completed comprehension questions and three different vocabulary tests. The result from multiple regression analyses showed that English self-evaluation and English proficiency significantly predicted vocabulary learning outcomes. In the 2 occurrences condition, the first fixation duration on target words positively correlated with vocabulary test scores but negatively correlated with working memory scores, indicating that individuals with higher working memory spent less time reading target words and had higher vocabulary learning. Furthermore, gaze duration on target words also predicted learning gains. These findings add new perspectives on how individual differences play a role in incidental vocabulary learning. This study presents the predictive roles of individual differences in the results of English incidental vocabulary learning.

Difference of fixation stability for two progressive lens designs during dynamic visual tasks

Clara Benedi-Garcia, Pablo Concepcion-Grande, Marta Alvarez, Carmen Cano, Amelia Gonzalez, Eva Chamorro, Jose Alonso, Jose Miguel Cleva
Indizen Optical Technologies, Spain
cbenedi@iot.es

Progressive power lenses (PPL) provide the presbyope with sharp vision from far to near distances, despite causing astigmatism outside their central region. Different designs of PPL vary in power and aberration distribution, impacting visual performance for a given task in different ways. Eye-Trackers (ET) provide useful information about visual quality by means of ocular movement metrics like fixation stability, which is measured by bivariate contour ellipse area (BCEA). Tobii Pro Glasses 3 were used to record pupil position of 20 subjects wearing two PPL designs, EndlessSteadyNear and Inmotion (IOT), a specific design for dynamic tasks like driving. They performed 4 dynamic tasks: (1) fixation of a letter which appears at a random position out of 9 possibilities, (2) same task than (1) but 2 possible positions, (3) descending and (4) ascending stairs. BCEA was calculated for each subject and condition and differences between designs were analyzed with a t-test. Averaged BCEA for the four tasks were: (1)0.20±0.17deg2 & 0.15±0.16deg2 for EndlessSteadyNear and InMotion respectively, (2)0.34±0.54deg2 & 0.11±0.10deg2, (3)0.26±0.21deg2 & 0.17±0.08deg2, and (4)0.18±0.07deg2 & 0.17±0.12deg2. Statistically significative differences between designs were found (p-value=0.04). In conclusion, both designs present good performance during dynamic tasks, but fixation stability is higher with InMotion lens.

Promise of open-source, low-cost pupillometry – Contribution to the PupilEXT platform

Gábor László Bényei[1,2], Attila Bertalan Boncsér[3], Péter Pajkossy[1,4]
[1]Department of Cognitive Science, Faculty of Natural Sciences, Budapest University of Technology and Economics, Budapest, Hungary; [2]Neurocognitive Research Center, National Institute of Mental Health, Neurology and Neurosurgery, Budapest, Hungary; [3]Department of Automation and Applied Informatics, Faculty of Electrical Engineering and Informatics, Budapest University of Technology and Economics, Budapest, Hungary; [4]Center for Cognitive Medicine, University of Szeged, Szeged, Hungary
benyeig@edu.bme.hu

Affective empathy (feeling for others) and social cognition (knowing what others are feeling) work in tandem to facilitate social interaction. These constructs are intimately related, yet ultimately dissociable. While research suggests affective empathy is a stable trait, social cognition seems to be a skill that can be learned. Reading fiction is suggested to be one such method to enhance social cognition, with increased print exposure correlated with enhanced social cognitive skills. It is possible, then, that literature consumption is a moderating variable that enables trait affective empathy to be "translated" into learned social cognition. Here we examined this relationship by asking participants to read emotional vignettes, and assessing their own emotional reaction via self-report, while pupil dilation was simultaneously recorded. All participants also underwent a measure of print exposure (Author Recognition Test; ART) and of social cognition (Reading the Mind in the Eyes Test; RMET). Data collection is currently underway (with a planned sample of 55 participants), and we predict that participants with higher print exposure will have greater pupillary responses to emotional vignettes, and that this will be mediated by their RMET scores. We will also present results regarding the pupillary response to various elicited emotions.

Eye movements reveal differences in attentional processing of words and faces between dyslexic and typical readers

Nuala Brady[1], Sean Abarico[1], Jordan Crupper[1], Shannon Lanz[1], Áine Hansberry[2], Meredith Gallagher[2], Maeve Mastri[3], Ciara Egan[2]
[1]University College Dublin, Ireland; [2]University of Galway, Ireland; [3]University of Notre Dame, United States of America
nuala.brady@ucd.ie

Several cognitive skills are involved in reading – including visual recognition, phonological discrimination and spatial attention – and modern accounts of dyslexia recognise multiple routes to this specific reading disorder. For example, reports of co-occurring differences in the processing of words and faces between dyslexic and typical readers suggest that dyslexia may be characterised, in part, by differences in high-level vision. Here we explore the additional role of attention by recording eye movements while participants - 58 adult dyslexic and typical readers - completed both a *lexical decision task* and a face *gender discrimination task*. In Exp 1 stimulus position was varied around a maintained central fixation of 500ms duration providing a measure of the optimal viewing position (OVP) for both word and face stimuli. In Exp 2 participants were free to move their eyes during a 2000 msec trial after an initial brief fixation, revealing difference in saccade direction and fixation duration. Preliminary results confirm reports of differences in the OVP for words between children with and without dyslexia (Valdois et al., 2021) while extending this finding to words and faces in an adult sample. Pupillometry data are used to distinguish participants both within and between the two groups.

GUI-free EOG-based control of smart devices

Tracey Camilleri, Nathaniel Barbara, Matthew Mifsud, Salah Ad-Din Ahmed Youbi, Kenneth Camilleri
University of Malta, Malta
tracey.camilleri@um.edu.mt

Typical smart home setups tailored for individuals with limited fine motor skills often require the use of a graphical user interface (GUI) for device control, whereby users select icons through eye gaze to activate desired device functions. This approach, however, may not be very natural as users are bound to continually focus on a computer screen. This study introduces an alternative innovative paradigm wherein users can initiate device functions by directing their gaze towards the device they wish to control and executing specific eye gestures associated with its functions. Operating in real-time, the system uses the user's position and head orientation to identify the intended device, and utilizes the recorded electrooculographic (EOG) signals to interpret performed eye gestures, thus initiating device functions accordingly. Preliminary findings from a study involving five subjects demonstrate a device selection accuracy of 95.71% when selecting one out of seven smart devices. Additionally, the average cross-validated classification accuracy for seven distinct eye gestures, namely blinks, double blinks, eyes-closed, and upward, downward, leftward and rightward saccades, exceeds 96%. This approach presents a user-friendly alternative for individuals with mobility impairments, offering intuitive control over smart devices, thereby enhancing their autonomy and overall quality of life.

The Differences in Cognitive Processes and Comprehension Results of L2 Readers with Different Language Proficiency Levels: An Eye movement Study

Ting-Ai Chen, YU-CIN JIAN
National Taiwan Normal University, Taiwan
czs93123@gmail.com

The cognitive processes of adult L2 readers during reading-while-listening have been underexplored, with its conflicting effects on comprehension outcomes to be resolved. This study aimed at exploring how the additional audio impacts the eye-movement patterns and the comprehension results of L2 readers with different English proficiency levels. The participants were 87 adult English learners, distributed to either the high-proficiency or low-proficiency groups. Three conditions were designed – reading-while-listening, reading-only with and without time limitation. Each participant had to finish a vocabulary levels pre-test, read two English articles with their eyes monitored, and complete comprehension posttests. The findings in the high-proficiency group support the redundancy principle, whereas those in the low-proficiency group support the dual route theory and print-speech integration. Also, the larger word frequency effect found in this study supports the weaker links hypothesis. Firstly, the low-proficiency participants performed better after reading-while-listening, while the high-proficiency counterparts acquired lower scores. Besides, the high-proficiency group had lower mean fixation duration and longer mean saccade length across the three conditions. Moreover, the word frequency effect was larger in L2 reading, with 74 ms found in the high-proficiency group and 110 ms in the low-proficiency group, compared with 30 ms in first language research.

A new algorithm for identifying saccade movements in eye-tracking experiments.

Pablo Concepcion-Grande, Marta Varela, Marta Álvarez, Clara Benedi-Garcia, Carmen Cano, Amelia Gonzalez, Lorena Lopez, Eva Chamorro, José Alonso, José Miguel Cleva
IOT, Spain
pconcepcion@iot.es

Eye-tracking (ET) systems use algorithms for detecting saccades and fixations based on gaze dispersion, velocity, or acceleration (Duchowski et al., 2000). Low sampling rates in velocity-based algorithms may split saccades or classify them as data loss (Leube & Rifai, 2017). While high-sampling static-ET devices are now common, mobile-ET offers natural scenario recordings at the cost of lower-sampling rates being necessary an accurate saccade analysis. In this work a new saccade identification algorithm for mobile-ET datasets is presented. The algorithm proposed is based on reclassify the movements between two fixations as one unique saccade, recalculating its characteristics. The algorithm was evaluated and compared with data provided by Tobii I-VT fixation filter using data obtained from 28 participants in a study where eye position was recorded using Tobii-Pro-Glasses-3 during two reading tasks at 5.25m and 37cm while using 3 different progressive power lenses. On average, the new algorithm got the saccade count reduced by 18.3±14.0% at 5.25m and 29.2±15.6% at 0.37m and it got the saccade duration increased by 17.9±12.3% at 5.25m and 31.3±26.1% at 0.37m. In conclusion, the implementation of the proposed algorithm successfully integrates fragmented movements, being in accordance with fixation count and leading to correct analysis of saccades.

Individual differences in working memory and lexical quality predict eye-movements, but not fixation-related potentials: Evidence from co-registration of EEG and eye-tracking

Allyson L Copeland, Brennan R Payne
University of Utah, United States of America
allyson.copeland@psych.utah.edu

Individual differences in working memory (WM), literacy skill, and spelling ability have been shown to modulate oculomotor control during reading. However, it is less clear how these constructs predict individual differences in electrophysiological responses (EEG) in natural reading. We examined correlations between a selection of cognitive constructs and both eye-tracking (ET) and EEG outcomes from a co-registration study examining the effects of predictability and plausibility violations in natural reading. Participants (*N* = 44) completed tasks measuring verbal working memory (RSPAN, OSPAN), verbal fluency (FAS) and lexical quality (spelling). We found that higher verbal working memory (RSPAN) and better lexical quality (spelling skill) each predicted faster first fixation durations, gaze durations, and reduced re-fixations. In contrast, only lexical quality predicted overall reading speed (words per minute), regression path duration, skipping, and total reading time. Interestingly, there were no significant correlations between any of the individual difference measures and fixation-related brain potentials (e.g., parafoveal N400 and foveal frontal positivity). These findings suggest that readers with higher spelling ability and verbal WM have more efficient word recognition perhaps driven by better lexical representations and that these differences may be better captured by eye movements than electrophysiological responses in natural reading.

Eyeflow Studio: An Extensible GUI-Based Tool for Dynamic Eye-Tracking Data Processing and Analysis

Wolf Culemann, Angela Heine
University of Duisburg-Essen, Germany
wolf.culemann@uni-due.de

Eye-tracking research, a standard approach to investigate visual attention and perception, often faces challenges regarding data processing, including event detection and assigning fixations to areas of interest (AOI). Even though numerous software solutions are available, many are limited by a lack of interactivity, hardware compatibility and adaptability to scientific progress or require advanced programming skills. Recognising these limitations, we present Eyeflow Studio, a Python-based graphical user interface designed to provide a dynamic and adaptable platform for eye tracking data analysis. Unlike existing solutions, Eyeflow Studio is not only adaptable and extensible through plugins, but also supports interactive visualisations, allowing both automated and semi-automated processing with manual oversight capabilities. The current prototype demonstrates essential features for eye tracking data analysis, especially in the context of reading studies, such as hardware compatibility, event detection, AOI detection/creation and drift correction. By facilitating the integration of new algorithms and deep learning approaches, Eyeflow Studio aims to advance eye tracking research by providing a versatile tool for both experienced researchers and those with limited programming experience. We plan to continue developing Eyeflow Studio in collaboration with the eye tracking community, thereby adding significant value to research methodologies in various fields of application.

Monitoring arousal levels among crew members of a Spanish battleship: An eye-tracking based longitudinal observational study

Leandro L. Di Stasi[1,2], Jesús Vera[1], Beatriz Redondo[1], Ignacio Grueiro Méndez[3], Jaime J. Mas Esquerdo[4], Eduardo A. Gómez Quijano[5], Carolina Díaz-Piedra[1]
[1]University of Granada, Spain; [2]Joint Center University of Granada - Spanish Army Training and Doctrine Command; [3]Buque de Aprovisionamiento de Combate (BAC) "Cantabria" (A-15); [4]Departamento de Psicología, Escuela Militar de Sanidad, Academia Central de la Defensa; [5]Sección de Técnicas de Apoyo a la Decisión, Estado Mayor de la Armada
distasi@ugr.es

Working in a rotating shift schedule often results in not optimal arousal levels, potentially affecting operational safety and crew well-being. Here, we studied arousal variations of 26 members of an auxiliary oiler and replenishment ship operated by the Spanish Navy. We assessed them approximately every 4/6 hours during ten days, before/after the shift and throughout the 24-hour cycle (morning, evening, and night shifts). To record oculomotor indices, we used the Fitness Impairment Tester 2000 (750 Hz, PMI Inc., US). Saccadic (peak) velocity and pupil parameters (constriction, amplitude, and latency) were analyzed as main variables. In addition, we assessed, sleep parameters, perceived levels of fatigue, and reaction times/errors with a short psychomotor vigilance test (Miinsys-Optimal Solution S.A., Chile). During the navigation, crew members slept less than six hours. Perceived fatigue was greater and performance worse before/after and throughout night shifts (F-values > 7). Saccadic (peak) velocity, pupil diameter, and latency varied throughout the 24-hour cycle (F-values > 8.5). Only saccadic (peak) velocity and pupil diameter differentiated between before and after shifts (F-values > 9), with both indices decreasing after shifts. Overall, our findings support the viability of an objective, long-term, periodic monitoring of arousal levels in out-of-the-lab settings.

Art Through the Mind's Eye: How Cognitive Styles Shape Visual Attention with Eastern and Western Masterpieces

Daniel
Haibin Han, Danni Fan, Xingshan Li
Hebei Normal University, Shijiazhuang, Hebei, China
haibinhan@outlook.com

This study explores how individual cognitive styles, delineated into field-independent and field-dependent categories using the Embedded Figures Test, impact the process of appreciating paintings. Participants engaged in viewing celebrated paintings from both Western and Eastern artistic traditions, with a focus specifically directed to three regions of interest within each artwork: face, theme, and context. Results revealed that field-dependent individuals showed significantly higher average number of fixations on context, highlighting their pronounced focus on contextual elements of the artwork compared to field-independent participants. Interestingly, field-independent viewers directed significantly more attention towards theme of the paintings, underscoring their propensity to engage more deeply with the central narratives and subjects of the artworks. However, no significant differences were observed in the attention paid to faces across both groups. These findings illuminate the profound impact of cognitive styles on visual attention distribution during art appreciation, suggesting that field-dependent viewers tend to allocate greater attention to peripheral and contextual details, whereas field-independent viewers prioritize central themes and salient features of artworks. This differential focus not only highlights the nuanced ways in which cognitive predispositions can shape the perceptual experience of art but also enhances our understanding of the psychological underpinnings of art interpretation.

Do Chinese readers of English find text in capitals hard to read?

Xi Fan[1], Ronan Reilly[2]
[1]Guangzhou Medical University, China; [2]Maynooth University, Ireland
ronan.reilly@mu.ie

This paper describes a replication of a classic study of case-change preview effects (Rayner, McConkie, & Zola, 1980), using Chinese readers of English as a second language (L2 readers). There is some evidence (Yang & Fu, 2019) that Chinese L2 readers tend to have greater difficulty reading words in uppercase than their L1 counterparts. To explore the locus of this difference, we collected data from 64 participants, controlling for English oral and reading ability. The task involved showing a parafoveal preview, either in upper- or lowercase, of a target stimulus displayed in either upper- or lowercase. Participants were instructed initially to fixate a central dot and to move their eyes to the location of the right parafoveally presented stimulus as rapidly as possible and to indicate by keypress if the stimulus was a word or not. During the saccade, the preview stimulus was replaced by the target. The results showed that L2 readers' responses were significantly less facilitated by an uppercase preview than those of L1 readers. A convolutional neural network (CNN) trained using similar letter statistics as those encountered by Chinese readers, reproduced a similar pattern of results.

Exploring the role of the macaque lateral intraparietal area in voluntary and reflexive saccadic eye movements

Sadra Fathkhani[1,2], Bahareh Taghizadeh-Sarshouri[1,2], Andre Kaminiarz[1,2], Frank Bremmer[1,2]
[1]Applied Physics and Neurophysics, Philipps University Marburg, Germany.; [2]Center for Mind, Brain and Behavior, Universities of Marburg, Giessen, and Darmstadt, Germany;
sadra.fathkhani.de@gmail.com

The macaque lateral intraparietal area (area LIP) is involved in the processing of voluntary saccades. Here, we asked if the same neurons are also involved in the processing of reflexive saccades, in this case the fast phases of an Optokinetic Nystagmus (OKN). We compared spiking neural activity in area LIP of two rhesus macaques across two types of eye movements. Neurons were first tested using a visually guided saccade paradigm. Neurons that had a significant ($p<0.05$) peri-saccadic response were further investigated for their involvement in the processing of reflexive eye movements. OKN, i.e. an alternation of smooth and fast resetting eye movements, was induced by large field frontoparallel motion of a random dot pattern. The direction of dot-motion was chosen in a way that the elicited fast phases were aligned with the neuron's preferred saccade direction and shared kinematic properties with saccades. We found that despite enhanced spiking activity during visually guided saccades, the same neurons exhibited close-to-baseline or even reduced activity during the fast phases of the OKN. We conclude that area LIP is not primarily concerned with the generation of motor commands for fast eye movements but with goal directed interaction and target selection in the visual environment.

Effects of Pupil Swim on Eye-Tracking in Virtual Reality

Hunter Christian Finney, Sarah H. Creem-Regehr, Jeanine K. Stefanucci
University of Utah, United States of America
hunter@hunterfinney.com

Eye-tracking is a relatively recent addition to commercially available head-mounted displays (HMDs). Further, the sampling rate of these eye trackers is generally lower than what has been previously used in carefully controlled research studies. For immersive virtual environments (IVEs), it is important to know if the image rendered is properly projected to the user without distortions in order to accurately perceive scale. One such distortion is global pupil swim, a phenomenon that occurs when the user's gaze moves outside of the lens' exit pupil projecting the image. Convergence is a common action that causes such occurrence. We conducted a study using the Varjo XR-3 and the integrated 200Hz eye-tracker to uncover if a user's gaze was accurately represented. Calibration of eye-tracking and interpupillary distance were done prior to the experiment. The subject then looked at targets monocularly with their dominant eye. The targets appeared centered on their field of vision and progressively came closer to the subject, requiring a steeper convergence angle. We discovered that the eye-tracking reported the user's gaze was shifted in the same direction as the distortion induced by pupil swim. Consequently, eye-tracking reported inaccurate fixations of targets outside the exit pupil.

Parafoveal processing of word length and compound words in English children

Donna Gill, Simon P. Liversedge, Sara V. Milledge
University of Central Lancashire, United Kingdom
degill@uclan.ac.uk

The word length constraint hypothesis (Inhoff et al., 2003) proposes that word length information for word *n*+1 (the upcoming word) constrains lexical candidates. Juhasz et al. (2008) found support for this in adults. A review of Kuperman et al.'s (2012) Age-of-Acquisition database shows that young children have fewer long words in their mental lexicons, and average word length increases with age. Thus, it is conceivable that word length may provide a greater cue as to the identity of word *n*+1 for children, resulting in a stronger word length constraint effect. In the present study, 7–8-year-old children and adults were presented with sentences using the boundary paradigm (Rayner, 1975). Participants were either given a correct length (e.g., *pancake – pancake*) or incorrect length preview by manipulating the first or second lexeme of a compound or a matched monomorphemic word (e.g., *pan ake – pancake*; *bro nie – brownie*). We are currently analysing our results. We anticipate that word length will constrain lexical identification more in children than adults, such that inaccurate length previews will cause greater disruption for children. The results will provide insight into how word length constrains parafoveal processing in children and how children process compound words in English.

Who is addicted to games? Comparison of attentional bias through eye movements among e-athletes, gaming addicted, and normal gamers

Zheng-Hong Guan[1], Shan-Mei Chang[2]
[1]National Yang Ming Chiao Tung University, Taiwan; [2]National Tsing Hua University, Taiwan
a0935220867@gmail.com

Based on Incentive Sensitization Theory, previous research has observed attentional biases in gaming disorders, but there are few studies comparing differences between e-athletes and addicted gamers. In this study, the dot-probe task with eye-tracking technology was used to investigate attentional biases and visual processing. Fifty-two participants were categorized into three groups based on their Internet Gaming Disorder tests: Gaming Disorder (GD), non-GD and e-athletes. Participants underwent stimulus and control trials with two images each. GD showed a longer first fixation duration for all images and also a longer total viewing time on the stimulus images than neutral images; other groups did not show this effect. It shows the attentional bias of GD individuals in that they allocated more total viewing time to the stimulus image compared to the neutral one. GD and e-sports had comparable higher scores for self-report gaming time and depression than non-GD. Only GD demonstrated impulsivity traits. These suggest excessive gaming alone (both e-sports and GD) does not necessarily indicate gaming addiction. The diagnosis of gaming disorder based on gaming time alone should be taken with caution. Future research should further investigate reasons why both GD and e-athletes exhibit higher levels of depression compared to recreational gamers.
.

Saccade Automaticity and Sense of Agency

Julian Gutzeit, Lynn Huestegge
University of Würzburg, Germany
julian.gutzeit@uni-wuerzburg.de

Sense of agency (SoA) refers to the feeling that one is in control of one's actions and their resulting effects. As we usually do not manipulate our environment using our gaze, corresponding research in the oculomotor domain has so far been rather sparse. We examined the impact of different levels of action voluntariness on SoA by manipulating saccade automaticity: Participants performed pro- and anti-saccades in response to peripheral stimuli. By fixating those stimuli, participants changed the stimulus color. We hypothesized that anti-saccades (vs. relatively automatic pro-saccades) would lead to stronger SoA due to a high level of voluntary action control.
We found that temporal action binding, a phenomenon often interpreted as an implicit measure of SoA, was stronger for anti-saccades compared to pro-saccades. However, temporal effect binding was stronger for pro-saccades than for anti-saccades.
These results indicate that increased levels of voluntary action control enhance the implicit SoA for actions, whereas a reduced need for action control increases the implicit SoA for effects, likely due to decreased effort. These results are well in line with recent accounts emphasizing the importance of considering the SoA for actions and effects as distinct theoretical constructs.

Parafoveal Processing of Kanji Characters during Reading Japanese

Miyuki Henning[1,3], Chie Nakamura[2], Laura Schwalm[1], Ralph Radach[1]
[1]University of Wuppertal, Germany; [2]Waseda University, Japan; [3]University of Cologne, Germany;
miyuki.henning@uni-wuppertal.de

Orthographic characteristics of a written language determine what kind of information can be extracted from the parafovea during reading. Considering the complex writing system of Japanese, combining three script types, it is surprising that little research has been conducted with regard to this language. The present work examined parafoveal processing of two-character kanji words using a gaze-contingent boundary paradigm in single-line Japanese sentences. The preview was either identical, or orthographically, phonologically, semantically similar, or unrelated to the target (e.g., 索 (/saku*/; search*) vs. 素 (/su/; *basis*) vs. 削 (/saku/; *reduce*) vs. 探 (/tan/; search) vs. 黄 (/ki/; yellow)). Results indicated a substantial increase of viewing times in all conditions when compared to the viewing times in the identical condition. Compared to the unrelated condition, there was a substantial preview benefit when an orthographically similar preview was presented and a weaker benefit in the semantic condition. Further, there was no preview benefit in the phonologically similar condition. These results indicate that orthographic as well as semantic information can be extracted from the parafovea during reading of kanji characters in Japanese. Theoretical implications and similarities to work on reading in Chinese will be discussed

Stem Alternations in Easy Language and L2 Reading Fluency

Rosa Salmela[1,2], Anniina Laurila[1], Minna Lehtonen[1], Raymond Bertram[1]
[1]University of Turku, Finland; [2]Åbo Akademi University, Finland
rnsalm@utu.fi

Easy language, adapted for improved readability and comprehension, serves as an auxiliary form of communication for individuals with weakened language skills due to injury, illness, or neurological conditions. Additionally, it serves as an aid for second language learners (L2). Despite its significance, easy language has received little attention in experimental research. This study explores how simplifying the complex morphology of Finnish can facilitate reading for L2 learners. Finnish morphology entails inflecting words (auto: auto+ssa; 'car: in a car'); sometimes these inflections involve morphophonological stem alternations (vesi: vede+ssä; 'water: in the water'). Using eye-tracking methodology, we investigated the reading fluency of beginner-level L2 speakers (n = 28) as they read short Finnish stories in easy language, both with and without stem alternations. The results indicate that while stem alternations did not significantly impact reading fluency in on-target fixation durations, they interacted with language skills in late measures. Specifically, a spill-over effect was observed on the target+1 words in the low-proficiency group, affecting both gaze durations and total fixation durations. These findings suggest that reducing morphophonological changes can enhance the readability of easy language in Finnish, particularly for low-proficiency groups, and highlight the importance of addressing morphophonological complexity in language learning contexts.

Individual text corpora for predicting personality and eye movements during reading

Markus J. Hofmann, Markus T. Jansen, Christoph Wigbels, Ralph Radach
University of Wuppertal, Germany
mhofmann@uni-wuppertal.de

While different word corpora have frequently been discussed in psycholinguistics, here we take this discussion down to the individual level by text corpora generated for each participant. We assume that individual text corpora (ICs) reflect a sample of unique human experience and train language models reflecting the semantic memory of each participant. In study 1, we collected ICs from two participants that followed their everyday reading behavior for two months on a tablet (Hofmann et al., 2020). We computed word probabilities from the two ICs and a standard corpus. Then we selected 134 stimulus sentences that provide uncorrelated word probabilities. In the subsequent eye-tracking study, we found that these ICs, but not the standard corpus, can predict first fixation durations, suggesting rapid access to recently acquired individual memories. In study 2, we collected ICs from the internet search history of 214 participants (Hofmann et al., 2024) and used the similarity of the ICs to personality-descriptive terms to predict 35% variance of the Big Five dimension of openness in an independent test sample (N = 35). In sum, ICs can be used to define the individual semantic structures, reflecting personality and predicting individual reading behavior.

The Pupil Labs Neon for the study of face-to-face conversation: A test of data quality and comparison of AOI-analyses

Gijs Holleman[1], Thijs van Laarhoven[1], Jeroen Benjamins[2]
[1]Tilburg University; [2]Utrecht University
g.a.holleman@tilburguniversity.edu

Background and method. The Pupil Labs Neon is a calibration-free wearable eye-tracker that provides automatic fixation-classification and Area-of-Interest (AOI) analysis in Pupil Cloud. We assessed the PL-Neon's data quality by computing accuracy and precision measures using GlassesValidator (Niehorster et al., 2023). In addition, we compared the automatic AOI analysis in Pupil Cloud (FaceMapper) with manual AOI-analysis in GazeCode (Benjamins et al., 2018). Gaze of 36 subjects (participants=12, research team=24) was measured during face-to-face conversations with a PL-Neon and a calibration-validation procedure was conducted. For 10 participants, we compared proportion total fixation time on faces using FaceMapper with the same measure classified and manually annotated in GazeCode.
Results. Participants had average lower accuracy and higher precision (2.01°; RMS=0.29°) than gaze recordings of the research team (1.90°; RMS=0.36°). Average proportion total fixation time on faces was 0.54 (0.26–0.82) for the automatic face-AOI analysis (FaceMapper) and 0.40 (0.18–0.71) for manual face-AOI annotation (GazeCode).
Discussion. The obtained accuracy and precision values corresponded well with the Pupil Labs technical report (Baumann & Dierkes, 2023). We stress the importance of data quality for wearable eye-tracking studies with the PL-Neon and discuss trade-offs between automatic and manual methods for fixation-

The Effects of Lexical and Sentence Level Contextual Cues on Chinese Word Segmentation

Linjieqiong Huang[1], Xingshan Li[1,2]
[1]CAS Key Laboratory of Behavioral Science, Institute of Psychology, Chinese Academy of Sciences, Beijing, China; [2]Department of Psychology, University of Chinese Academy of Sciences, Beijing, China;
huangljq@psych.ac.cn

Unlike alphabetic languages, Chinese lacks inter-word spaces to mark word boundaries, sometimes leading to ambiguity in word boundaries, such as overlapping ambiguous strings (OASs). An OAS consists of three characters (ABC), where the middle character can form distinct words with the characters on both its left (word AB) and its right (word BC). To determine the correct segmentation structure, Chinese readers rely on context information. Our eye-movement study investigated the impact of different types of prior context on Chinese word segmentation. We manipulated the lexical- and sentence-level contextual cues orthogonally. Regarding the lexical-level contextual cue, the pre-OAS context included either a semantically related word to AB (i.e., semantically related condition) or an unrelated control word (i.e., semantically unrelated condition). For the sentence-level contextual cue, the high-constraint context exclusively favored the A-BC structure, whereas the low-constraint context imposed no constraints on segmentation structure. Results showed that in the low-constraint context, the semantically related condition exhibited lower regression-out probabilities than the unrelated condition. However, in the high-constraint context, the semantically related condition exhibited higher regression-out probabilities than the unrelated condition. These findings suggest that Chinese readers utilize the lexical-level contextual cue earlier or simultaneously with the sentence-level contextual cue during word segmentation.

Eye movement to measure the effect of syntactic complexity and socioeconomic status on school textbooks processing

Romualdo Ibáñez
Pontificia Universidad Católica de Valparaíso, Chile
romualdo.ibanez@pucv.cl

Given the relevance of school textbooks (ST), it's essential to know if students comprehend and consequently learn from this instructional material. In this context, understanding the different linguistic features that could impact the process becomes crucial. Among these linguistic features, syntactic complexity (SC) has demonstrated to affect the processing of written texts by secondary school students (Kleijn, 2018). Simultaneously, it has been evidenced that the effect of text characteristics interacts with individual student features, such as their socioeconomic status (SES) (Ibáñez, Moncada & Santana, 2022). In this study, we assessed the effect of SC on the processing and comprehension of school texts by 113 elementary school students. To achieve this, a 2x2 design was employed, integrating both within-subject and between-subject factors. Experimental materials were constructed from school texts. Data collection was conducted using the Portable Duo Eye Tracking equipment. The results demonstrated the interaction between SC and SES effects in certain areas of interest (Complete Area, Subject-Verb, Verb-Subject) with specific Eye-tracking measures (Total Fixation Duration and First Pass). From these preliminary findings, it can be argued that the effect of SC varies depending on students' SES, with those from lower SES being more affected.

How do first and second language readers deploy theory of mind during text processing? A cross-study comparison of mentalizing inferences and irony processing.

Antonio Iniesta[1], Karla Tarin[1], Vegas Hodgins[1], Chaimaa El Mouslih[1], Mehrgol Tiv[2], Debra Titone[1]
[1]McGill University, Canada; [2]US Census Bureau, United States of America
antonio.martineziniesta@mcgill.ca

Addressing questions about how general cognitive capacities support theory of mind in natural reading is crucial for understanding the relationship between social cognition and language. However, this endeavor is impeded by the fragmentation of relevant work across siloed subfields (e.g., inference generation, pragmatic language), for which cross-study data are rarely evaluated. To fill this gap, we integrated data from two independent studies (Hodgins et al., submitted; Tarin et al, submitted) that investigated mentalizing inferences and irony comprehension during natural reading for the same 53 bilingual adults (25 English-French, 28 French-English). Results showed that eye movements during ironic compliment reading related to early-stage (first pass time) but not late-stage (total reading time) measures of mentalizing inference generation for both L1 and L2 readers. Crucially, cognitive control measured by the AX-Continuous Performance task linked reactive control to enhanced L1-irony comprehension and proactive control to improved L2-irony comprehension. These effects did not extend to mentalizing. This collectively suggests that while the reading/cognitive processes involved in irony and inference comprehension partially overlap (i.e., initial stages of lexical access), there are significant differences (i.e., post-lexical access/semantic integration). These differences may arise from the distinct prediction and revision requirements of the situation model constructed during reading.

A framework for gaze-analysis during multimodal interactions in spatial environments

Sven Janßen, Thies Pfeiffer
Hochschule Emden Leer, Germany
sven.janssen@hs-emden-leer.de

The presented work is motivated by research on behavior analysis using machine learning during the process of requirement analysis with experts inspecting virtual prototypes of future products. During this process, spoken language, gestures and gazes on the products, as well as data about the 3D environment the experts are interacting with are recorded and analyzed in a multi-step processing pipeline including spatial re-simulations. Data management and handling are important methodological challenges here, as data from multiple sensors with different, yet high framerates need to be stored, integrated, and iteratively refined, while maintaining consistency and traceability throughout a complex analysis pipeline. We assessed and evaluated several representation formats with benchmarks in a photorealistic virtual reality set-up with a Varjo XR-3 headset, and developed data schemas for multimodal recordings in spatial environments including data from eye tracking, hand tracking, motion tracking, as well as speech recording and transcription. The results demonstrate how the hdf5 format can be used to efficiently store and analyze behavioral data of spatial interactions. The developed representation scheme and data processing pipeline allows researchers, e.g., to analyze speech and gesture interactions with joint gaze in 3D environments in an efficient manner and supports the replication of data-driven experiments.

EYEdentify - Investigating Unique Eye Gaze Patterns in Adults with Autism Spectrum Disorders Using Virtual Reality

Alberte Cathrine Ehrhardt Jeppesen[1,2], Johannes Andresen[1,2], Lars Clemmensen[1], Rizwan Parvaiz[3], Jens Richardt Møllegaard Jepsen[4], Louise Birkedal Glenthøj[1,2]
[1]VIRTU research Group, Copenhagen Research Center on Mental Health (CORE), Copenhagen University Hospital, Denmark; [2]University of Copenhagen, Denmark; [3]Department of ADHD and Autism, Mental Health Services, Capital Region of Denmark, Denmark; [4]Copenhagen Research Centre for Mental Health (CORE), Mental Health Centre Copenhagen, Copenhagen University Hospital
alberte.cathrine.ehrhardt.jeppesen@regionh.dk

Introduction: Individuals with autism spectrum disorders (ASD) often display distinct eye gaze patterns during social interactions, potentially serving as characteristic identifiers for this group. These gaze patterns are typically tracked through eye-tracking devices linked to computer screens exhibiting diverse stimuli. A novel avenue that holds significant promise is virtual reality (VR), which offers several advantages, including adaptable environments and enhanced control. In this study, our objective is to examine eye gaze patterns of adults with ASD within virtual social scenarios. Specifically, we intend to ascertain if VR-based eye gaze patterns can effectively differentiate between individuals with ASD and their neurotypical counterparts.
Methods: Constituting a case-control design, this study includes 140 participants with ASD and a matched control group of 50 neurotypical individuals with no known psychiatric conditions. The VR paradigm consists of 6 computer-generated social scenarios mimicking a pedestrian street, with increasing degrees of social complexity, and non-social distractors. Between groups analyses will be conducted on parameters including number of fixations, mean fixation time, and dwell time.
Results and discussion: The study is presently underway. Positive outcomes from this investigation have the potential to significantly contribute to expediting and objectifying ASD screening procedures.

The effect of white noise stimulation on oculomotor control in children with ADHD

Erica Jostrup[1], Emma Claesdotter-Knutsson[1,2], Pia Tallberg[1,2], Göran Söderlund[3,4], Peik Gustafsson[1], Marcus Nyström[5]
[1]Lund University, Lund, Sweden; [2]Region Skåne, Lund, Sweden; [3]Western Norway University of Applied Sciences, Sogndal, Norway; [4]University of Gothenburg, Gothenburg, Sweden; [5]Lund University Humanities Lab, Lund, Sweden
erica.jostrup@med.lu.se

In attention-deficit/hyperactivity disorder (ADHD), deficient inhibitory control is a prominent feature, and impairments in oculomotor control have been suggested as a potential biomarker of the disorder. While white noise stimulation has been demonstrated to enhance working memory performance, particularly in individuals with ADHD, its impact on oculomotor control remains unexplored.
This study investigated the effects of auditory and visual white pixel noise stimulation on oculomotor control in children diagnosed with ADHD and typically developing children (TDC). Memory Guided Saccades (MGS) and Prolonged Fixations (PF) are two tasks that have been suggested to have the highest sensitivity in detecting oculomotor deficiencies in ADHD. The tasks were performed during two levels of visual white pixel noise (25% and 50%), one level of auditory white noise (78dB) and a no noise condition.
Results revealed no effects of white noise stimulation on oculomotor control in either group. Results also question the effectiveness of the PF task in differentiating between the groups regarding oculomotor control.
We conclude that the previously reported beneficial effects of white noise stimulation on performance in children with ADHD do not seem to generalize to oculomotor control.

The role of personal traits in individual eye movement strategies during facial expression recognition

Sofya Klumova, Galina Menshikova
Moscow State University, Russian Federation
s.sofokl@mail.ru

The purpose of this study was to differentiate repeatable eye movement patterns during facial expression recognition and their correlation with personal traits. The hypothesis about the existence of individual invariant cyclic patterns of eye movements associated with personality traits was put forward. 75 subjects were presented photographs of people's faces expressing 7 basic expressions. A total of 210 stimuli were presented. Eye movements were recorded using SMI iViewX RED-500. Afterwards, subjects completed questionnaires. BeGaze, SPSS Statistics were used to analyze the data. The dependent variables were facial viewing routes among first 3 fixations ("strategy") and the fixation duration in areas of interest. To differentiate individual strategies, the zone of first fixation and strategy invariance were examined. It turned out that the zone of first fixation is a stable feature that allows us to divide the subjects into two groups - those who start their examination with the eye area and nose area. Further analysis allowed us to divide the subjects into two groups, people who use 1 strategy for the entire set and people with variable strategies. Significant differences between groups and correlations between the level of social anxiety and the fixation duration in the eye area were shown.

Testing the familiarity check assumption of E-Z Reader using concurrent eye-tracking and magnetoencephalography

Roslyn Wong[1], Judy Zhu[1], Lili Yu[1], Aaron Veldre[2], Iain Giblin[1], Paul Sowman[3], Erik D. Reichle[1]
[1]Macquarie University; [2]University of Technology Sydney; [3]Auckland University of Technology;
roslyn.wong@mq.edu.au

According to the E-Z Reader model of eye-movement control, the decision about when to move the eyes during reading depends on the completion of an early stage of lexical processing: the familiarity check. The present study used concurrent eye-tracking and magnetoencephalography (MEG) to identify the neural correlates of lexical processing that occur prior to eye movements and that predict when the eyes move from one word to another. Participants made lexical decisions about pairs of letter strings, with one displayed centrally and the other displayed randomly in the left or right periphery, while their brain activity was recorded by MEG. The frequency of centrally displayed words was manipulated. The results were analysed by aligning the MEG data to saccade onset from centrally displayed words and then examining the time course of brain activity modulated by word frequency prior to this decision to move the eyes, from early visual encoding through word identification and saccadic programming. The implications for the neural underpinnings of the eye-mind link in reading will be discussed.

Inhibition of return and saccadic momentum in a saccade sequencing paradigm

Christof Körner[1], Margit Höfler[1], Tim Hohensinn[1], Iain D. Gilchrist[2]
[1]University of Graz, Austria; [2]University of Bristol, UK
christof.koerner@uni-graz.at

We checked for the presence of saccadic inhibition of return and of saccadic momentum in an extended saccade sequencing paradigm, originally developed by Ludwig et al. (2009). Participants made saccades in a display that consisted of 16 (Experiment 1) or 25 circles (Experiment 2) as possible fixation locations. The gaze of the participants was guided either by a cue appearing in the circle to be fixated next (Experiment 1) or by highlighting that circle (Experiment 2). In Experiment 1, direction of the subsequent saccade deviated randomly from the preceding saccade direction. After six fixations, gaze was guided back to a circle fixated up to five fixations earlier (fixation lag). We found longer saccade latencies for fixation lags of up to three fixations back compared to fixations of non-fixated circles, indicating a robust effect of inhibition of return. Forward saccades were initiated faster than those with greater angular deviations, indicating saccadic momentum also. In Experiment 2, we varied saccade direction systematically. We found a linear increase of saccade latency with increasing angular deviation. The magnitude of this effect suggests that saccade latency in this paradigm is not exclusively determined by saccadic momentum but that both inhibition and momentum exert an influence.

Using eye movements for the identification of partners in dyadic interactions

David-Levente Kovacs
IT University of Copenhagen, Denmark
dako@itu.dk

The study investigated privacy concerns surrounding eye tracking technology within the context of dyadic interactions. We examined eye tracking data collected through two experiments involving dyadic interactions. In the first experiment participants either maintained eye contact directly, or through a mirror, with a baseline condition also included where no eye contact was present. In the second experiment a new condition was introduced where participants also had to shake hands while maintaining eye contact. We anonymized data from half of the participants (test set) and trained a Support Vector Classifier on the labeled data from the remaining participants. The classifier's objective was to match people in the labeled group with their partners in the unlabeled group, based solely on eye movement features (fixation duration, saccade velocity, blink rate, etc.) and their synchronization between partners. Our findings revealed a concerning level of accuracy in partner identification. Overall, our findings highlight the significant privacy risks inherent to eye tracking technology in social contexts. This research emphasizes the urgent need for robust, real-time anonymization techniques that safeguard eye tracking data from the moment of collection. Further research must explore methods to effectively protect user privacy while still enabling valuable applications of this technology.

The influence of pursuit eye movements on changes in brain activity during optokinetic exposure

Artem Kovalev, Ekaterina Nefeld
Lomonossov Moscow State Univeristy, Russian Federation
artem.kovalev.msu@mail.ru

Oculomotor activity is a part of the system for determining the position and orientation of the human body in space. The purpose of this study was to examine the effect of pursuit eye movements on changes in brain activity during optokinetic stimulation. 10 subjects participated in the study. The optokinetic virtual drum rotating in different directions around a vertical axis at speeds of 30 and 60 deg/s, painted in alternating black and white stripes, was presented in the HTC Vive Pro Eye. Two instructions were used – to fix the gaze in the center of the scene, to follow the movement of the stripes. The efficiency was monitored using a helmet eytracker (120 Hz). Brain activity was recorded using 24-channels functional infrared spectroscopy Artinis Brite. It was found that during pursuit eye movements there was a significant decrease in the level of blood oxygenation ($t=2.975$, $p=0.018$) in the occipital zone. Also, a decrease in oxygenation in the parietal zone was observed at a lower rotation speed only with the gaze fixation instruction ($t=2.412$, $p=0.039$). Thus, eye movements can be an element of a control system for the stable perception of the body position. This work was supported by grant RSF 23-78-10090.

Exploring Natural Sceneries: A Comparative Eye-Tracking Study of Freely Moving Participants in Virtual and Real Environments

Alexander Kreß[1], Frank Bremmer[1], Markus Lappe[2]
[1]Philipps Universität Marburg, Germany; [2]Universität Münster, Germany
alexander.kress@physik.uni-marburg.de

Capturing eye-tracking data in dynamic natural scenes presents significant challenges due to the necessity of maintaining consistent environmental conditions across participants. This study explores the potential of virtual environments to replicate real-world settings for investigating gaze behavior in freely moving individuals in natural environments. Utilizing a virtual reality (VR) motion platform that mimics natural walking, we recorded eye position data from participants navigating through five distinct virtual landscapes designed to mirror various real-world scenes, plus one control condition. A major focus of this setup was cost efficiency, aiming to provide a viable alternative to the more expensive and expansive 2D treadmill systems typically used. Participants engaged in a search task within these environments. Our analysis reveals that, although general gaze statistics remained consistent, notable differences in gaze behavior emerged. Specifically, in real-world settings, there is a pronounced increase in the downward gaze angle corresponding to terrain ruggedness, a correlation that is substantially attenuated in virtual conditions. These findings suggest that while virtual environments can effectively model real-world scenarios to some extent, discrepancies in specific gaze responses highlight the limitations of this approach. Thus, virtual environments offer a viable, though not perfect, alternative for simulating real-world conditions in eye-tracking research.

The role of vocabulary size and contextual diversity on word learning during sentence reading

Oliwia Kus, Ascension Pagan
University of Leicester, United Kingdom
ok65@student.le.ac.uk

A critical aspect for reading development is the acquisition of new vocabulary. Recently, some studies have begun to identify factors that facilitate word learning during silent sentence reading, using eye movement behaviour as an index of word learning. For example, Pagán and Nation (2019) showed that contextual diversity facilitated word learning during sentence reading. Additionally, although larger vocabulary size could be associated with effective learning (e.g., Stanovich et al., 1989); it is unclear if it can impact word learning. In this study, 55 adults were assessed on vocabulary knowledge and read novel words embedded in sentences while their eye movements were recorded. During the learning phase, novel words were presented either in the same sentence repeated four times or in four different sentences. During the pre- and post-learning phases, novel words were embedded in neutral sentences, and the invisible boundary paradigm was used (Rayner, 1975). For each novel word (e.g., faddle), a valid (e.g., faddle) and an invalid (e.g., hobbta) preview were created. Preliminary results showed a) the contextual diversity effect, b) the orthographic preview benefit was greater after learning and c) vocabulary size facilitated word learning. These findings provide novel insights into the link between word learning and memory.

Return Sweeps in Serial Naming Tasks: What does matter

Laoura Ziaka[1,2], Athanassios Protopapas[1]
[1]University of Oslo, Norway; [2]Oslo University Hospital, Norway
laoura.ziaka@gmail.com

In text reading, return sweeps (RSs) are saccades during line transitioning. After RSs, undersweep-fixations (fixations between an undershoot and a corrective saccade) may occur, usually landing on a row's second word. During these fixations, preprocessing of the fixated word and parafoveal processing of the first word is possible. Although RSs have received attention in reading tasks, less is known for serial naming tasks, leading to the rule of thumb of excluding the first and last item of all rows. Here, we take a step toward this direction by using two multi-item Stroop tasks differing in naming direction: the left-to-right (language default; LR) and the top-to-bottom (TB) task. Preliminary analysis of 42 Greek adults indicates that after undersweep-fixations, gaze duration on the first item is shorter for the LR task only. Moreover, a Stroop effect is evident in early and late measures of the LR task for both the first and second items, whereas it is observed in one late measure for the first item of the TB task. Our results indicate that processing after RSs is a dynamic interplay between automaticity, naming direction, and, hence, parafoveal processing, even suggesting that the second item is the most troublesome, not the first.

Wednesday afternoon – Poster Session 2

Investigating Brain Seizure Activity Detection in Epilepsy: Insights from EEG Analysis of Seven Individuals

Eyad Attar
King Abdulaziz Unversity, Saudi Arabia
etattar@kau.edu.sa

Epilepsy, a prevalent neurological disorder affecting approximately 50 million individuals globally, is characterized by recurrent seizures, transient disruptions in cerebral activity causing alterations in behavior and consciousness. Seizures are classified as focal or generalized based on the localization and extent of abnormal brain activity. Focal seizures manifest in specific brain areas, while generalized seizures involve widespread brain electrical activity, presenting symptoms like convulsions and loss of consciousness. This study examines seven individuals experiencing three to five seizures, aiming to detect brain seizure activity. EEG recordings from the Siena Scalp Database, accessible through PhysioNet, were analyzed using EEGLAB, a robust tool for EEG data processing. Independent Component Analysis (ICA) algorithms were employed to separate arterial EEG sources from neuronal-generated EEG sources. The efficacy of ICA algorithms in discerning different EEG sources was demonstrated, shedding light on the intricate nature of brain seizure activity detection in epilepsy. The research contributes valuable insights into the mechanisms underlying epilepsy seizures and highlights the potential of EEG analysis in improving diagnosis and treatment strategies.

EOG-Based Ocular Angle Estimation Without Assuming Equal Vertical Ocular Angles

Nathaniel Barbara, Tracey A. Camilleri, Kenneth P. Camilleri
University of Malta, Malta
nathaniel.barbara@um.edu.mt

Electrooculography (EOG) signals can be processed to estimate the ocular angles (OAs), which refer to the separate orientation of the two ocular globes. One recent method (Barbara et al., 2020) for EOG-based OA estimation assumes that the vertical OAs of the two ocular globes are equal. This is undesirable in applications where estimation of the orientations of each ocular globe is required. If this assumption is relaxed and a conventional four-electrode setup is used, the average horizontal and vertical OA estimation errors across 10 subjects were 3.60±1.18° and 9.52±3.58° for the left ocular globe, and 3.09±0.96° and 3.14±1.13° for the right ocular globe, respectively. Notably, the vertical OA estimation error is high for the left ocular globe, above and below which no electrodes are attached. However, including electrodes superiorly and inferiorly to this eye, resulted in a statistically significantly reduced error of 3.07±1.04° ($p<0.05$). Furthermore, the left horizontal OA estimation error, and the right horizontal and vertical OA estimation errors, were also statistically significantly improved ($p<0.05$) to 3.13±1.07°, 2.94±0.97° and 2.63±0.79°, respectively. Thus, relaxing the equal vertical OA assumption and adding two vertically-aligned electrodes to the left eye, permits the reliable estimation of the orientation of each ocular globe.

Sub-lexical semantic decoding in incidental word learning during natural Chinese reading

Feifei Liang[1], Ying Xiang[1], Xuejun Bai[1], Simon P. Liversedge[2]
[1]Faculty of Psychology, Tianjin Normal University, China; [2]School of Psychology and Humanities, University of Central Lancashire, UK
feifeiliang_329@126.com

Chinese characters are often formed with two radicals, one phonetic and the other semantic. This property of Chinese characters raises an intriguing question regarding whether the semantic, sub-lexical, structure of characters may be used to form representational links between characters and their semantic meanings during novel word acquisition. In the present study, we report a large scale, eye movement experiment that addressed this issue. We constructed 16 semantic-phonetic pseudo-characters as novel target words. Half of the pseudo-characters were semantically transparent, and the other half were semantically opaque. Each novel word was embedded into 9 constraint sentences and we recorded 113 adult participants' eye movements as the novel words were learnt through natural reading. Participants made shorter fixations for semantically transparent novel words relative to opaque ones during the initial 4 exposures, this effect did not maintain to the subsequent 5 exposures, indicating a decline in reliance on sub-lexical semantic decoding over the course of word learning. When participants were grouped as faster and slower readers, a semantic transparency effect was apparent for faster, but not slower readers, indicating differential use of sub-lexical semantic information in the instantiation and development of novel lexical representations during Chinese word learning.

Using Fixated-Related Potentials to Investigate Prediction Error during Natural Reading

Rupali Limachya[1,2], Steven Frisson[1], Federica Degno[3], Simon P. Liversedge[4], Kevin B. Paterson[2], Ascensión Pagán[2]
[1]School of Psychology, University of Birmingham, United Kingdom; [2]School of Psychology and Vision Sciences, University of Leicester, United Kingdom; [3]School of Psychology, Bournemouth University, United Kingdom; [4]School of Psychology, University of Central Lancashire, United Kingdom;
rxl025@student.bham.ac.uk

Current theories emphasise the importance of prediction as a mechanism for facilitating efficient word recognition during reading (Pickering & Gambi, 2018). An important assumption of such theories is that readers should experience a processing cost when they predict a word incorrectly (Cevoli et al., 2022). However, while such an effect is claimed to occur in post-N400 ERP components (Federmeirer et al., 2007), evidence for such effects in eye movement studies is lacking (Frisson et al., 2017; Luke & Christianson, 2016). We investigated this issue further, using a larger item set and EEG-eyetracking co-registration to establish if a prediction error cost can be obtained in either fixation times or fixation-related potentials (FRPs) for words during reading. Participants read sentences containing either a high or low predictable word in either strongly constraining or neutral contexts. Eye movement data show clear effects of sentential constraint and word predictability, but no evidence of a prediction error cost. FRP data are currently being analysed to determine whether they are in line with these eye movement data or similar to previous ERP data. We will also report data from older adult readers (60-80 y.o.) who participated in the same co-registration experiment.

Unpacking the relation between morphological awareness and word processing during sentence reading in Chinese children

Nina Liu, Yawen Gao, Qiancheng Gao, Guoli Yan
Faculty of psychology, Tianjin Normal University, China
lnnair@163.com

Morphological awareness (MA) plays a crucial role in the development of Chinese reading. This study investigated the impact of MA on word processing during sentence reading among Chinese children. We assessed 123 primary school children (61 in Grade3; 62 in Grade5) using a comprehensive test battery and recorded their eye movements while they read sentences embedding target words (two- and three-character words). Results indicated that MA exerted a unique predictive influence on gaze duration when controlling other reading skills. However, this influence varied by grade, with specific components of MA playing differential roles. For Grade 3 students, all three components of MA (homophone, homograph, and compounding) significantly predicted gaze duration independently. In contrast, for Grade 5 students, only compound awareness was a significant predictor, while homophone and homograph awareness did not significantly affect gaze duration. Moreover, the influence of compounding awareness on gaze duration was stronger in grade 5 than in grade 3. Additionally, quantile regression analysis showed that the predictive influence of MA on gaze duration strengthened as children's gaze duration lengthened. Results demonstrate MA is uniquely important for word processing during sentence reading, as well as revealing a developmental shift in its relationship with word processing in Chinese.

Effects of spatially congruent and incongruent sounds on visually-driven microsaccade direction modulations during primary visual cortex inactivation

Tatiana Malevich[1,2,3], Matthias P. Baumann[1,2,3], Yue Yu[1,2,3], Tong Zhang[1,2,3], Ziad M. Hafed[1,2,3]
[1]Hertie Institute for Clinical Brain Research, Tuebingen, Germany; [2]Centre for Integrative Neuroscience, Tuebingen, Germany; [3]University of Tuebingen, Tuebingen, Germany;
tatiana.malevich@cin.uni-tuebingen.de

In our recent work, we discovered that primary visual cortex (V1) inactivation abolishes the reflexive phenomenon of saccadic inhibition, in which visual cues cause robust changes in both saccade times and directions. Interestingly, when we paired visual stimuli with spatially uninformative sounds, both the inhibition and direction modulations were partially restored, suggesting a boosting by the sounds of latent visual signals bypassing V1. Here, we asked whether such boosting can be rendered even more effective if the sounds were spatially informative. In one macaque monkey, we presented a visual onset during fixation, either in the scotoma caused by V1 inactivation or in the opposite hemifield. In one block of trials, a lateralized speaker was placed either congruent or incongruent with the scotoma hemifield. When the sound was congruent, a visual onset in the blind hemifield was associated with a much stronger visual direction biasing of saccades than if the sound was incongruent. Importantly, even with incongruent sound, a visual stimulus in the scotoma still caused saccade biasing towards the visual stimulus. Thus, these results confirm the presence of latent visual signals bypassing V1 that can still robustly influence saccade direction dynamics during saccadic inhibition, especially when boosted by multi-sensory information.

Eye Movement Indicators of Mind-Wandering during Reading: A Meta-Analysis

Diane C. Mézière, **Niilo Hautala, Timo T. Heikkilä, Johanna K. Kaakinen**
University of Turku, Finland
diane.meziere@utu.fi

Mind-wandering has been extensively investigated in reading research, with several studies reporting differences in eye-movement behaviour compared to on-task reading. However, it remains unclear which eye-tracking measures are the most useful for catching mind-wandering episodes during reading. In this meta-analysis, we aim to identify eye movement indicators of mind-wandering during reading. We searched four databases (PsychInfo, PubMed, Web of Science, and Scopus) for articles within our inclusion criteria for search terms specifying the topic (e.g., mind-wandering, task-unrelated thoughts), measures (e.g., eye movements), and task (i.e., reading). Abstracts from 140 articles were reviewed for eligibility, and 39 articles were included for full text reading and data extraction. Finally, we identified 16 individual datasets from 19 articles for which we could compute effect sizes. We calculated effect sizes for 9 eye-movement measures: mean fixation duration, fixations count, first-fixation duration, gaze duration, total reading time, saccade length, skipping, blink count, and inter-word regressions, and. The number of effect sizes that could be computed for each measure ranged from 3 to 8. The results indicated that readers skipped more words during mind-wandering compared to on-task reading. None of the other measures showed significant effects.

How reading on a computer affects comprehension in college-aged readers: An eye movement investigation

Wiralpach Nawabutsitthirat, Barbara Juhasz
Wesleyan University, United States of America
bjuhasz@wesleyan.edu

Reading texts on a computer has become increasingly common in secondary education. Yet, previous research suggests that comprehension is worse when college students read on a computer (e.g. Singer & Alexander, 2017). The current study sought to clarify the differences in reading times and comprehension when college-level informational texts are read on a computer screen compared to on paper. Four passages were selected from an open-source Psychology textbook. Each passage contained a short title and a 200-word paragraph. Forty-eight college students read two passages on a computer screen while their eye movements were recorded. They then read two passages on paper. Comprehension was also assessed. In addition, titles and passages were written in a contrasting serif (Lucida Bright) and sans serif font (Lucida Sans) to examine the potential impact of font style on reading. Students read the passages significantly faster on the computer screen relative to paper. This increase in reading speed was at the cost of comprehension, which was significantly worse when reading on a computer. There was also a subtle effect of font type, with participants making significantly more fixations when the body of the text was presented in a sans serif compared to a serif font.

gazeMapper: A tool for automated world-based analysis of wearable eye tracker data

Diederick C. Niehorster[1], Roy S. Hessels[2], Marcus Nyström[1], Jeroen S. Benjamins[2], Ignace T. C. Hooge[2]
[1]Lund University, Sweden; [2]Utrecht University, The Netherlands
diederick_c.niehorster@humlab.lu.se

In comparison to young adult readers (18-35 years), older readers (65+ years) make longer saccades, longer and more frequent fixations, more regressions, and more word skips. A key theoretical account attributes this to a "risky" reading strategy, in which older readers attempt to guess the identities of upcoming words. However, it is not yet known whether this pattern holds across older adulthood. Indeed, studies have previously focused on the "younger-old" (65-75 years), and so we have little understanding of reading in the "older-old" (80+ years). Previous research into the component processes underlying reading has indicated that age-related changes do not always follow a linear trajectory. For example, sensory abilities decline increasingly rapidly with advancing age, whereas vocabulary knowledge appears to increase until young-older age, and then decline in older-old age. Thus, the cognitive mechanisms underlying reading may differ markedly between younger-old and older-old adults. Accordingly, the eye movements of young, younger-old, and older-old participants will be monitored as they read sentences. The findings will provide crucial insights into how the processes underlying reading change across older adulthood, as well as highlighting the methodological challenges involved with researching older populations.

.

Target size modulates smooth pursuit gain in patients with schizophrenia spectrum disorder

Rozana Ovsepian[1], Frank Bremmer[2,3], Benjamin Straube[3,4], Tilo Kircher[3,4], Alexander C. Schütz[1,3]
[1]Department of Psychology, University of Marburg; [2]Department of Physics, University of Marburg; [3]Center for Mind Brain and Behavior, Universities of Marburg, Giessen and Darmstadt; [4]Department of Psychiatry and Psychotherapy, University of Marburg
ovsepian@staff.uni-marburg.de

In schizophrenia, abnormal smooth pursuit eye movements, characterized by reduced smooth pursuit gain and increased frequency of catch-up saccades, is one of the most replicated dysfunctions under laboratory conditions. In healthy subjects, the occurrence of catch-up saccades depends on characteristics of the pursuit target: Findings reveal fewer saccades with dot configurations lacking a central target, while pursuit gain and latency remain consistent across different dot configurations. In the present study, we aimed to characterize the ocular pursuit performance of schizophrenia patients to single-dot and multi-dot stimuli with or without central target. Consistent with previous findings, a multi-dot stimulus without a central target produced fewer saccades than a single-dot stimulus or a multi-dot stimulus with a central target. In contrast, during either the initial open-loop or steady-state phase, the mean pursuit gain was significantly increased when tracking the multi-dot stimuli compared to the single-dot stimulus. This indicates that this group of patients might specifically benefit from larger pursuit targets. Larger eye movement targets might facilitate motion processing or might provide a stronger signal to drive pursuit eye movements in patients with schizophrenia spectrum disorder.

Eye movements in biliterate children with and without dyslexia in reading English and Kannada

Dr. Aparna Pandey[1], Dr. Prakash Padakannaya[2]
[1]IIT Bhubaneswar, India; [2]Christ (deemed to be University)
aparnapandey@iitbbs.ac.in

Progressive power lenses (PPL) provide the presbyope with sharp vision from far to near distances, despite causing astigmatism outside their central region. Different designs of PPL vary in power and aberration distribution, impacting visual performance for a given task in different ways. Eye-Trackers (ET) provide useful information about visual quality by means of ocular movement metrics like fixation stability, which is measured by bivariate contour ellipse area (BCEA). Tobii Pro Glasses 3 were used to record pupil position of 20 subjects wearing two PPL designs, EndlessSteadyNear and Inmotion (IOT), a specific design for dynamic tasks like driving. They performed 4 dynamic tasks: (1) fixation of a letter which appears at a random position out of 9 possibilities, (2) same task than (1) but 2 possible positions, (3) descending and (4) ascending stairs. BCEA was calculated for each subject and condition and differences between designs were analyzed with a t-test. Averaged BCEA for the four tasks were: (1)0.20±0.17deg2 & 0.15±0.16deg2 for EndlessSteadyNear and InMotion respectively, (2)0.34±0.54deg2 & 0.11±0.10deg2, (3)0.26±0.21deg2 & 0.17±0.08deg2, and (4)0.18±0.07deg2 & 0.17±0.12deg2. Statistically significative differences between designs were found (p-value=0.04). In conclusion, both designs present good performance during dynamic tasks, but fixation stability is higher with InMotion lens.

Can Local Meaning Predict Task-Dependent Fixation Patterns?

Thore Pingpank, Franz Faul, Antje Nuthmann
Kiel University, Germany
pingpank@psychologie.uni-kiel.de

Meaning maps serve as a tool to represent the spatial distribution of semantic features in scenes. Local meaning has been identified as a reliable predictor of fixations across non-specific viewing tasks, including scene memorisation and free viewing. However, it is unclear how local meaning performs when viewers are required to make informed judgements about specific properties of the scene or the scenes' context. To test the tacit assumption that meaning can be measured independently of the viewer's intentions, we investigated how well meaning maps predict fixation patterns in different tasks. Participants viewed 67 indoor scenes under five instructions related to the occupant, objective characteristics of the room (e.g., its cleanliness) and the comfort it provides. For each scene, a contextualised meaning map was created by aggregating ratings on the meaningfulness of scene patches. We tested the hypotheses that a) the fixation pattern varies between tasks, b) the predictive accuracy of meaning maps varies between tasks, and c) the quality of prediction changes over the course of the scene observation. Overall, the results indicate similarities across tasks that can potentially be explained by meaning maps, but also a task-dependent emphasis on certain objects that cannot be captured by local meaning.

An interplay between forward and backward saccade adaptation

Patrik Polgári, Alexander C. Schütz
Philipps-Universität Marburg, Germany
patrik.polgari.lfgeb@gmail.com

Adaptive changes in saccade amplitude are supported by two mechanisms, with forward adaptation being weaker and having a slower dynamic profile than backward adaptation. Furthermore, saccade adaptation operates in a direction-specific manner, such that adaptation for leftward saccades does not transfer to rightward saccades. However, most studies adapted only saccades in one direction starting from the same original eye position, which limits the ability to observe a potential interaction between the two mechanisms happening in different directions.
We tested the hypothesis that the learned saccade error in one direction can inform saccade adaptation in the opposite direction if the errors are consistent with a horizontal shift of the visual scene.
Using a horizontal random walk paradigm, we adapted participants' leftward and rightward saccades in three conditions: (1) backwards only, (2) forwards only, and (3) backwards in one direction and forwards in the other.
Preliminary results indicate stronger adaptation when saccades are adapted backward and forward in two opposite directions. This suggests an interplay between backward and forward saccade adaptation mechanisms when these adaptive changes in saccade amplitude occur concurrently in opposite directions.

Advancing dyslexia intervention with gaze-based interactions in DeveLex software

Stanislav Popelka[1,2], Michaela Vojtechovska[1,2], Nicol Dostalova[2], Cenek Sasinka[2]
[1]Palacký University Olomouc, Czech Republic; [2]Masaryk University, Brno, Czech Republic;
stanislav.popelka@upol.cz

DeveLex Software introduces an innovative prototype developed for dyslexia intervention through gaze-based interactions. This web-based application, crafted in Svelte and TypeScript, leverages eye-tracking technology not just as a user interface but also as a means of monitoring and adapting to the user's performance in real-time. Integrating a WebSocket data bridge facilitates the connection with remote eye-trackers, allowing for dynamic adjustment of task difficulty based on real-time performance data.
The software features interactive tasks designed to aid in dyslexia intervention, with the current prototype showcasing a paired reading task tested with the GazePoint GP 3 HD eye-tracker. This setup exemplifies the potential for eye-tracking technology to enhance educational software, particularly for dyslexia intervention, by automatically adjusting task parameters to sustain user engagement and challenge.
Future developments will expand the software's task library and integrate it more into existing educational ecosystems, with the goal of offering a personalized, adaptive learning experience. This approach aims to support the academic achievement and emotional well-being of students with dyslexia by providing targeted, efficient, and engaging intervention strategies. DeveLex software represents a promising step forward in the application of gaze-based interactions for dyslexia intervention, showcasing the potential for technology to facilitate personalized learning experiences.

Cookie cravings – Sugar content information affects Christmas treat preferences

Jonas Potthoff
University of Graz, Austria
jonas.potthoff@uni-graz.at

High-sugar diets can promote the development of overweight. Especially during the Holiday season, when high-sugar food is abundant, people overeat and gain more weight than during other times of the year. The present study investigated how sugar content information affects food preference and visual attention in a buffet-like situation. Fifty-eight participants well acquainted with the local Christmas traditions and foods were presented with four cookies and two non-foods (presents). Cookies were either 'Christmas cookies' (cookies traditionally eaten only at Christmas) or had no Christmas association and were either labeled as cookies made with or without sugar. Participants reported higher wanting and liking for cookies with sugar, particularly Christmas cookies (interaction effect for wanting: $p = .047$, $\eta_p^2 = .059$; interaction effect for liking: $p = .017$, $\eta_p^2 = .084$). Sugar-free cookies were fixated more often ($p = .028$; $d = 0.35$) and shorter ($p < .001$; $d = 0.64$) than sugar cookies. Being informed that cookies are sugar-free reduced the reported preference and was associated with a more detail-oriented viewing pattern. The study's findings can potentially aid in developing interventions to promote healthier food choices during festive periods. The new strategies should not focus on the sugar content of foods.

Eye movements as a potential mechanism for synchrony perception plasticity

Anna Ptukha
University of Helsinki, Finland
anna.ptukha@helsinki.fi

A delay of 350 ms between sound and visual components of a multimodal stimulus would correspond to a real stimulus positioned about 100 m away from the observer. In real-world settings, human observers easily adapt to such asynchrony and perceive a single multimodal stimulus. However, a number of psychophysical experiments showed that under specific conditions humans can perceive even a 20 ms delay between the auditory and visual signals.

In our experiments, the perception of multimodal events was studied in relation to fixational eye movements (FEMs). Human participants watched monochrome videos featuring a musician (either the hands of a pianist playing the keyboard, or a drummer). Two conditions were introduced – temporal synchrony and 350 ms asynchrony between the visual and audio streams. Luminance and contrast of stimuli were equalized in time, and short periods of random duration of each condition were interleaved. No history effects were observed. Most of participants could not consciously detect this temporal asynchrony. Their total FEMs duration was longer when viewing the same video in the asynchronous condition, and more fixational microsaccades had larger amplitudes. Thus, fixational eye movements can potentially serve as a plasticity mechanism in the perception of synchrony in multimodal stimuli.

An analysis of eye movements of novice and expert Wordle players

Ananya Rajora, Ronan Reilly
Maynooth University
ronan.reilly@mu.ie

This study explored problem-solving strategies among players of the game Wordle. This is a popular word puzzle in which players must guess a five-letter English word, while being given feedback about the accuracy of their guesses (i.e., whether their guess contained a letter in the target word and whether its location was correct). Participants comprised players of differing aptitude and their pattern of eye movements was studied as they played the game. By comparing eye movements of experts and novices, it was hoped to detect different playing strategies across levels of expertise. The study used SR-Research's WebLink software in conjunction with an EyeLink 1000 to monitor scanpaths through predefined areas of interest (e.g., the keyboard, previous rounds of guesses, the current round). Results demonstrated distinct patterns: experts showed a more concentrated visual focus along with strategic transitions in focus, while beginners displayed more extensive and exploratory eye movement patterns. Ultimately, the goal of the study is to see if providing guidance to novices based on the eye movement patterns of experts might benefit their performance, as has been observed in the Grant and Spivey (2003) study of attention guidance.

Experimental design of eye tracking based validation of customer requirements

Lisa Reintanz, Lena Stubbemann, Robert Refflinghaus
University of Kassel, Germany
lisa.reintanz@uni-kassel.de

Within the product development process, customer requirements are essential. But common tools for customer requirement analysis and validation involve high costs and time expenditure. Moreover, the common tools hardly ensure high objectivity. The research project therefore aims to validate customer requirements by combining eye tracking and speech data. Eye movements show the areas of interest within a product whereas speech data reveal the reason for someone looking at this area.
For the experimental design we firstly conduct a survey to learn about the probands preferences and to introduce a psychological priming to the product. Afterwards, the probands look at a virtual prototype and simultaneously speak about the product features. During this phase we collect eye tracking and speech data. Then we repeat the survey from the beginning. In the end all those data are combined to validate the requirements for the product features by using the probands verbal feedback. The data will be analysed by using a machine learning approach to improve the velocity of the whole process. This method will increase the richness and resilience of customer requirements analysis and validation.

Dynamics of Eye Movements during Schulte Tables Completion in Stressful Situations

Kseniia Ryseva[1], Artem Kovalev[2], Vladislav Pedashenko[2]
[1]Federal Scientific Centre of Psychological and Multidisciplinary Research, Russian Federation;
[2]Lomonosov Moscow State University, Russian Federation
rysevakm@my.msu.ru

Stressful circumstances have been shown to influence the way individuals perceive information, which might be represented in the oculomotor activity. In order to assess this phenomenon, this study was aimed at examining eye-movement during the completion of the Schulte tables. The study involved 19 participants (18.36 y.o, SD = 0.7) all of whom were female. Data was collected using the Eyelink 1000+ system, left eye registration at 500 Hz. Three Schulte tables were used in a specific sequence. The experimental group completed the tasks under the time deficit condition. The successful completion of the tasks was determined by the accuracy of the click sequence, which was quantified in terms of points earned. Additionally, we calculated the average dispersion of the total absolute change in speed (TACS) along two coordinates using the formulae: std (|x_t - x_{t-1}| + |y_t - y_{t-1}|). The results showed that TACS demonstrated a statistical significance for only the 1st Shulte table between the control and experimental group in t-test (t=2.25, p=.048). Thus, the acquired results demonstrate that in stressful situations the TACS is lower, the eye-movements are less rapid and have similar speed between fixations. This study was supported by grant RSF №23-78-10090

How much time is left? Effects of time pressure in visual search

Margit Höfler[1,2], Alejandro J. Cambronero Delgadillo[1], Sarah J. Nachtnebel[1], Iain D. Gilchrist[3], Christof Körner[1]
[1]University of Graz, Austria; [2]University for Continuing Education Krems, Austria;
[3]University of Bristol, UK
ma.hoefler@uni-graz.at

Time pressure has been shown to be disruptive for visual search tasks. Here we tested whether and to what extent uncertainty about the amount of time pressure affects visual search performance. In each trial, participants were asked to search two subsequently presented displays and report the orientation of the always-present T-shaped target among L-shaped distractors. In Expt. 1, both displays always consisted of 24 items (hard-hard searches); in Expt. 2, Display 1 consisted of 24 items while Display 2 consisted of 8 items (hard-easy searches) and in Expt. 3, hard-hard and hard-easy searches were intermixed within a block. To vary the level of uncertainty about the amount of time pressure, we compared intermixed and pure blocks of either high or low time-pressure trials in all experiments. Our results revealed that search accuracy decreased as time pressure increased and that participants usually prioritized completing the first search instead of moving to the next (easier) search. Preliminary findings also indicate that uncertainty about the amount of time pressure in the intermixed blocks led to a worse search performance than when time pressure was predictable, suggesting that uncertainty should be considered as an important factor when investigating time pressure and interruptions.

Speeding through the lines: Effects of reading speed on eye movement control and word processing

Laura Schwalm, Ralph Radach
University of Wuppertal, Germany
lschwalm@uni-wuppertal.de

We examined how eye movement control during reading can be adjusted to changes in reading speed. Five-sentence paragraphs were presented in grey font, with only one line highlighted in black from top to bottom at a predefined speed. This method allows for good experimental control of speed while preserving nearly natural reading.
As a baseline, we determined the individual reading speed of forty participants. The Line-by-Line technique was applied at 100% of the baseline speed, followed by 125% and then 150%. Materials consisted of 72 items, each containing five sentences (279 – 423 words).
Results indicate that at higher speeds fewer words were fixated. However, there was no change in first-pass reading times, as faster word reading was mainly achieved through a reduction in regressions and re-reading. The word frequency effect persisted across all speeds, with a smaller difference between high and low-frequency target words in total viewing time at higher reading speeds.
These results suggest that experienced readers have a substantial residual capacity that can be utilized when reading speed increases, allowing for maintained word processing and comprehension. At higher speeds the rate of re-reading is reduced, indicating that a more relaxed criterion for comprehension (monitoring) is adopted.

Webcam based eye-tracking -Validation Study Report

Divya Prakash Seernani, Morten Mosbaek Pedersen, Kerstin Wolf
iMotions A/S, Denmark
divya.seernani@imotions.com

The purpose of the validation study was to run a large-scale study to evaluate how the iMotions WebET 3.0 algorithm performs on a truly diverse, global, sample, "in-the-wild". Data was globally collected from 255 participants over 35 days. Participants conducted a short study comprising of gifs, images, videos, and surveys. Self-reported parameters for ethinicity, eye-color, wearing glasses or not, having facial hair or not, and self-reported lighting conditions in the room were evaluated against accuracy. Over 50% of participants had an accuracy of 2 degrees of visual angle (dva) or lower. Over 70% had an accuracy of 3dva or lower and over 90% had an accuracy of 5dva or lower. Of the parameters measured, only the presence of glasses had a significant effect on accuracy. Over time, fixation classification stays stable in the center of the screen but classification may reduce in accuracy towards the bottom corners of the screen. Longer studies and internet problems can cause problems with participant compliance and a suboptimal user experience. The individual differences for ethinicities, regions, eye-colour and the presence of facial hair did not have a significant impact as people collected data in their natural environments, indicating an unbiased dataset on a demographic level.

Perceptual span during reading in Russian

Vladislava Staroverova[1], Svetlana Alexeeva[2], Anastasia Sycheva[1], Anastasiya Lopukhina[3]
[1]HSE University, Russia; [2]Saint Petersburg State University, Russia; [3]Royal Holloway, University of London, United Kingdom
nastya.lopukhina@gmail.com

Studies with adult readers of English established that the amount of information a reader can perceive within one fixation – perceptual span – is 14-15 letter spaces to the right of fixation (Rayner, 2009). Our study explores whether these findings apply to reading in other alphabetic languages. In the two eye-tracking experiments, we measured the perceptual span during reading in Russian and compared the conditions in which the information about word length was preserved or removed. We tested 177 adults who read 80 sentences in the moving window paradigm with X-replacements, excluding or including space replacements. The sentences were presented either without replacements or with 12, 14, or 16 visible characters to the right of the fixation. The results indicate that the reading speed in all masked conditions was slower compared to the unmasked condition. Also, in masked conditions, when the spaces between words were left intact, the reading speed stopped increasing at 12 visible characters to the right. However, when the spaces were replaced with Xs, it stopped increasing at 14 characters. Altogether these findings showed that readers used parafoveal information about word length and that the perceptual span during reading in Russian might be wider compared to English.

The influence of the unselected meaning of homographs in reading Chinese sentences

Jie-Li Tsai
National Chengchi University, Taiwan
jltsai@nccu.edu.tw

The present study investigated the processes of Chinese homographs in lexical ambiguity resolution. The subordinate bias effect (SBE) found in previous research demonstrated the competition between dominate meaning and the context-facilitated subordinate meaning when selecting the appropriated meaning of homograph in sentence. It is unclear whether the activation of the unselected meaning would vanish or remain after meaning selection. One experiment manipulating the strength of the preceding context and meaning ambiguity of Chinese words was conducted. For homographs, the preceding context was biased to the subordinate meaning with different strength and the following context was biased to the dominated meaning. Sixty native Chinese speakers read 120 sentences while the eye movements were recorded. The results showed that fixation durations on homographs were longer than unambiguous words. The interaction was significant in the second-pass measures showing a larger SBE in weak context than in strong context. The post-target words showed the same interaction as target words. The findings suggest that the activation of dominate meaning competes with context-appropriated subordinate meaning during lexical access. Moreover, the unselected dominated meaning needs more time to be integrated with the following related context due to the cost of the meaning selection in lexical ambiguity resolution.

Enhancing Human Performance in Air Traffic Control using Eye Tracking Technology and Artificial Intelligence Support

Celina Vetter[1], Kristina Samardžić[2], Ivan Tukarić[2], Tomislav Radišić[2], Ruth Haeusler Hermann[1]
[1]Zurich University of Applied Sciences (Centre for Aviation); [2]University of Zagreb
(Faculty of Transport and Traffic Sciences)
vetn@zhaw.ch

The rise of air traffic leads to increased workload of air traffic controllers. As a result, there is a growing need for more automation and supporting tools to reduce workload without compromising the controllers' situational awareness (SA). Our study investigates (i) eye-tracking (ET) indicators suitable for objectively assessing SA levels of en-route air traffic controllers, and (ii) how ET data can assess human performance in conflict detection and resolution. ET data were collected from 20 licensed ATCOs over five air traffic scenarios of varying complexity in two simulator sessions. In the first simulator session, the ATCOs performed their tasks without assistance, while in the second session they received AI-based conflict detection support. Our results suggest that a combination of the ATCO's fixation duration on the aircraft involved in the conflicts and the frequency with which ATCOs looked at these aircraft, as well as scanning patterns with respect to conflict-involved aircraft combinations are good indicators of SA. Furthermore, we found that conflicts are recognised faster with AI support, but not resolved faster. Indeed, it plays an important role when the conflict information is delivered to the ATCOs.

The WHO said what? Interaction of source credibility and readers' prior beliefs in the reading of social media posts

Oskari J. Virtanen, Johanna K. Kaakinen
University of Turku, Finland
t09ovirt@utu.fi

This study examined the effects of source credibility and prior beliefs on reading in a social media context. Participants' (n = 83) beliefs on societally relevant topics were measured with an online questionnaire, after which they took part in an eye tracking experiment. Participants were shown 76 mock-up Twitter posts from either credible or noncredible sources. Tweets consisted of a target sentence (pro- or contra-claim), followed by a neutral spillover sentence. Separate interest areas were defined for sources (usernames) and both sentences. First-pass and look-back reading times, along with look-back probabilities (1/0), were calculated for target and spillover sentences. Source reading times (summed fixation duration after reading the target sentence) and look-back probabilities were calculated for source areas. Source reading times and source look-back probabilities were the highest when participants saw disagreeable claims from credible sources, and, to a lesser extent, agreeable claims from noncredible sources. Similar interactions were found for look-back reading times and look-back probabilities for target and spillover sentences, but not first-pass reading times. These results suggest that an incongruence between source credibility and (especially negative) prior beliefs increases processing effort. With more neutral beliefs, source credibility has little influence on reading.

Unveiling Religious Imagination Through AI and Eye-Tracking

Michaela Vojtechovska, Markéta Muczková, Nikola Svobodníková, Klára Kubálková, Kamila Fačevicová, Stanislav Popelka, Tomáš Bubík
Palacký University Olomouc, Czech Republic
mail@vojtechovska.com

Presenting a novel methodology, we combine artificial intelligence (AI) with eye-tracking to gain deeper insights into religious imagination. This approach examines how participants visualize spiritual ideas through AI-generated images, aiming to identify the religious concepts that significantly influence the individuals. Such concepts include anthropomorphism—the tendency to envision the supernatural in human-like forms, holism—the view of the divine as an interconnected essence pervading the universe, and others.
On 22 compositions, Czech participants answered questions such as "How do you imagine God?" by choosing from 12 AI-generated images in randomized order. Images are weighted (0-6) for each concept by experts in religious studies. Addressing AI's inherent cultural bias, scores are normalized based on each concept's minimum and maximum achievable points. Using a Tobii Pro Fusion eye-tracker, we analyze participants' choices, deliberation, interest, and return patterns to images through AOI-based metrics. Descriptive and inferential statistical analysis can reveal the influence of each concept on the imagination of different ideas between specific demographic and religious groups.
The participants' choices are visualized using on-the-fly generated radar charts comparing the influence of concepts. Our approach could pave the way for using AI and eye-tracking technology in cultural studies fields beyond religion science.
.

Eye-movement patterns of subclinical body dysmorphic individuals in social situations

Christian Vorstius[1], Lia Mengsteab[1], Ralph Radach[1], Katrin Schoenenberg[2]
[1]University of Wuppertal, Germany; [2]University of Kiel, Germany
vorstius@uni-wuppertal.de

Body dysmorphic disorder (BDD) and social anxiety disorder (SAD) share similarities in phenomenology and presumed underlying mechanisms (Coles et al., 2006; Fang & Hofmann, 2010; Kelly, Walters, & Phillips, 2010). For both disorders, models assume selective attention to threat stimuli and hypervigilance (Richards et al., 2014), both affecting visual attention, e.g., in terms of hyperscanning. However, empirical findings for BDD are lacking. Method: We tracked eye movements (EyeLink100, remote option) in a sample with subclinical BDD symptoms, while participants were engaged in a 4min speech in front of a video conference audience (Chen et al., 2015). Results: During their speech, scan path length (in degree of visual angle) of BDD individuals did not differ from healthy controls (HC), contrary to findings from SAD samples. Regarding the distribution of attention towards positive, threatening, or neutral facial expressions, a significant main effect of expression emerged for total viewing times, however, there was no interaction with group (BDD vs. HC). Finally, a trend towards longer total viewing times in areas outside of social cues (i.e., outside of the tiles depicting individuals) was found for BDD individuals. Conclusion: BDD and SAD appear to rely on slightly different underlying mechanisms, theoretical implications will be discussed.

Characterizing the Variability of Eye Movement Behaviours in Children Reading English: A Corpus Study

Kendall E. Walter[1], Hazel I. Blythe[2], Sara V. Milledge[1], David Thomson[3], Simon P. Liversedge[1]
[1]University of Central Lancashire; [2]Northumbria University; [3]Thomson Software Solutions;
kwalter@uclan.ac.uk

Children consistently demonstrate greater variability in their eye movements during reading compared to adults. We used an eye movement corpus from English readers to explore potential causes of this increased variance. Our analyses assessed group differences in offline reading assessment scores and eye movement measures. We also assessed between groups differences in relation to proportional effects. Results showed that children's reading scores reflected reading time measures based on eye movement data, but not those of adults, perhaps as a result of the nature of the stimuli (which were created for children). Additionally, there were clear group differences in respect of analyses based on absolute differences, however, proportional analyses resulted in much smaller between groups effects. The results indicate that consideration of proportionality in reading performance metrics between children and adults might reflect a greater degree of comparability in performance than has previously been assumed to be the case.

An Eye-Tracking Study to Understand the Connections Between Social Rank Information, Task Performance, Sleep and Mental Health

Gamze Kocdemir
Lancaster University, United Kingdom
g.kocdemir1@lancaster.ac.uk

Social comparisons among peers have a strong influence on an individual's task performance. Past research has shown that social comparisons to peers can facilitate or impair performance via an influence on attention and inhibitory control. Psychological research has also investigated how social comparisons are connected to poor sleep and mental health episodes in university students. This study aims to investigate how social rank information affects task performance in university students, and if this is related to poor sleep and mental health and wellbeing. This study has used self-reported measures of subjective social status, sleep quality, and mental health and collected data on sleep and wake activity for the prior 7 days from wrist worn actigraphy. We have used social rank manipulation and a facial antisaccade (AS) task to assess the influence of social rank information on inhibitory control. We predict impaired AS performance in response to higher ranking peers in students with lower subjective status and this will correlate with poorer sleep and mental health. This study will help us understand more about how social attention can be modulated by social comparisons and help inform psychological interventions for the poor sleep and mental health observed in undergraduate students.

Is There a Preferred Viewing Location during Chinese Reading? Novel evidence from Hong Kong Corpus (of Chinese Sentence and Passage Reading)

Yushu WU, Chunyu KIT
City University of Hong Kong, Hong Kong S.A.R. (China)
yushuwu2-c@my.cityu.edu.hk

This study investigates the preferred viewing location (PVL) of initial landing positions (ILPs) and provides new evidence for the existence of PVL in Chinese reading. Left-of-centre PVL in alphabetic scripts is well-documented, but its presence in non-alphabetic scripts is debated. Different from previous research, which concludes the PVL as artefacts (Li et al., 2011), the present research reveals a left-of-centre PVL in Chinese natural reading. By utilising HKC and eye-movement data, our study echoes the classic PVL for forward fixations within an English word. Additionally, language properties significantly modulate the curving of the PVL: (1) Words in sentence reading have more leftward PVLs from the word centre than those in passage reading; (2) More visually complex words or low-frequency words have more left-of-centre PVLs. We speculate that greater cognitive difficulty contributes to a more leftward deviation of the PVL, which supports a processing-based strategy for saccade targeting of Chinese readers.

Processing Code-Switched Words Interactively: An Eye-tracking Study

Michael C. W. Yip
The Education University of Hong Kong, Hong Kong S.A.R. (China)
mcwyip@eduhk.hk

This study examined the dynamic relationship among different lexical variables of spoken word recognition of code-switched words in context by an eye-tracking experiment. Eighty native Cantonese-English listeners were recruited to the experiment. In this experiment, listeners were asked to listen carefully to different Chinese-English code-mixed sentences and look at different words presented on the computer screen. The target code-switched words were manipulated to (a) Phonotactic structure of the code-switched word (CC vs. CV), (b) Language phonetics of the target word (code-switcher vs. borrower), (c) Context type (constrained vs. neutral vs. non-code-mixed) and (d) Probe type (related to the Chinese meaning of the target code-switched word, or the English meaning of the word, or a phonologically similar word to the target, or an unrelated control) in the experiment. Eye-movement data revealed that (1) sentence context had an early effect on the processes of the code-switched word recognition; (2) sentence context interacted with language phonetics of the code-switched word during lexical access; and (3) phonological information of the distracters only had a weak effect on the spoken word recognition processes. Finally, the patterns of eye-tracking results further support the interactive approach of spoken word recognition of Chinese and code-switched words in context.

Parafoveal processing of Chinese four-character idioms with symmetrical and asymmetrical structure

Chuanli Zang[1], Wei Lu[2], Zhichao Zhang[2], Simon P. Liversedge[1]
[1]University of Central Lancashire, United Kingdom; [2]Tianjin Normal University, China
CZang@uclan.ac.uk

Research has demonstrated that commonly used Chinese four-character idioms are represented and processed parafoveally as Multi-Constituent Units (MCUs, see Zang et al., 2024) even though they often extend rightward beyond the limits of the perceptual span during reading. Are idioms with different structures parafoveally processed differently? In symmetrical idioms like “**争分**夺秒”, both “**争分**” (seize every minute) and “夺秒” (compete for every second) the constituents have a similar structure and meanings (proactive and diligent); In contrast, asymmetrical idioms like “风雨无阻”, “风雨” (wind and rain) combines with “**无阻**” (unimpeded) to form a cohesive meaning (of perseverance in facing challenges). If idioms are lexically accessed via their separate units, symmetrical idioms will be read faster than asymmetrical idioms. Alternatively, if idioms are accessed as a whole regardless of constituent comparability (i.e., as MCUs), then reading times should be similar. Using the boundary paradigm, we manipulated preview (identities or pseudocharacters) of each constituent. Consistent patterns were observed for both idiom types with greater preview benefit for the second constituent when the first was an identity than a pseudocharacter preview, suggesting MCU processing.

Attentional Disengagement Differences in Young Children with Autism: A Comparative Eye-movement Study Using Static and Dynamic Stimuli

Li Zhou[1,2], Fuyi Yang[2], Valerie Benson[1]
[1]University of Central Lancashire, United Kingdom; [2]East China Normal University, Shanghai, China
vbenson3@uclan.ac.uk

Dysfunctional disengagement represents a crucial concern in children with Autism Spectrum Condition (ASC). Failure to disengage from attended stimuli has consequences for the development of everyday communication skills, and in the real world, stimuli are often dynamic as well as static. In this study, we recorded eye movements and investigated attentional disengagement in young Chinese children for both static and dynamic stimuli. Our approach employed gap-overlap paradigms (GOP) with stimuli consisting of static (Experiment 1: 44 ASC, 47 TD) or dynamic (Experiment 2: 26 ASC, 26 TD) geometric figures. Basic oculomotor function was intact in both groups. No significant group differences were observed for reflexive saccades and attentional orienting behaviour between ASC and TD children when using the classic GOP task. However, children with ASC consistently exhibited prolonged voluntary disengagement (longer saccade latencies) in a modified-overlap condition across both stimulus types. Furthermore, ASC children demonstrated increased delayed disengagement when presented with dynamic foveal stimuli consisting of repetitive motion compared to random motion patterns, and this effect was absent in TD children. These findings reflect how attentional biases to both static and repetitive dynamic stimuli impact upon disengagement, and hence have potential to influence future development of social cognition in ASC.

Diagnosis of Schizophrenia by Integrated Saccade Scores

Jiahui Zhu[1], Li Zhou[2], Chuan Shi[1]
[1]**Peking University Sixth Hospital, Peking University Institute of Mental Health, Beijing, China, NHC** Key Laboratory of Mental Health (Peking University), National Clinical Research Center for Mental Disorders (Peking University Sixth Hospital), Beijing, China.; [2]Faculty of Education, East China Normal University, Shanghai, China
2011110594@bjmu.edu.cn

We aim to estimate the diagnostic accuracy of integrated pro/antisaccade eye movement measurements to discriminate between healthy individuals and schizophrenic patients. We compared the eye movement performance of 85 healthy individuals and 116 schizophrenia-stable patients during prosaccade and antisaccade tasks. The difference eye movement measurements were accumulated by stepwise discriminant analysis to produce an integrated score. Finally, the diagnostic value of the integrated score was calculated by the receiver operating characteristic (ROC) area under the curve (AUC), and the best sensitivity and specificity were calculated based on the given cut-off values. Using discriminant analysis, an integrated score included the residual gain and latency (step) during the prosaccade test, the error rate, and the corrected error rate during the antisaccade test. We found that the integrated score could well classify schizophrenia patients and healthy individuals with an accuracy of 80.6%. In the ROC, Youden's index was 0.613 (sensitivity = 80.5%, specificity = 80.7%) and AUC was 0.869. Using only two saccade tasks to discriminate well between schizophrenia patients and healthy controls, the underlying neuropathologic mechanisms associated with abnormal saccades may provide insights into the intervention and diagnosis of schizophrenia.

Effects of Decoding and Linguistic Skills on Reading Fluency and Comprehension in Finnish 2nd and 3rd Graders

Raymond Bertram, Ida-Maria Martti
University of Turku, Finland
rayber@utu.fi

The impact of component skills on reading development depends on the depth of orthography (see, e.g., Florit & Cain, 2011). In an orthographically shallow language like Finnish decoding skills are supposedly easily acquired, leaving room for linguistic skills like vocabulary knowledge to have an early impact. The current eye movement study investigates to what extent reading fluency and reading comprehension of 2nd and 3rd graders depend on these different types of skills. Decoding skills of 40 second graders and 40 third graders were assessed by a pseudoword naming and word chain test, while linguistic skills were assessed by the Lexize vocabulary test (Salmela et al., 2021). Reading fluency and reading comprehension were assessed by a modified text from the MECO corpus (Siegelman et al., 2022). The results showed that across grades most of the explained variance in reading comprehension stemmed from vocabulary knowledge with only a minor role to play for decoding skills. The same pattern was discernable in most eye movement measures of reading fluency. The results indicate that in Finnish decoding skills have limited influence on reading comprehension and reading fluency and underline the pivotal role of vocabulary knowledge in the initial phases of reading development.
